# An expanded codebook of human transcription factor DNA-binding specificity

**DOI:** 10.1038/s41586-026-10798-9

**Published:** 2026-08-05

**Authors:** Arttu Jolma, Kaitlin U. Laverty, Ali Fathi, Ally W. H. Yang, Isaac Yellan, Ilya E. Vorontsov, Antoni J. Gralak, Judith F. Kribelbauer-Swietek, Sachi Inukai, Rozita Razavi, Mihai Albu, Alexander Brechalov, Zain M. Patel, Vladimir Nozdrin, Georgy Meshcheryakov, Andrey Buyan, Ivan Kozin, Sergey Abramov, Alexandr Boytsov, The Codebook Consortium, Arttu Jolma, Kaitlin U. Laverty, Ali Fathi, Ally W. H. Yang, Isaac Yellan, Ilya E. Vorontsov, Antoni J. Gralak, Sachi Inukai, Rozita Razavi, Mihai Albu, Alexander Brechalov, Zain M. Patel, Vladimir Nozdrin, Georgy Meshcheryakov, Ivan Kozin, Sergey Abramov, Alexandr Boytsov, Fedor A. Kolpakov, Vsevolod J. Makeev, Marjan Barazandeh, Zhenfeng Deng, Chun Hu, Samuel A. Lambert, Sara E. Pour, Mikhail Salnikov, Hong Zheng, Giovanna Ambrosini, Judith F. Kribelbauer-Swietek, Marie-Luise Plescher, Semyon Kolmykov, Ivan Yevshin, Nikita Gryzunov, Mikhail Nikonov, Arsenii Zinkevich, Katerina Faltejskova, Pavel Kravchenko, Vasilii Kamenets, Dmitry Penzar, Anton Vlasov, Aldo Hernandez-Corchado, Hamed S. Najafabadi, Xiaoting Chen, Quaid Morris, Matthew T. Weirauch, Oriol Fornes, Jan Grau, Ivo Grosse, Philipp Bucher, Bart Deplancke, Ivan V. Kulakovskiy, Timothy R. Hughes, Quaid Morris, Matthew T. Weirauch, Oriol Fornes, Vsevolod J. Makeev, Jan Grau, Ivo Grosse, Philipp Bucher, Bart Deplancke, Ivan V. Kulakovskiy, Timothy R. Hughes

**Affiliations:** 1 Donnelly Centre, University of Toronto, Toronto, Ontario Canada; 2 Computational and Systems Biology Program, Sloan Kettering Institute, Memorial Sloan Kettering Cancer Center, New York, NY USA; 3 Department of Molecular Genetics, University of Toronto, Toronto, Ontario Canada; 4 Vavilov Institute of General Genetics, Russian Academy of Sciences, Moscow, Russia; 5 Laboratory of Systems Biology and Genetics, Institute of Bioengineering, School of Life Sciences, École Polytechnique Fédérale de Lausanne, Lausanne, Switzerland; 6 Swiss Institute of Bioinformatics, Lausanne, Switzerland; 7 Department of Biological Sciences, University of Southern California, Los Angeles, CA USA; 8 Faculty of Bioengineering and Bioinformatics, Lomonosov Moscow State University, Moscow, Russia; 9 Institute of Protein Research, Russian Academy of Sciences, Pushchino, Russia; 10 Institute of Biochemistry and Genetics, Ufa Federal Research Centre of Russian Academy of Sciences, Ufa, Russia; 11 Altius Institute for Biomedical Sciences, Seattle, WA USA; 12 Center for Autoimmune Genomics and Etiology, Cincinnati Children’s Hospital Medical Center, Cincinnati, OH USA; 13 Divisions of Allergy and Immunology, Biomedical Informatics, Human Genetics, and Developmental Biology, Cincinnati Children’s Hospital Medical Center, Cincinnati, OH USA; 14 Department of Pediatrics, University of Cincinnati College of Medicine, Cincinnati, OH USA; 15 Department of Medical Genetics, Centre for Molecular Medicine and Therapeutics, BC Children’s Hospital Research Institute, University of British Columbia, Vancouver, British Columbia Canada; 16 Institute of Computer Science, Martin Luther University Halle-Wittenberg, Halle, Germany; 17 Department of Computational Biology, Sirius University of Science and Technology, Sirius, Russia; 18 Bioinformatics Laboratory, Federal Research Center for Information and Computational Technologies, Novosibirsk, Russia; 19 Bioinformatics Competence Center, Université de Lausanne, Lausanne, Switzerland; 20 Biosoft, Novosibirsk, Russia; 21 Institute of Organic Chemistry and Biochemistry of the Czech Academy of Sciences, Prague, Czech Republic; 22 Computer Science Institute, Faculty of Mathematics and Physics, Charles University, Prague, Czech Republic; 23 Max Planck Institute of Biochemistry, Planegg, Germany; 24 TUD Dresden University of Technology, Center for Molecular and Cellular Bioengineering (CMCB), Biotechnologisches Zentrum (BIOTEC), Dresden, Germany; 25 Department of Human Genetics, McGill University, Montréal, Quebec Canada; 26 Victor P. Dahdaleh Institute of Genomic Medicine, Montréal, Quebec Canada; 27 Present Address: Cancer Research UK National Biomarker Centre, University of Manchester, Manchester, UK

**Keywords:** Gene regulation, Transcriptional regulatory elements, Transcriptomics

## Abstract

Gene expression is regulated by transcription factors (TFs), which recognize specific DNA sequence motifs. Several hundred putative human TFs, identified mainly by an apparent DNA-binding domain, lack known binding motifs^1^. Furthermore, even for well-characterized TFs, it remains controversial the degree to which motifs accurately reflect binding sites in living cells^2^. Here we describe a systematic effort (‘Codebook’) to determine the sequence specificity of 332 putative and poorly characterized human TFs. More than 4,000 independent experiments, encompassing multiple in vitro and in vivo assays, produced motifs for just over half (177; 53%) of the TFs, of which most are associated with only a single protein. These results extend the vocabulary of sequence recognition encoded by human TFs by around 130 distinct motifs. Moreover, binding motifs identified in vitro are strongly enriched in cellular binding sites. Collectively, the data reveal tens of thousands of previously unknown, conserved and direct TF-binding sites across the human genome. These sites are concentrated in promoter regions and are predictive of gene expression. In summary, this new codebook provides an important step forward in decoding the human genome.

## Abstract (web summary)

Results from a panel of assays that analyse different aspects of DNA sequence specificity reveal more than 100 new motifs to aid the characterization of putative human transcription factors.

## Main

A 2018 survey of putative human TFs concluded that over one-quarter of the estimated 1,600 TFs lacked established binding motifs^1^. This proportion represents a striking deficit given that most of the conserved DNA in the human genome is noncoding^3^ and that a primary hypothesis for the function of noncoding DNA is gene regulation^4^. Moreover, most of the uncharacterized and putative TFs are not close paralogues of any established TF. Thus, despite possessing either literature evidence of DNA binding or protein domains that would typically bind DNA (DNA-binding domains (DBDs)), their binding motifs cannot be readily inferred. However, it cannot be excluded that these putative TFs lack DNA sequence specificity entirely.

TF DNA-binding motifs are commonly modelled as position weight matrices (PWMs), which describe the relative preference of a TF for each nucleotide base pair in the binding site^5,6^. Different methods for measuring TF binding, and for deriving PWMs from the resultant data, have different inherent limitations and biases^5^. There is also long-standing controversy regarding the contribution of the inherent sequence specificity of a TF to its cellular binding relative to the influence of chromatin and cofactors^2^. These uncertainties represent fundamental hurdles for the analysis of gene regulation and for a myriad of related tasks in genome analyses, including the interpretation of conserved genomic elements and sequence variants.

To address these issues, we analyse a large majority of the poorly characterized human TFs^1^ (defined here as having no confidently known binding motif) and several dozen previously studied control TFs^7,8^ using a panel of assays that provide different perspectives on DNA sequence specificity. We refer to this international collaborative project as the ‘Codebook–GRECO-BIT Collaboration’. The reagent set and laboratory experiments were initiated as the ‘Codebook project’, which alludes to the fact that TFs decode individual ‘words’ in the genome. Meanwhile, the Benchmarking Initiative by the Gene Regulation Consortium, GRECO-BIT rooted in GRECO^9^, was engaged for much of the data analyses.

In this paper, we present an overview of the Codebook data, major outcomes of the study and examples of prevalent phenomena and applications. We discuss the following findings:

- Just over half of the 332 putative (that is, Codebook) TFs (177) display DNA sequence specificity, most with the same motifs observed in vitro and in living cells.
- The resulting 177 motifs are largely different from one another and from motifs of previously studied human TFs.
- Tens of thousands of genomic binding sites for these TFs display evidence of purifying selection at the motif matches, which indicates that they are functional.
- These conserved binding sites are abundant in the promoters of coding genes, and promoter-binding sites of the Codebook TFs are predictive of gene expression across tissues and cell types.
- Many TFs bind to genomic dark matter, such as transposons, or are derived from transposons, which suggests that they may have had roles in adaptive evolution.

Accompanying manuscripts^10–13^ and the Supplementary Information provide greater technical detail and depth, including biological findings, new assays and TF families of note, as well as methods for identifying and benchmarking PWMs.

## Overview of the Codebook project

**Fig. 1:**
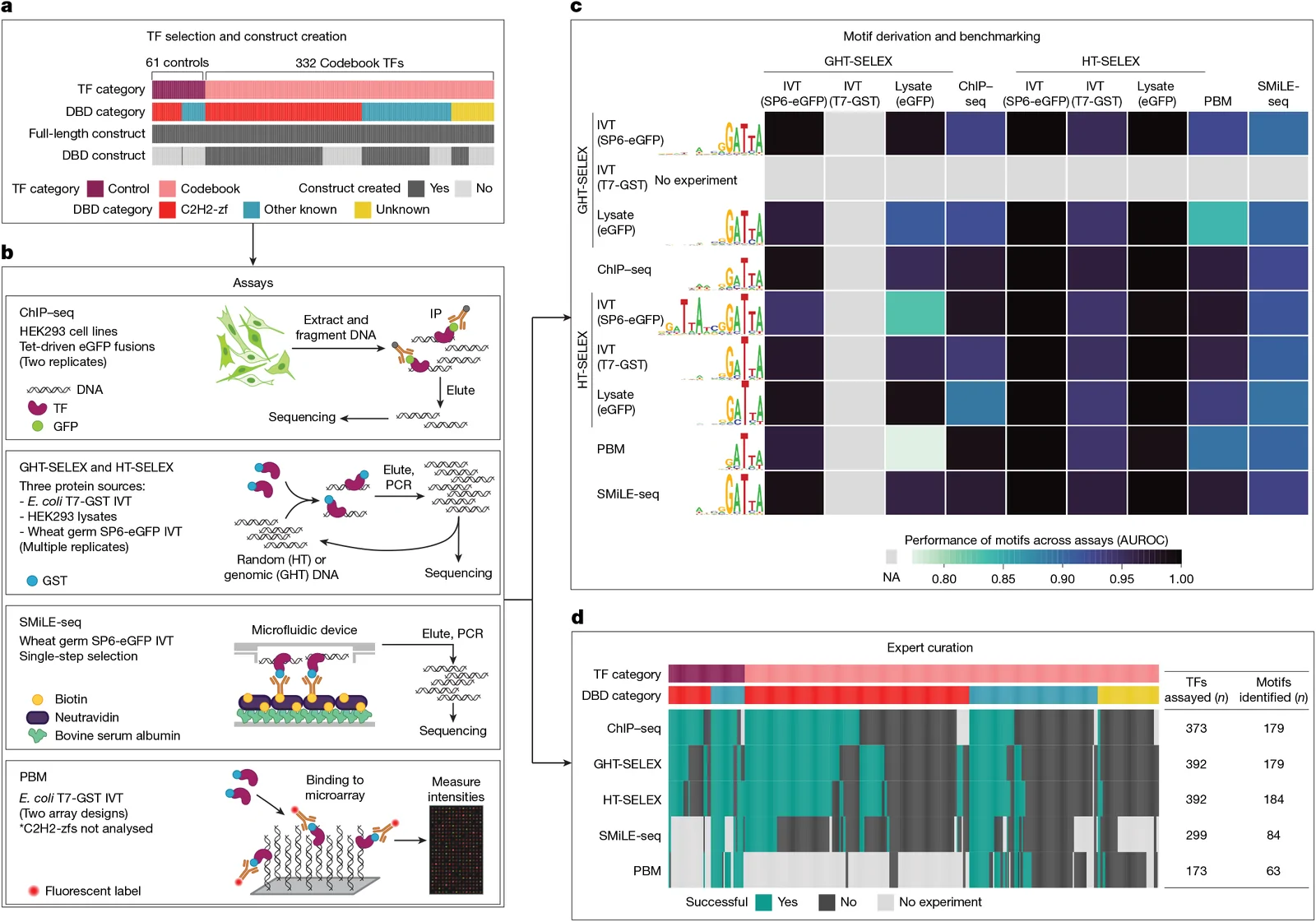
Codebook project overview. **a**, Categories of 393 TFs assayed and corresponding constructs. **b**, Graphical summary of the assays used in the study. **c**, Example of performance (as AUROC values) of the best-performing PWM for TPRX1, for each combination of experiment type: one for PWM derivation (rows) and one for PWM testing (columns). **d**, Heatmap of successful experiments for all 393 TFs across all experiment types. *E. coli*, *Escherichia coli*; IP, immunoprecipitation; IVT, in vitro transcription or translation; NA, not applicable; AUROC, area under the receiver operating characteristic curve. Source data

Figure 1 provides a schematic of the Codebook project. We analysed 393 proteins (332 putative Codebook TFs and 61 control TFs) (Supplementary Table 1) using up to 5 different assays that encompass both in vitro and living cellular contexts: HT-SELEX, SMiLE-seq, protein binding microarrays (PBMs), ChIP–seq (with inducible tagged transgenes in HEK293 cells) and GHT-SELEX, a variant of HT-SELEX in which the selections are performed using fragmented genomic DNA^10^ instead of random sequences. The GHT-SELEX, SMiLE-seq and ChIP–seq data generated significant and extensive biological insights on their own, which are detailed in accompanying manuscripts^10,11,13^. Highlights and joint conclusions are summarized here. Different assays accommodate different protein tags and expression systems, and many TFs were analysed using multiple constructs (for example, full-length and DBDs) and multiple expression systems, which led to variable numbers of constructs analysed and experiments per protein. The study used 622 and 93 protein-coding inserts cloned into 1,267 and 118 protein expression constructs for the Codebook TFs and controls, respectively (Supplementary Tables 2 and 3). In total, we performed 4,804 experiments: 4,377 for Codebook proteins and 427 for control TFs (Supplementary Table 4).

**Extended Data Fig. 1:**
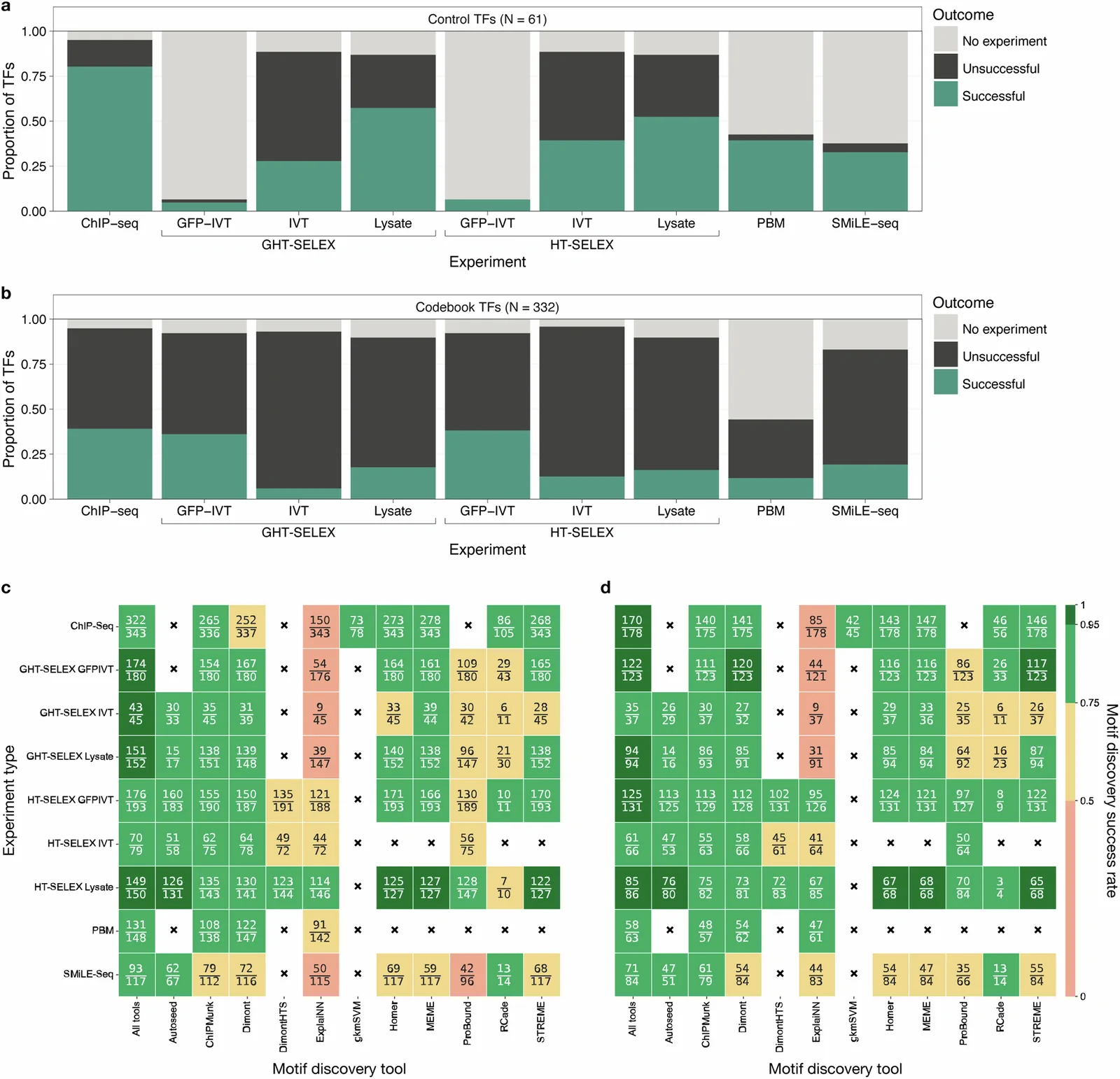
Motif discovery success rates. **a**, **b**, Proportion of control (**a**) and Codebook (**b**) TFs for which a motif was successfully identified across experiment types. GHT-SELEX and HT-SELEX experiments are separated according to protein expression system. The proportion of untested TFs is also indicated. **c, d**, Rate of successful motif discovery across experiment types and motif discovery tools. Numerator in each cell represents the number of successes (cases of correctly identified motifs), and denominator represents the number of experiments (**c**) or TFs (**d**) for a particular combination of a tool and an assay. The first column in **c** and **d** displays the joint estimates across all tools. IVT: in vitro transcription, TF: transcription factor. Source data

Because the Codebook proteins had no known motif, the process of determining which experiments were successful was, by necessity, coupled to the process of deriving motifs from each of the datasets, as described in detail in a separate manuscript^12^. In brief, if we obtained similar motifs for the same protein from two or more different experimental platforms, and these motifs were predictive of data across platforms, then we considered the experiments that produced these motifs to be successful. In total, 1,073 experiments for Codebook proteins and 280 for controls were deemed successful (Supplementary Table 4). We obtained motifs for 177 out of 332 Codebook proteins and 58 out of 61 control TFs using this process, values that indicated a low overall false-negative rate. The number of Codebook proteins deemed successful in a particular assay was 130 for ChIP–seq (41% of those tested), 144 for HT-SELEX (44% of those tested), 139 for GHT-SELEX (42% of those tested), 64 for SMiLE-seq (23% of those tested) and 39 for PBMs (27% of those tested) (Extended Data Fig. 1a,b and Supplementary Table 1). For each of the proteins, we selected a single representative PWM that scored highly across data types for use in subsequent analyses (Methods and Supplementary Table 5).

**Extended Data Fig. 2:**
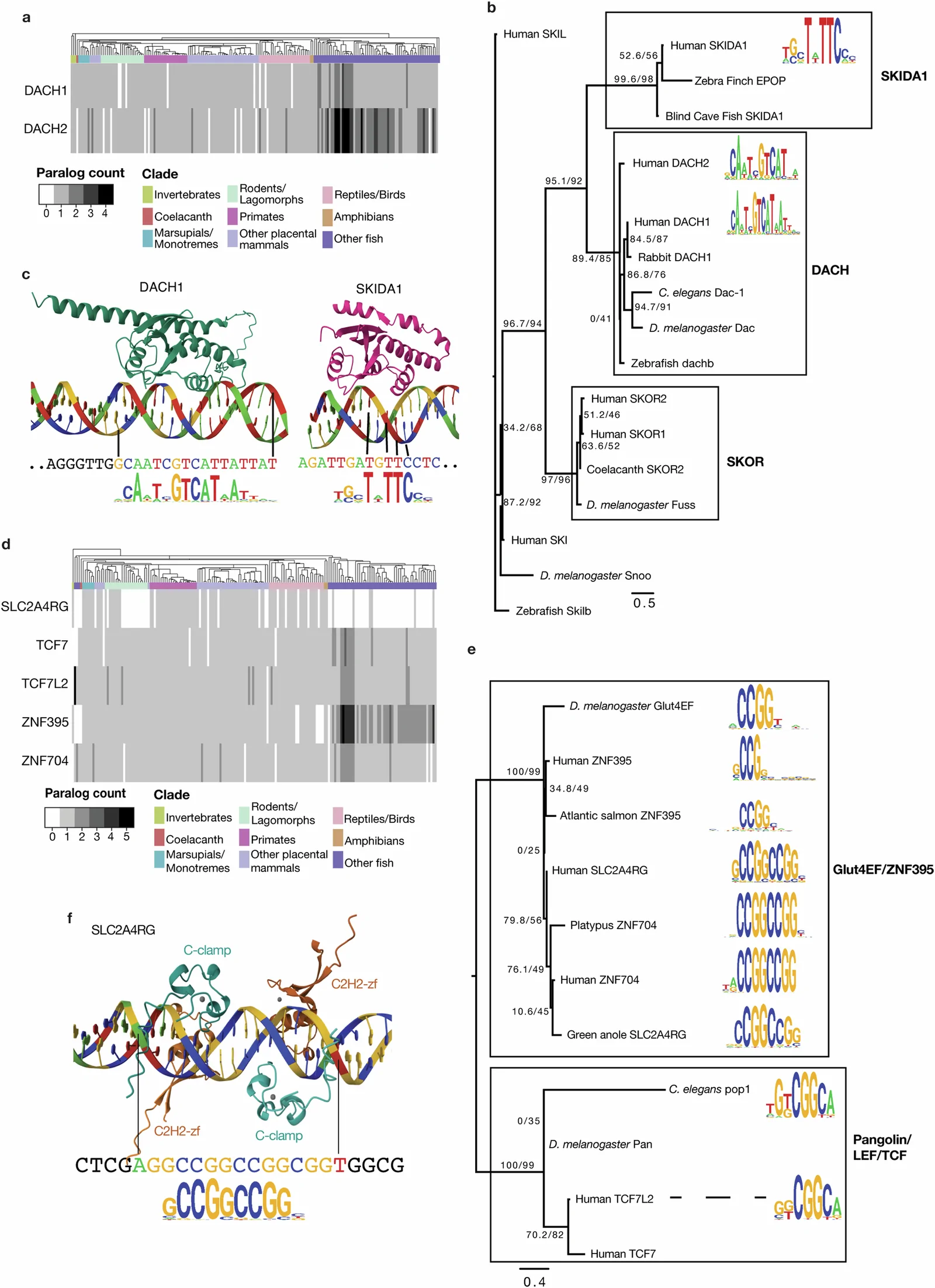
Undercharacterized DNA-binding domains. Overview of new motifs for undercharacterized TF families. **a**, Number of DACH1 and DACH2 orthologs (union of one-to-one and one-to-many) across Ensembl v111 vertebrates and selected invertebrates. Species order reflects the Ensembl species tree. **b***,* Sequence logos (from the main Codebook project, and follow-up study described in the Supplementary Discussion) and sequence relationships of human and representative animal SKI/SNO/DAC domains. All motifs from successful HT-SELEX assays are shown, and a representative set of the DBDs tested is included in the phylogenetic tree. Tree is an unrooted maximum-likelihood phylogram from IQTree^79^ using parameters --alrt 1000 -B 1000 (1000 replicates to compute SH-aLRT (a branch support value from 0−100%), 1000 replicates for ultrafast bootstrap), and automatic best-fit model selection with ModelFinder. Branch values are SH-aLRT/ultrafast bootstrap. **c**, AlphaFold3-predicted structure of the DACH1 SKI/SNO/DAC region (residues 130 – 390) bound to an HT-SELEX ligand sequence with a high-scoring PWM match. **d**, Number of orthologs (same counting process as **a**) for human C-clamp proteins. **e**, Sequence logos (from the main Codebook project and follow-up study) and sequence relationships of human and representative animal C-Clamp domains (*ZNF704 motif from^80^). The tree was produced identically to the tree in **b**. **f**, AlphaFold3-predicted structure of two full-length SLC2A4RG proteins bound to a CTOP sequence with flanking sequences (chr17:48,048,369-48,048,401), and four Zn^**2+**^ ions (grey). The remainder of the proteins (beyond the C-clamp and C2H2-zf domains) are hidden, for visual simplicity. DBD: DNA-binding domain. DNA-protein structures were predicted with AlphaFold3 (available under Creative Commons licence CC-BY-4.0) and visualized using Mol*^81^. Source data

The 177 successful Codebook TFs were dominated by the large and diverse C2H2 zinc finger (C2H2-zf) class, for which 67% (121 out of 180) produced motifs. C2H2-zf proteins can bind RNA, protein or other ligands^14^. Our finding indicates that most of the Codebook C2H2-zf proteins are DNA-binding proteins, although it does not exclude other functions. Among the Codebook TFs with other DBD types, roughly half (49%; 50 out of 103) also produced motifs. The Codebook TFs also encompassed 49 proteins that lack a well-established DBD, which were included owing to literature evidence for DNA sequence specificity. Among these, only six (12%) produced motifs. After closer inspection, all six do, in fact, contain plausible DNA-binding regions or DBDs (Supplementary Note 1). For example, DACH1 and DACH2, which recognize the same motif, are homologues of *Drosophila* *Dachshund* and share the SKI/SNO/DAC domain. It has long been controversial whether this domain binds DNA^15^. Subsequent to the main Codebook study, we tested all five additional human SKI/SNO/DAC domain-containing proteins by HT-SELEX, and this experiment identified a motif for one: SKIDA1. This result provides further support for SKI/SNO/DAC acting as a DBD (Extended Data Fig. 2a–c and Supplementary Note 1).

**Extended Data Fig. 3:**
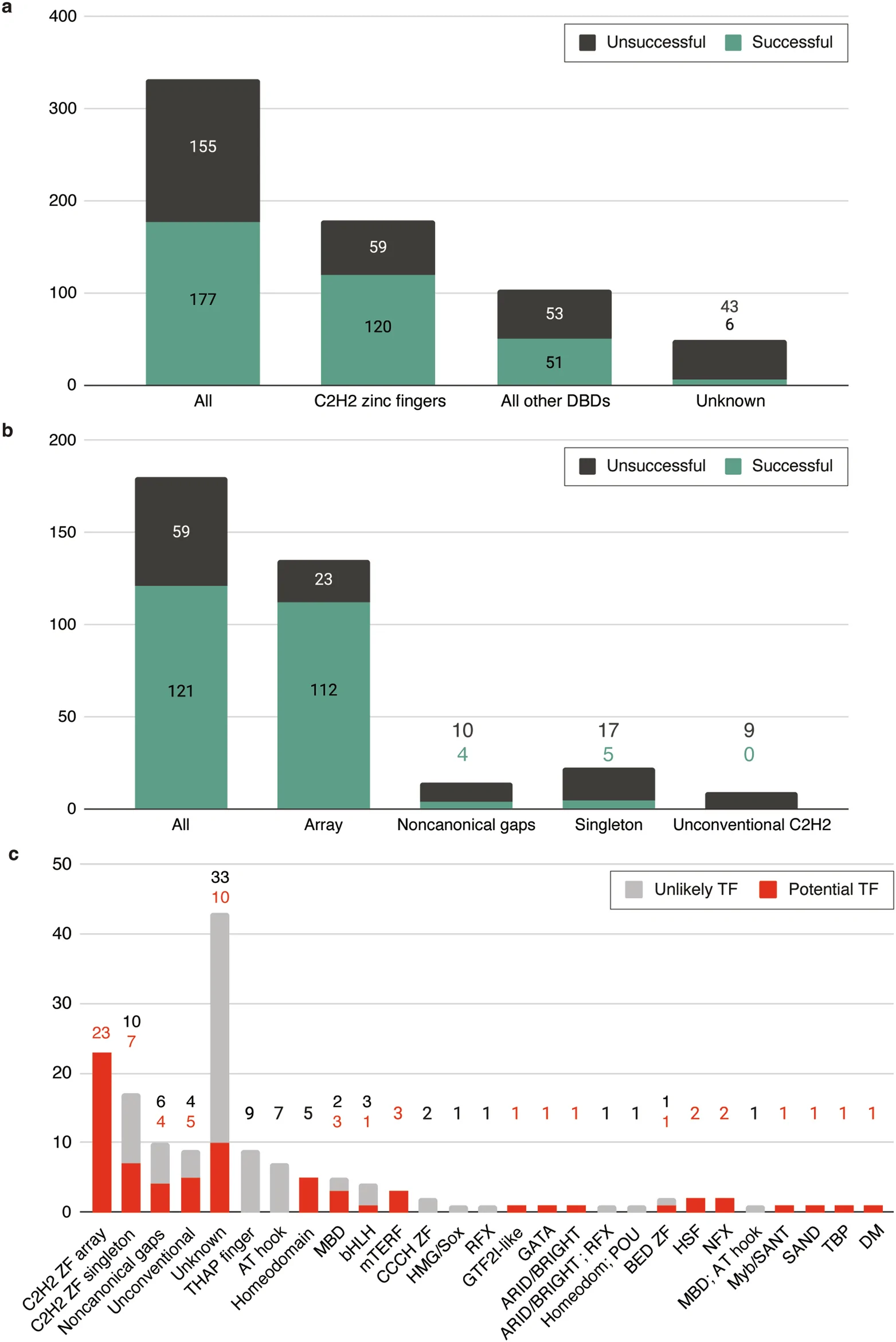
Description and re-assessment of proteins that did not yield motifs (i.e. “unsuccessful”). **a**, Number of successful and unsuccessful Codebook proteins by category. **b**, Number of successful and unsuccessful C2H2-zf proteins categorized based on: whether a protein has an array of at least two DBDs separated by 3-10 AA; has multiple C2H2-zf domains but with other kinds of spacings; has only a single domain; or if it has a potential C2H2-zf domain based on literature but none are predicted by our standard HMM based prediction. **c**, Summary of re-assessment (see Supplementary Table 6 and Supplementary Discussion for details) of unsuccessful proteins by structural class or category. Colors indicate if the protein is still assessed as a potential TF (red) and is therefore a potential false negative, or if it is now assessed as an unlikely TF (gray). TF: transcription factor, DBD: DNA-binding domain, HMM: hidden Markov model. Source data

Nearly half of the Codebook proteins did not produce motifs (PWMs) that passed our success criteria. False negatives could arise from the lack of an obligate binding partner, a requirement for epigenetically modified DNA, a lack of requisite post-translational modification in our experiments or other limitations of the methods. We manually re-assessed each of these proteins that did not produce a motif and deemed that the majority (83 out of 155) probably represent true negatives. C2H2-zf proteins with a single C2H2-zf domain, or composed only of unusual C2H2-zf domains, typically did not produce motifs in our assays. Nor did those that lack the canonical spacing of C2H2-zf domains that supports binding of tandem C2H2-zf arrays to longer sites^16^ (Extended Data Fig. 3, Supplementary Discussion and Supplementary Table 6). This result suggests that these C2H2-zf domains may have roles other than in DNA binding. Some subtypes of other bona fide DBD classes also lack sequence specificity (for example, HMG), and we found almost two dozen such cases. Moreover, the 155 putative TFs that did not produce a motif in any assay were enriched for those with no known DBD (of which 43 out of 49 failed). Among these, 33 have no evidence for DNA binding beyond a single study, most of which were published over two decades ago.

## Cellular binding sites and SNVs

**Fig. 2:**
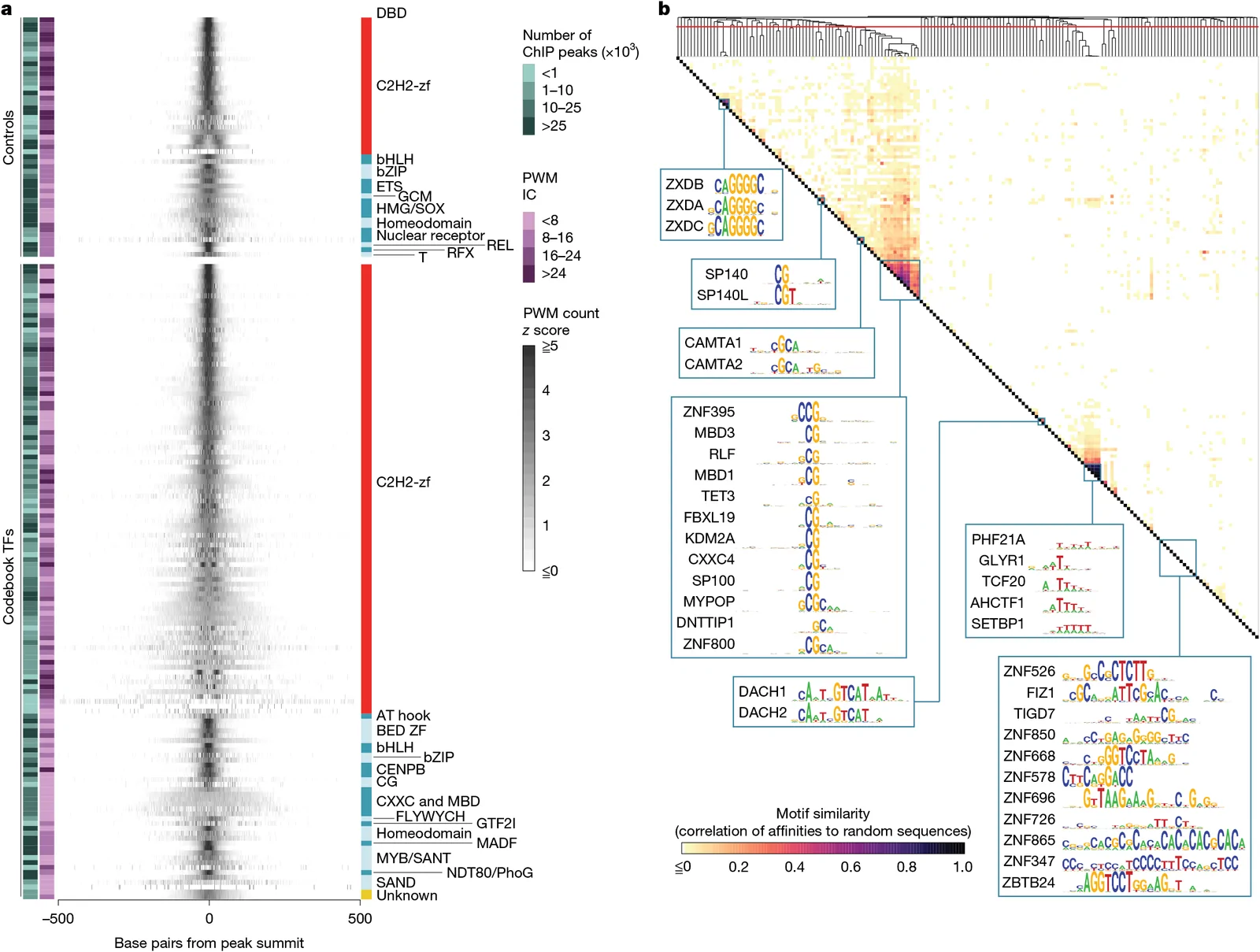
Motif relationships to cellular binding sites and to other motifs. **a**, Heatmap of the standardized count of representative PWM matches at each base ±500 bp from ChIP–seq peak summits of the TF. PWM matches were identified using MOODS^51^ (*P* < 0.001) and aggregated for each TF at each base position. For visualization, counts were standardized (*z* score across all base positions) for each TF. PWM IC and the number of ChIP–seq peaks for each TF are indicated on the left. Control and Codebook TFs are separated and grouped by DBD type. **b**, Symmetric heatmap of the similarity between representative PWMs for Codebook TFs, clustered by PCC values with average linkage. PWM similarity is the correlation between pairwise affinities to 150,000 random sequences of length 100, as calculated using MoSBAT^25^. The red line across the dendrogram indicates the clustering threshold determined by the optimal silhouette value^52^. Pullouts and labels illustrate specific points in the main text. IC, information content; PCC, Pearson correlation coefficient. Source data

The Codebook project generated technically successful ChIP–seq data for 130 out of the 177 successful Codebook TFs^11^, which enabled us to gauge the use of the motif in a cellular context. There was clear enrichment of motif matches in ChIP–seq peaks, which indicated that the intrinsic sequence specificities of the TFs are used in the selection of cellular binding sites (Fig. 2a). Some TFs displayed motif enrichment in a wide region around the peak summit due to multiple repeated motif matches. Notably, this phenomenon is particularly common at CpG island promoters (discussed below). An accompanying manuscript^10^ demonstrates that genomic binding in vitro and in vivo are more similar than generally thought. This conclusion could only be reached through analyses of both GHT-SELEX and ChIP–seq data coupled with optimization of PWM derivation and scanning methods.

**Extended Data Fig. 4:**
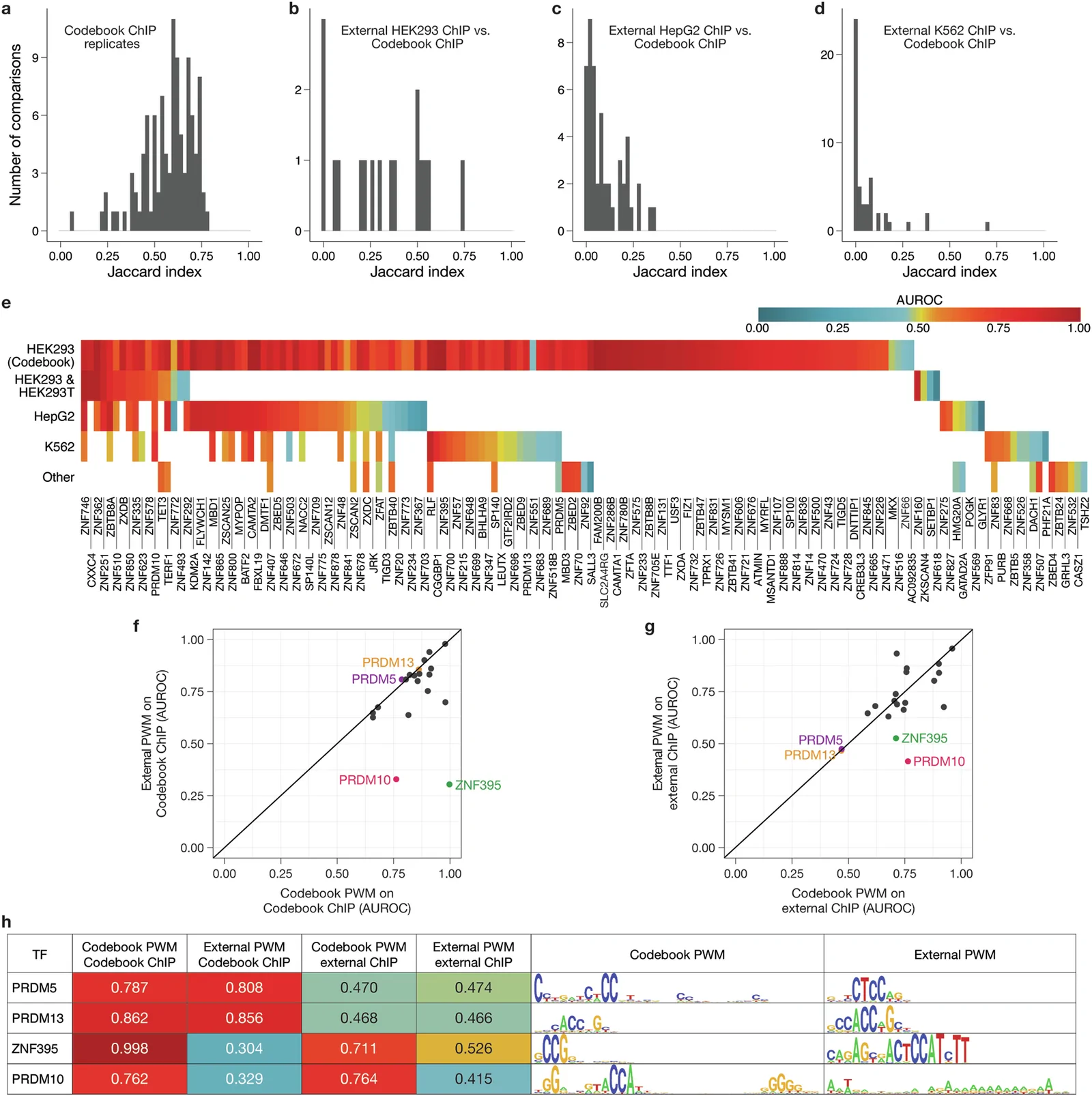
Comparison of Codebook ChIP-seq datasets and PWMs to external ChIP-seq datasets and PWMs. **a-d**, Histograms of optimized Jaccard indices measuring the overlap between ChIP-seq peak sets for the same TF: **a**, Codebook ChIP-seq replicates; **b, c, d**: Codebook ChIP-seq vs. external ChIP-seq performed in HEK293 cells (**b**), HepG2 cells (**c**), or K562 cells (**d**). Control TFs are not included. **e**, AUROC values for expert-curated Codebook PWMs (columns), discriminating ChIP-seq peaks vs. random genomic background loci. Rows show different cell types. **f, g**, comparison of Codebook and external PWMs at the task of discriminating ChIP-seq peak sets from random sequences (as in **e**), for the 20 TFs that have Codebook ChIP-seq data, a Codebook PWM, external ChIP-seq data, and an external PWM. **f**, Performance of Codebook PWM vs. the best performing external PWM on Codebook ChIP-seq data. **g**, Performance of Codebook PWM vs. the best performing external PWM on external ChIP-seq data. The four TFs with an AUROC of <0.5 on either axis of either plot are highlighted. **h**, Sequence logos for the four TFs highlighted in **f** and **g**. All expert-curated Codebook PWMs displayed are supported by ChIP-seq, GHT-SELEX, HT-SELEX, and SMiLE-seq data. PWM: position weight matrix, TF: transcription factor, AUROC: area under the receiver operating characteristic. Source data

The Codebook project was conducted over a period of nearly 6 years, and during this time, several large-scale studies aimed at systematic ChIP–seq analyses of human TFs (for example, ENCODE) were published, which mainly used cell lines other than HEK293 (refs. ^17–19^). Peaks from these external datasets overlapped only partially with the Codebook ChIP–seq peaks for the same TF. This result is expected because chromatin and cofactors can affect site accessibility and selection (Extended Data Fig. 4a–d and Supplementary Table 7). Nonetheless, Codebook PWMs were generally able to discriminate between binding sites in these other ChIP–seq data and random sequences (median area under the receiver operating characteristic curve (AUROC) of 0.71 on HEK293 cells, and slightly lower on other cell types). These results illustrate that the identified motifs are predictive across studies and cell types (Extended Data Fig. 4e).

**Extended Data Fig. 5:**
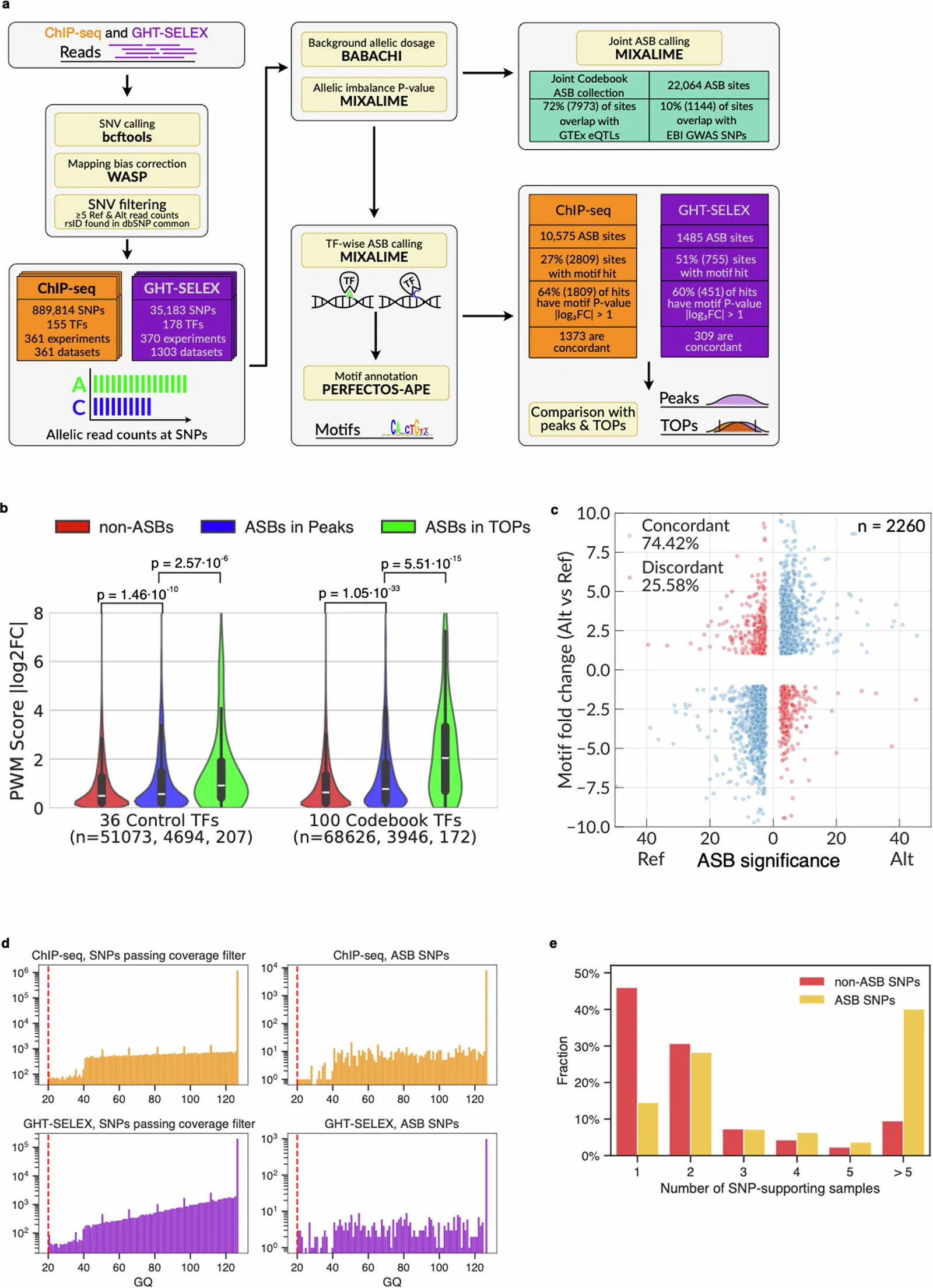
Identifying allele-specific TF binding sites in Codebook ChIP-seq and GHT-SELEX data. **a**, Principal scheme of the allele-specific analysis. **b**, Distribution of PWM score P-value ratio (fold change), |log_2_ FC|, between alleles for non-ASBs (FDR ≥ 5%), ASBs (FDR < 5%) in peaks, and ASBs in TOPs (FDR < 5%). *Left*, 36 positive control TFs; *Right*, 100 Codebook TFs. P-values: two-sided Mann-Whitney U test. Center line: median; box limits: upper and lower quartiles; whiskers: minimum and maximum values within the 1.5x interquartile range. For the 36 control TFs, the number of tested PWM hits (n) is 51,073 for non-ASBs, 4,694 for ASBs in peaks, and 207 for ASBs in TOPs. For the 100 Codebook TFs, the corresponding values are 68,626, 3,946, and 172. **c**, Motif concordance of Codebook ASBs. X-axis: ASB allelic preference and significance (-log_10_ FDR), *left*: preference for the reference allele (Ref), *right*: preference for the alternative allele (Alt). Y-axis: PWM score P-value ratio (log_2_ FC) between Alt and Ref. The plot shows only ASBs with |log_2_FC| > 1. **d**, Genotype quality (GQ) of SNPs called from ChIP-Seq (top) and GHT-SELEX (bottom) data. X-axis: GQ, Y-axis: the number of filtered-by-coverage candidate SNPs or ASB SNPs. The red dashed line at 20 is the default GQ threshold typically used to filter SNP calls. The majority of candidate SNPs and ASB SNPs have GQ much higher than the default threshold. **e**, The number of SNP-supporting ChIP-Seq or GHT-SELEX datasets for ASB and non-ASB SNPs. Due to file size limitations, the complete source data for this figure have been deposited into GitHub. See Data Availability for the link. The majority of ASB SNPs are supported by two or more datasets. ASB: allele-specific binding site, FC: fold change, SNP: single-nucleotide polymorphism, TOP: triple-optimized overlap.

Sequence variants, such as single-nucleotide variants (SNVs), can disrupt TF-binding sites, which will affect gene regulation^20^. Analyses of allelic read imbalance^21^ in ChIP–seq and GHT-SELEX peak sets (Extended Data Fig. 5 and Supplementary Table 8) revealed a total of 12,060 apparent allele-specific binding (ASB) events at 10,009 common single-nucleotide polymorphisms (SNPs) for 190 individual TFs at 5% false-discovery rate (FDR). Notably, for highly motif-disrupting variants (with at least twofold difference in *P* values between PWM predictions for the reference and the alternative alleles), the allelic preference of ASBs was concordant with PWM predictions for 1,682 out of 2,260 (74%) of ASBs. Thus, although the specific ASBs detected experimentally are limited to SNPs present in HEK293 cells, the Codebook motifs can be applied to predict differential TF binding to any other sequence variants. When considering aggregated ASBs across all tested TFs and experiments (Methods), the corresponding SNPs were enriched for previously characterized ASBs^22^ (odds ratio = 2.4, *P* = 3.90 × 10^–162^), disease-associated SNPs^23^ (odds ratio = 1.35, *P* = 3.79 × 10^–18^) and GTEx expression quantitative trait loci^24^ (odds ratio = 1.34, *P* = 1.77 × 10^–41^) (two-sided Fisher’s exact test) (Supplementary Table 9).

## Motif diversity and degeneracy

**Extended Data Fig. 6:**
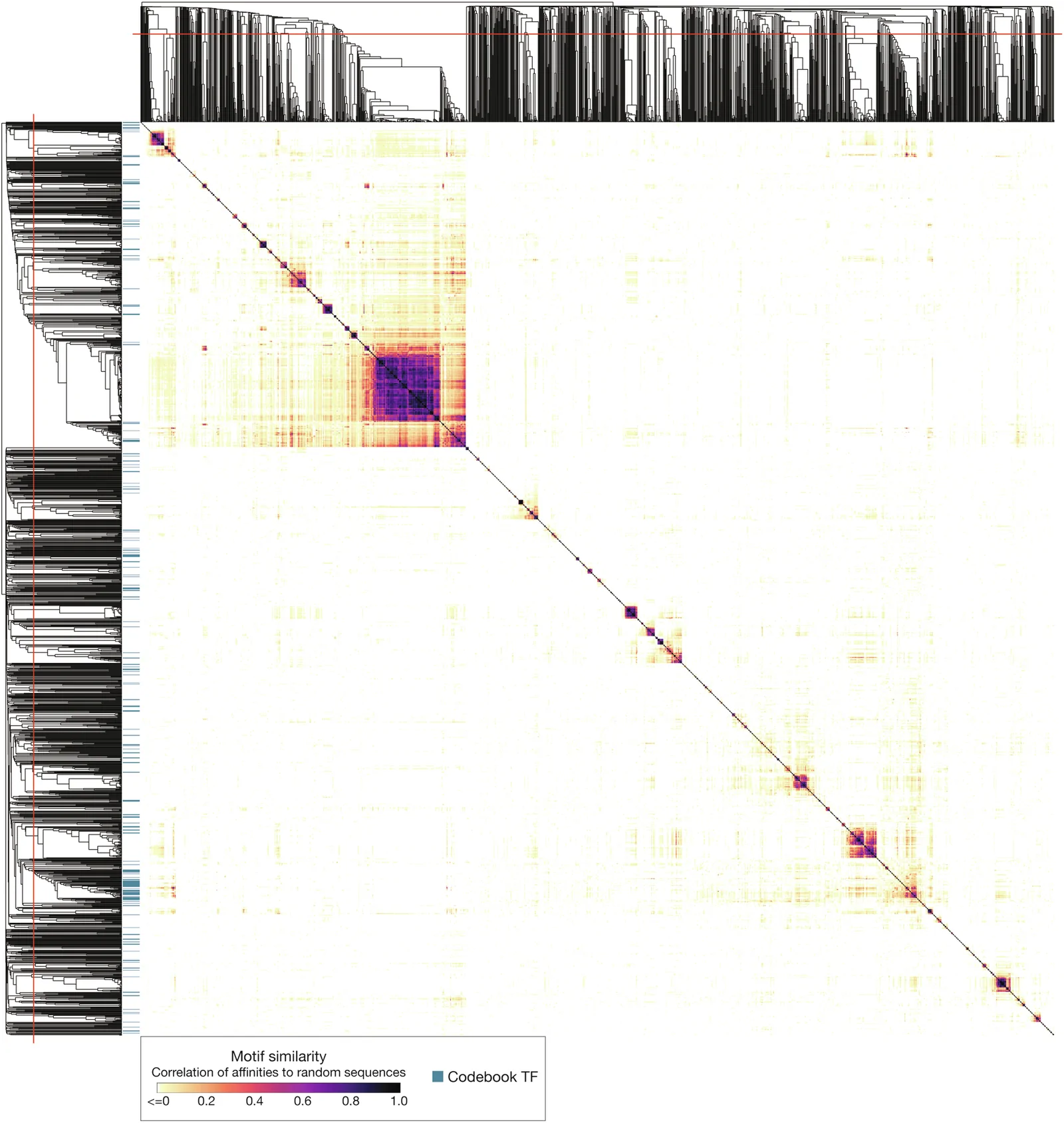
Similarity of TF motifs. Symmetric heatmap displays the similarity between 1,582 PWMs clustered by Pearson correlation with average linkage. The PWM set is composed of the 177 representative PWMs for Codebook TFs, and 1,405 PWMs for 1,211 TFs with previously identified binding specificities. The set of PWMs for non-Codebook TFs was retrieved from Lambert et al. ^1^. PWM similarity is the correlation between pairwise affinities to 150,000 random sequences of length 100 bp, as calculated by MoSBAT^25^. The red line across the dendrogram indicates the clustering threshold (see Fig. 2b, Methods) determined by the optimal silhouette value^52^. PWMs and cluster membership are in Supplementary Table 10. PWM: position weight matrix, TF: transcription factor. Source data

The Codebook protein sequences were typically not closely related to each other or to those of known TFs, which suggests that their DNA sequence preferences may also differ^1^. Figure 2b shows an overview of similarity^25^ among the representative Codebook PWMs, which illustrates that most of the Codebook TF motifs are indeed distinct. Most are also dissimilar to any other known PWM^12^ (Extended Data Fig. 6). This result is partially explained by the large number of C2H2-zf proteins in the dataset, which differ in their DNA-contacting ‘specificity residues’^26^. Small clusters along the diagonal in Fig. 2b primarily corresponded to the handful of paralogues analysed (for example, SP140 and SP140L, DACH1 and DACH2, CAMTA1 and CAMTA2, and ZXDA, ZXDB and ZXDC). Furthermore, the middle of Fig. 2b contains a set of eight TFs for which the motif is dominated by a single CG sequence. Five of them (CXXC4, FBXL1, TET3, MBD1 and KDM2A) contain CXXC-zf domains, which are expected to bind clusters of unmethylated CpGs^27^. Similarly, in the lower right is a group of five AT-hook proteins that prefer AT-containing sequences. Overall, however, the 177 Codebook TFs encompassed 129 distinct motifs (Fig. 2b and Methods). When we performed motif clustering jointly with previously catalogued, representative motifs for 1,211 human TFs (that is, 7.8-fold more TFs), we obtained only 4.75-fold more distinct motif clusters (613), of which 92 (15%) contained only Codebook proteins (Extended Data Fig. 6 and Supplementary Table 10). These numbers are robust to methodology. That is, the use of a different public motif database, a different similarity metric and a different clustering procedure led to a similar outcome, with a total of 653 motif clusters, 135 (21%) of which contained only Codebook motifs (Methods and Supplementary Table 11). Thus, the Codebook data expand the lexicon of human TFs by at least 15%, providing around 130 previously unknown motifs.

**Extended Data Fig. 7:**
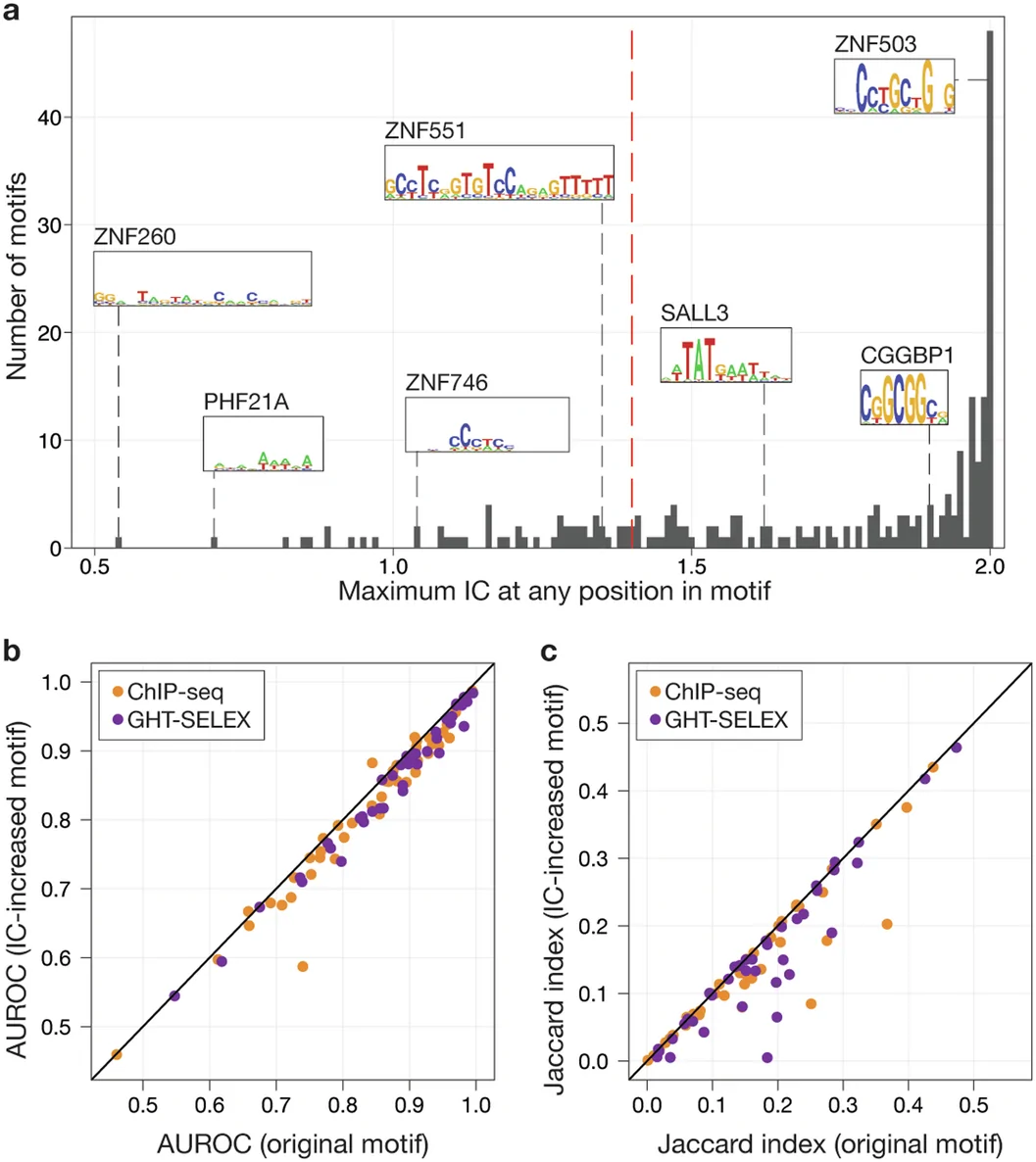
Motif degeneracy analysis. **a**, Histogram displays the maximum IC for any position within the representative PWM for all Codebook and control TFs. Logos are shown for TFs at various maximum positional IC values, for illustration. Red dashed line indicates an IC of 1.4. **b**, and **c**, comparison of original PWMs to IC-increased PWMs for the 52 TF PWMs for which no base position exceeded an IC of 1.4. **b**, AUROC values for original vs. IC-increased PWMs, discriminating ChIP-seq or GHT-SELEX peaks vs. random genomic background loci. **c**, Maximum Jaccard index for ChIP-seq or GHT-SELEX peak sets; using the approach described for optimized TOPs in Methods, for original vs. IC-increased PWMs. PWM: position weight matrix, IC: information content, TOP: triple-optimized overlap regions, AUROC: area under the receiver operating characteristic. Source data

For dozens of TFs, the representative PWM, selected for its highest accuracy across datasets, contained few or no positions at which a specific base was absolutely required (Extended Data Fig. 7a). This property is referred to as degeneracy, and it causes ‘flat’ motifs with low information content (IC), which might be expected to represent low specificity^6^. Artificially increasing the IC (that is, ‘unflattening’ the sequence logo and therefore increasing the apparent specificity of the low-IC representative PWMs), however, almost universally reduced motif performance (Extended Data Fig. 7b,c). This finding indicates that degeneracy is required for maintaining prediction accuracy across data types. We also found that overall, IC is not predictive of motif performance^12^. It is counterintuitive that degeneracy (that is, lower apparent specificity) would lead to better predictive capacity, but similar findings in other studies^28^ support the validity of the result. For particular TFs that can recognize more than one DNA pattern, degeneracy may be a consequence of forcing a single PWM to represent multiple related motifs^12^. It is also conceivable that it is beneficial to evolutionary processes if TFs have the ability to tolerate mutations in binding sites.

## Conservation of genomic binding sites

**Fig. 3:**
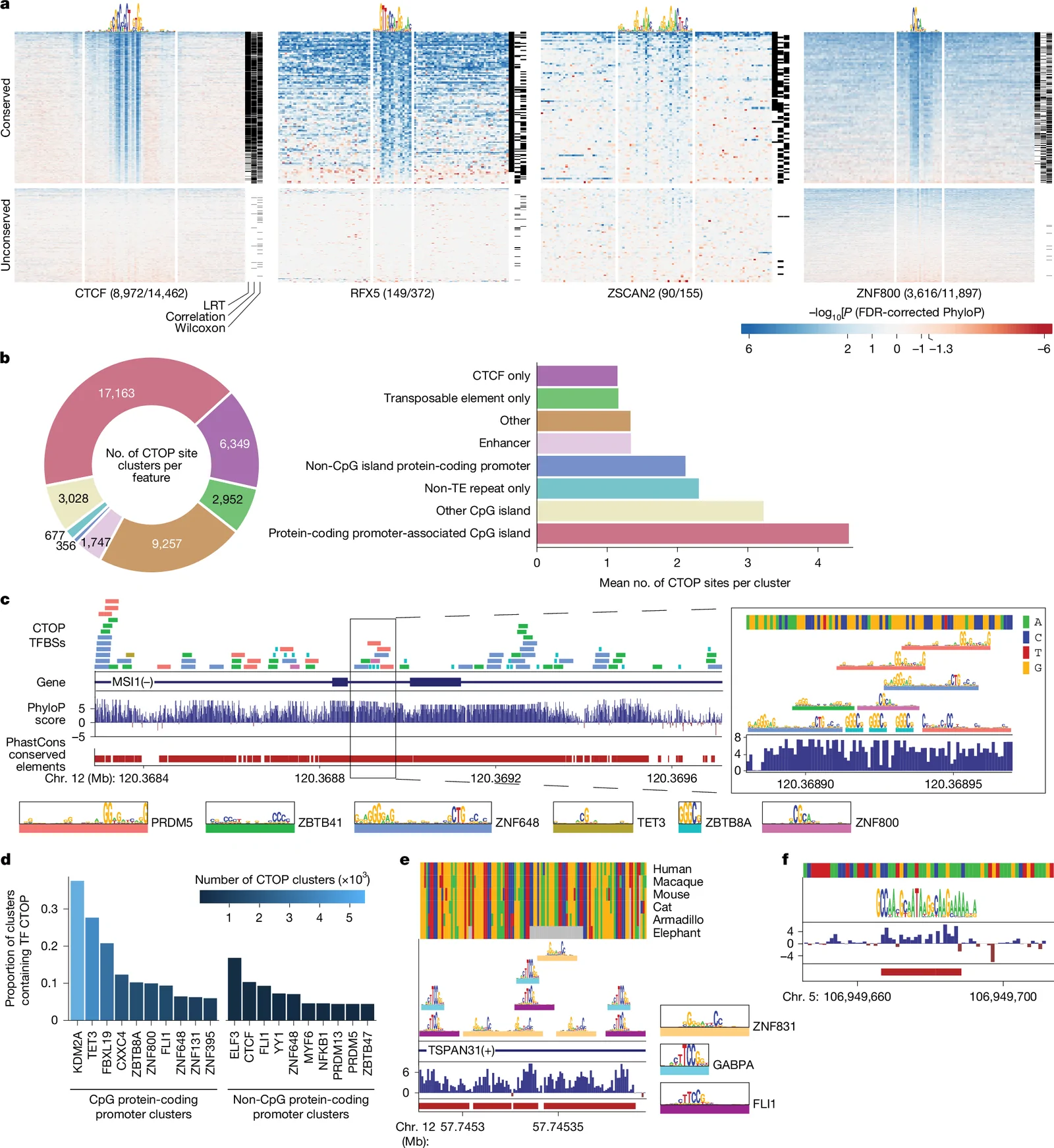
Conservation of Codebook TF-binding sites and association with genomic features. **a**, Heatmaps of FDR-corrected phyloP scores over the PWM match and 50 bp flanking for TOP sites for four TFs (two controls and two Codebook TFs). Statistical test results (see main text and Methods) are indicated on the right. **b,** Left, donut plot of the proportion and number of clusters of CTOP sites that overlap the indicated genomic features. Right, bar plot of the mean number of individual CTOPs contained in clusters that overlap the examined genomic regions. **c**, A 1,420-bp, CpG-island-overlapping CTOP cluster (chromosome 12: 120,368,293–120,369,713). Zoonomia 241-mammal phyloP scores and Multiz 471 Mammal alignment PhastCons conserved elements are shown. **d**, Bar plot of the frequency of TFs with CTOPs that occur most frequently in CTOP clusters that overlap CpG and non-CpG protein-coding promoters, respectively. **e**, CTOP cluster overlapping the non-CpG promoter at chromosome 12: 57,745,278–57,745,396. **f**, CTOP site for the KRAB-C2H2-zf protein ZNF689 overlapping an L1M4-type L1 element located at chromosome 5: 106,949,666–106,949,692. Source data

Together, the Codebook assays and PWMs pinpointed genomic loci that are bound directly by each TF in vivo (for example, in ChIP–seq) by identifying sites that are also bound in vitro (that is, GHT-SELEX) and that contain a PWM match, thus enabling base-level resolution. We refer to these as ‘triple optimized overlap’ (TOP) sites, which are taken as the overlap of the three datasets (ChIP–seq, GHT-SELEX and PWM matches) after applying optimized score thresholds for each (see Methods for details). To further gauge functionality of the TOP sites, we examined whether the pattern of per-nucleotide conservation^29^ at each site is consistent with the sequence preference of the TF driving local sequence constraint. To do so, we tested whether the motif matches at TOPs are more highly conserved than a neighbouring sequence. We also tested whether the degree of conservation at each position in the motif match correlates with the contribution of that base to predicted relative affinity (Methods). Figure 3a shows several examples that illustrate the apparent conservation of PWM matches for both control and Codebook TFs. Overall, 85 out of the 101 Codebook TFs (and 31 out of the 36 controls) with successful GHT-SELEX and ChIP–seq data displayed conservation of at least one TOP site (FDR < 0.1). In total we identified 113,577 such conserved TOP (CTOP) sites (82,760 for Codebook TFs and 30,817 for controls), which encompassed 1,394,530 bases in the human genome. These results provide strong support for the functional importance of Codebook TF-binding sites in the genome.

Many of the CTOP sites were either overlapping or adjacent to other CTOP sites for the same or different TFs, which suggests that these sites may be part of a larger regulatory element. We therefore grouped CTOPs that were within 100 bp from each other, which produced 41,529 CTOP clusters (of which 24,733 were singletons and 16,796 contained multiple CTOPs). The majority (35,583; 85.7%) overlapped with the UCSC PhastCons track. Thus, Codebook TFs binding to these regions represent a potential biochemical function for these previously identified conserved elements. The remaining 5,946 CTOPs seem to represent new functional annotations that were not readily detected by comparative genomics alone. It is known that TF binding motifs can provide additional functional information (for example, degenerate positions in motif matches) that can explain complex conservation patterns^30^.

Codebook TFs with the largest number of CTOP sites were typically associated with promoters (36.9% of all CTOP clusters) and/or CpG islands (47.3% of CTOP clusters) (Fig. 3b). The majority of CpG islands that overlapped with the promoters of protein-coding genes (60.4%; 8,111 out of 13,427) contained CTOP sites, with an average of 4.55 CTOP sites per CpG island. Moreover, 56 out of 101 of the Codebook TFs analysed had at least one CTOP site in a CpG island. A CTOP that overlaps a CpG island is shown in Fig. 3c. The extent of specific, conserved and intrinsic occupancy of CpG islands by many TFs of diverse classes was surprising; CpG islands are known to be enriched for binding sites for specific TFs (for example, SP1, NRF1, E2F and ETS families), which we did observe (Fig. 3c–e), but CpG islands typically have little deep conservation^31^. The CXXC proteins can specifically recognize unmethylated CG dinucleotides and modulate chromatin at promoters^32^, and we did observe that the CXXC proteins KDM2A, CXXC4, FBXL19 and TET3 predominantly bind CpG islands. However, the abundance of CG dinucleotides in CpG islands has been attributed primarily to their lack of methylation in the germline rather than to primary sequence constraint or binding to TFs^33^. The Codebook data therefore provide a new lens for the study of CpG islands. Notably, many of the Codebook TFs with CTOP sites in CpG islands recognize elaborate CG-rich motifs, often with degenerate positions and long gaps between invariant bases (Fig. 3c).

CTOP clusters were also found in non-CpG island protein-coding promoters (Fig. 3b): 678 out of 6,606 such promoters, defined as −1,000 to +500 relative to the transcription start site (TSS). These clusters were not dominated by any specific TFs, although some TFs were more prevalent than others (for example, ELF3 and CTCF in control TFs, and the Codebook TF ZNF648) (Fig. 3d). Figure 3e shows an example of a CTOP cluster in a non-CpG promoter, located early in the first intron of the *TSPAN31* gene, which exhibits apparent conserved spacing and orientation of multiple Codebook TF-binding sites. By contrast, CTOP clusters outside promoters and CpG islands often contained just one or two CTOP sites (Fig. 3b). One example is a strongly conserved ZNF689-binding site found in a lncRNA intron and overlapping an L1M4 transposon (Fig. 3f).

CTOP clusters also overlapped with known and putative enhancers: 1,825 overlapped with HEK293 enhancers (defined by ChromHMM^11^), and 4,057 were found in the extensive GeneHancer annotation set^34^. These lower numbers, relative to promoters, could be attributed to the relatively rapid evolution of enhancers^35^, a lack of complete knowledge of enhancer identities, a depletion of enhancer-related functions among the Codebook TFs or to other, non-enhancer functions of these conserved sites, such as control of heterochromatin or other aspects of chromosome architecture.

## Codebook motifs predict gene expression

**Extended Data Fig. 8:**
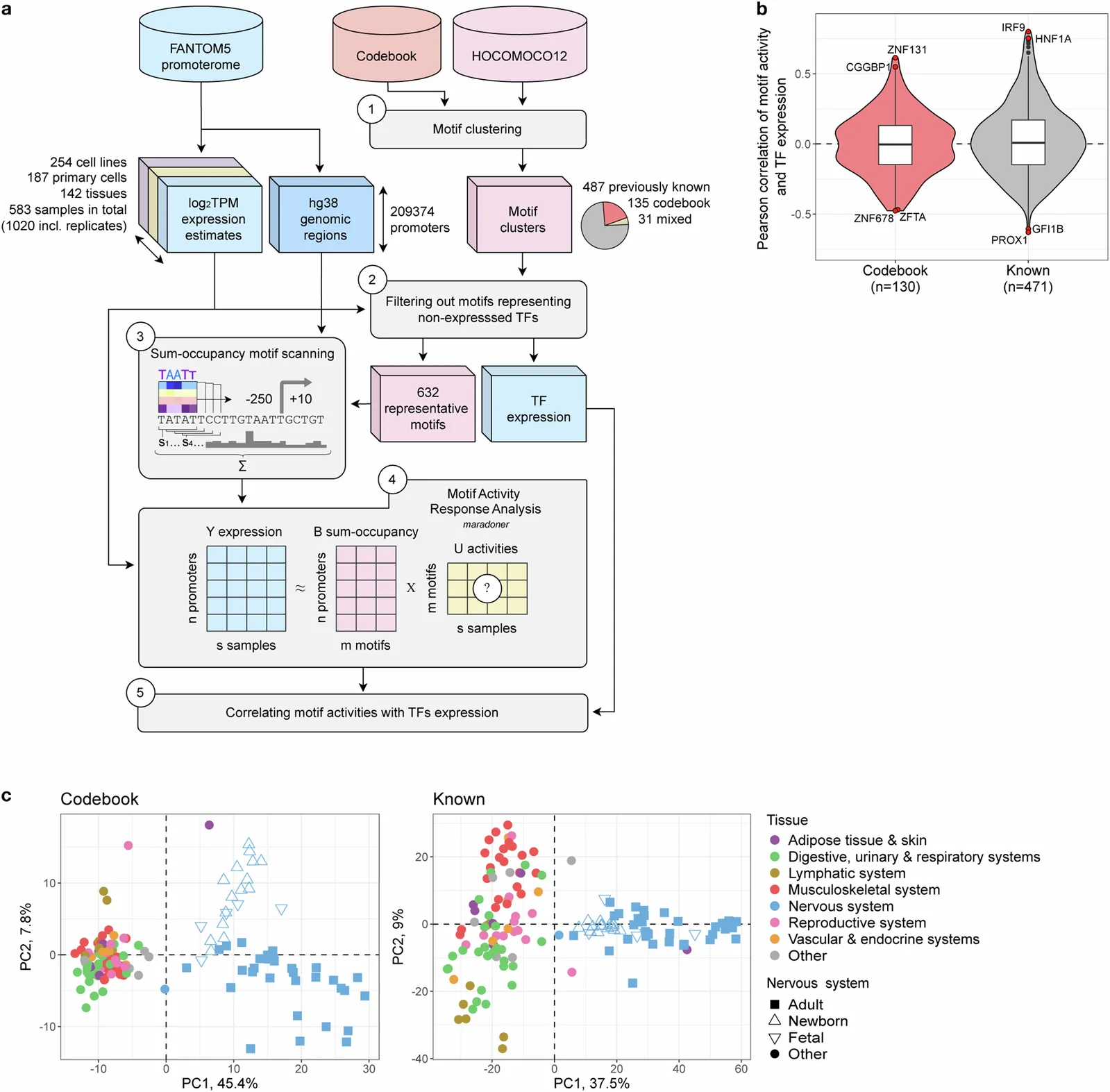
Motif activity response analysis highlights the relevance of Codebook motifs. **a**, MARA was conducted in five consecutive steps. (1) TF binding motifs from Codebook and HOCOMOCO^29^ (v12) were clustered, yielding 653 clusters, 135 of which consisted solely of Codebook motifs. (2) Motif clusters were filtered based on the total log_2_TPM of member TFs, resulting in 632 clusters (incl. 130 Codebook-only clusters) of expressed TFs’ motifs. (3) Representative motifs for each cluster were used to scan 209,374 genomic regions of 260 bp (250 bp upstream and 10 bp downstream relative to the representative TSS position of each promoter). (4) The sum-occupancy scores were used to perform the motif activity response analysis with the MARADONER Python package; (5) Pearson correlation coefficients were calculated for each representative motif by comparing the total TF expression against motif activities from MARA. **b**, Correlation between the TF expression (log_2_ TPM) and the activity of Codebook-specific (left) and known (right) motifs, the top 2 activators and inhibitors are labeled. Center line: median; box limits: upper and lower quartiles; whiskers: minimum and maximum values within the 1.5x interquartile range; dots: values outside the 1.5x interquartile range. The number of motif clusters (n) is 130 for Codebook and 471 for the known motifs. **c**, Principal component analysis of FANTOM5 data for 142 human tissues using motif activities of 130 Codebook (left) and 471 known (right) representative motifs. X- and Y-axes: the first two principal components. Marker color: tissue groups, marker shape: nervous tissue development stage. TF: transcription factor, TPM: CAGE tags per million, CAGE: cap analysis of gene expression, TSS: transcription start site. Source data

Conservation of TF-binding sites is a strong indicator that they are functional elements, but it does not inherently reveal in what conditions they are active and what processes they regulate. To link Codebook motifs to biological functions, we used motif activity response analysis (MARA)^36^, a framework that models relationships between promoter motif occurrences and gene expression in different biological samples. For MARA, we used 632 motif clusters representing expressed TFs (Supplementary Table 11), scanned the FANTOM5 promoters^37^ with a representative motif of each cluster and used the resulting scores to predict promoter activity (normalized CAGE tag counts) in 583 biological samples (tissues, primary cells and cell lines) using matrix-variate Bayesian regression (Methods, Extended Data Fig. 8a and Supplementary Table 12). Next, for each motif cluster, we calculated the Pearson’s correlation coefficient (PCC, *r*) between the inferred motif activity (the motif-specific regression coefficient) and the expression of the corresponding TFs, across all samples. The distribution of *r* values across 130 motif clusters containing only Codebook motifs was similar to that of clusters containing previously known motifs (Extended Data Fig. 8b). This result supports the notion that the Codebook proteins are bona fide regulatory factors.

**Fig. 4:**
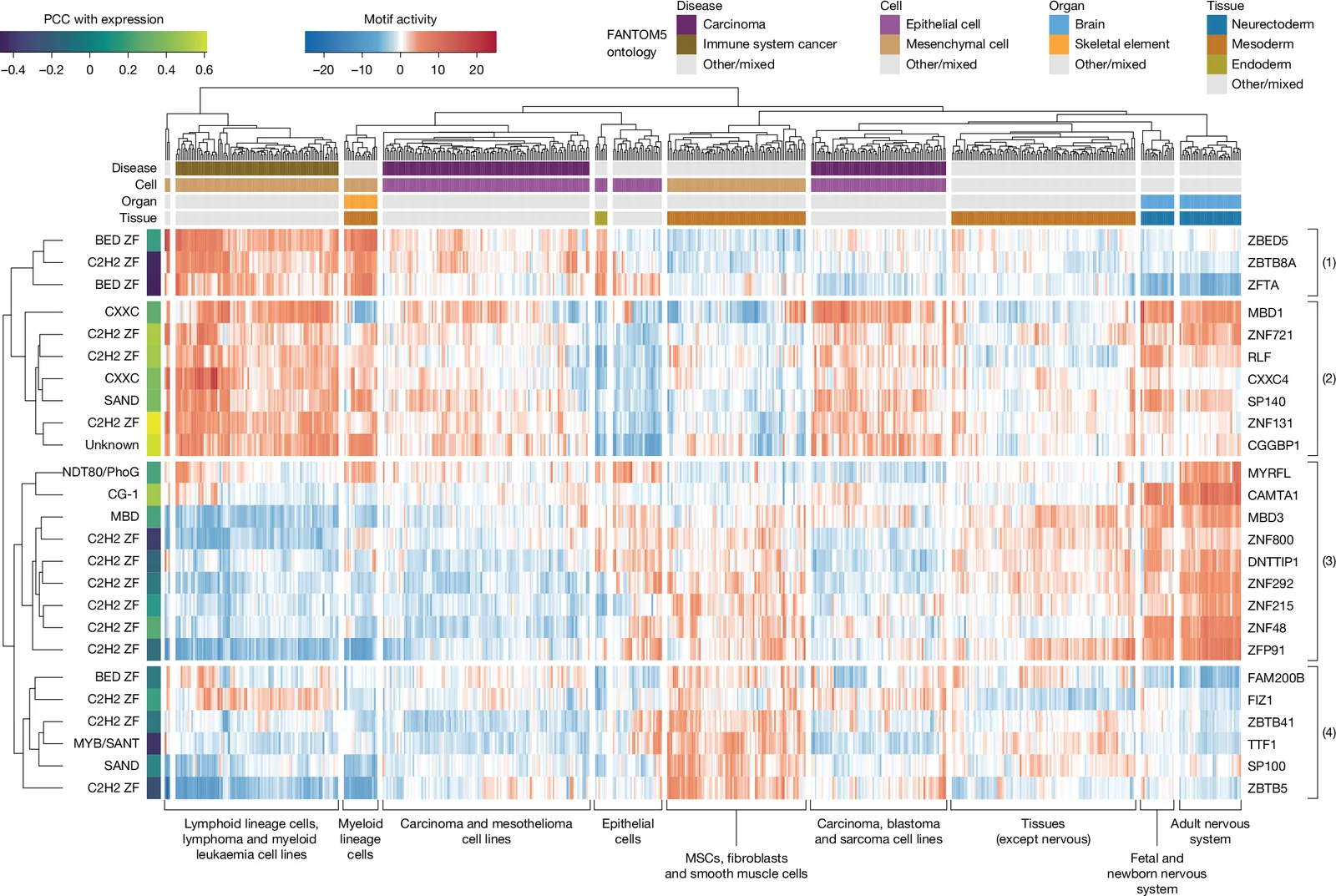
MARA for Codebook motifs. Heatmap of biclustered MARA-inferred motif activities. The *x* axis depicts 583 FANTOM5 samples, and clusters were manually labelled according to the predominant cell and tissue types. The *y* axis depicts TFs representing 25 Codebook motif clusters with the most significant differential activity across FANTOM5 samples. Additional columns (left to right) show DBDs of the representative motif and the correlation between TF expression and motif activity. Extra rows (top to bottom) depict FANTOM5 ontology-based sample classification using the selected groups from disease state (DOID: 4), cell type (CL: 0000003), organ (UBERON: 0000062) and tissue (UBERON: 0000479) terms, with clusters coloured by the predominant term if assigned to at least 40% of the samples. Source data

To explore cellular functions that the Codebook TFs might regulate, we examined the 25 Codebook-only motif clusters with the highest variance in activity across the FANTOM5 samples. Biclustering activities produced four major TF groups (Fig. 4): (1) TFs targeting genes that are preferentially expressed in immune cells, including the transcriptional repressors ZBTB8A and ZFTA, the targets of which are specifically repressed in the brain, and are often activated in ependymomas caused by ZFTA fusion with RELA^38^; (2) TFs active in both immune cells and cancer, including proteins that bind unmethylated CG-containing motifs (CGGBP1, CXXC4 and SP140), the transcriptional activator ZNF131, and MBD1; (3) TFs with target genes specifically expressed in nervous tissues, including neuron maturation regulators CAMTA1 and ZNF292, neurogenesis-associated repressors MBD3 and DNTTIP1, as well as MYRFL, the paralogue of myelin regulatory factor (MYRF, critical for central nervous system myelination), and exclusively active in the adult nervous tissues; (4) TFs mostly restricted to mesodermal primary cells, including the rRNA transcription regulator TTF1 and the zinc finger and BTB domain-containing proteins ZBTB41 and ZBTB5, which were found to stimulate cell proliferation^39,40^.

We obtained similar outcomes for the global patterns of activity among all 130 Codebook-only motif clusters and FANTOM5 samples using principal component analysis: the first component mainly separated nervous system from all other samples, whereas the second component separated the developing nervous systems from adult (Extended Data Fig. 8c). Taken together, the motif activities of Codebook TFs distinguish FANTOM5 tissues and cell types and highlight many candidates for new transcriptional regulators of genes involved in the inflammatory response, cell cycle control and nervous system development.

## Codebook TFs and the dark matter genome

TF-binding sites in promoters and enhancers are easily rationalized as likely to be being involved in the regulation of adjacent genes. A principal result of the Codebook project is that dozens of Codebook TFs instead bind preferentially to other regions of the genome. An accompanying manuscript^11^ focuses on the finding that roughly half of the examined TFs bind predominantly outside promoters and enhancers in ChIP–seq, with most TFs having a distinct set of binding sites that are supported by both GHT-SELEX and motif matches. Among these TFs are KRAB-containing C2H2 zinc fingers (KZNFs), which are best known for binding to specific classes of endogenous retroelements and nucleate regions of H3K9 trimethylation^41^. Until now, it has not been proven that the precise binding of KZNFs to specific retroelement subtypes is defined entirely by the sequence specificity of the KZNFs alone^42^. However, the Codebook GHT-SELEX data abundantly demonstrate that this is indeed the case^10^.

**Extended Data Fig. 9:**
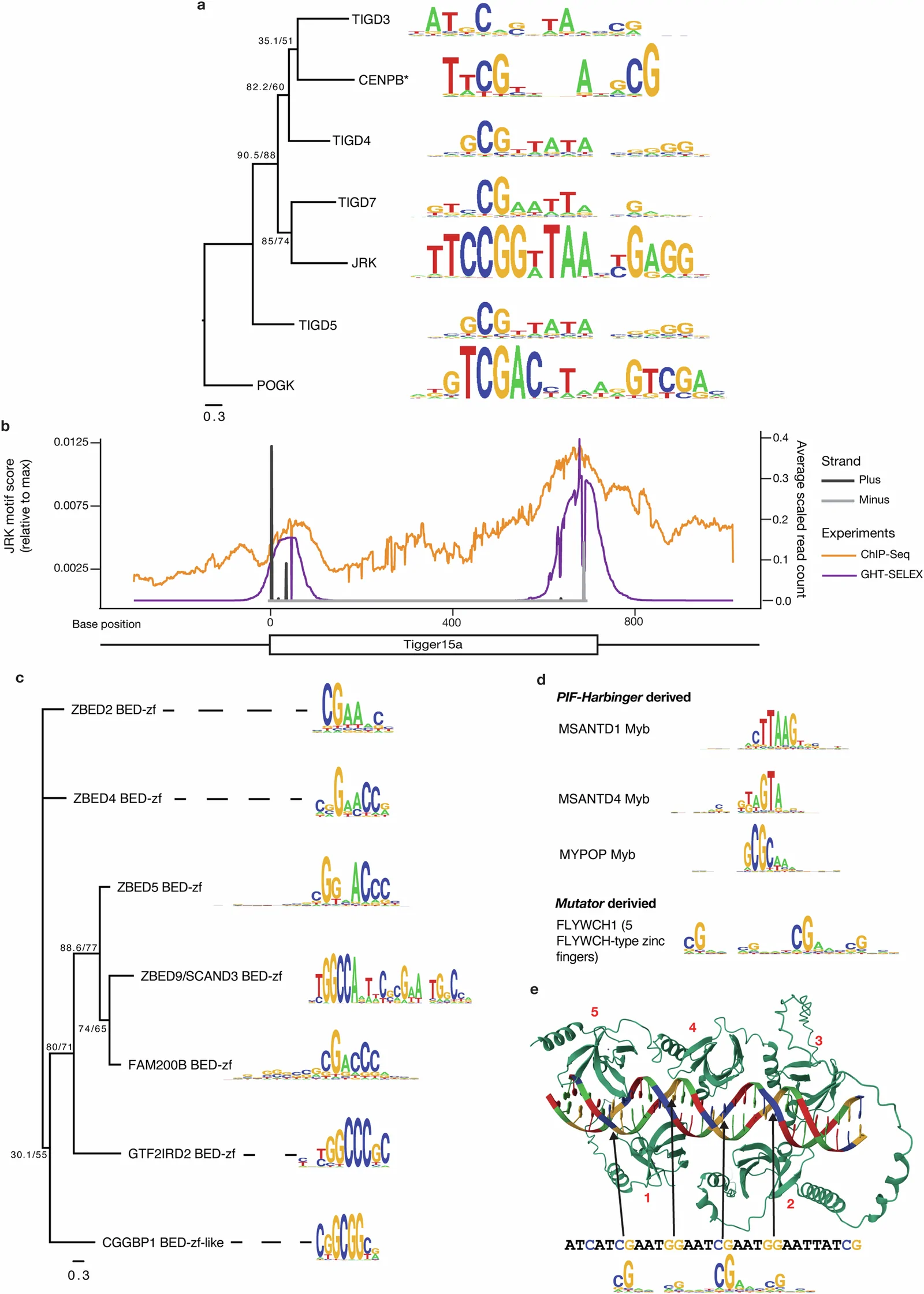
Transcription factors co-opted from DNA transposons. **a**, Motif logos of human TFs that are derived from the domestication of *Tigger* and *Pogo* DNA transposons and have previously known DNA binding motifs. Phylogenetic tree was derived from DBD sequence alignments produced with MAFFT L-INS-I^82^, and produced using the same method described in the Extended Data Fig. 2e legend. The tree is rooted on POGK, which is derived from a more distantly related family of *Pogo/Tigger*-like elements relative to the other proteins^83,84^. Sequence logos are Codebook-derived, except for CENPB, from the liturature^85^. **b***,* average per-base read count over Tigger15a TOPs in the human genome, for JRK ChIP-seq (orange) and GHT-SELEX (purple), with peak sequences aligned to the Tigger15a consensus sequence. JRK PWM scores at each base of the Tigger15a consensus sequence are shown in black (plus strand) and grey (minus strand). **c**, Sequence logos of Codebook human TFs that are derived from the domestication of DBDs from *hAT* superfamily DNA transposons. Phylogenetic tree was derived from DBD sequence alignments produced with MAFFT L-INS-I^82^, although for ZBED4 the third of its four BED-zf domains was used. The tree was produced using the same method as described in the Extended Data Fig. 2e legend. **d**, Sequence logos of TFs derived from domestication of *Mutator* and *PIF-Harbinger* DNA transposons. **e**, Alphafold3 structure of the five FLYWCH-type zinc-fingers of FLYWCH1, and a TOP sequence from chr10:42167256-42167276 with 5 bp flanks. Zinc finger domains are numbered according to their order in FLYWCH1 from the N-terminus to the C-terminus. Long linkers and terminal extensions are hidden for simplicity. DNA-protein structures were predicted with AlphaFold3 (available under Creative Commons licence CC-BY-4.0 and visualized using Mol*^81^. DBD: DNA-binding domain, PWM: position weight matrix.

The Codebook data also underscore the fact that transposons contribute to the TF repertoire itself^43^. Sixteen of the Codebook TFs (and two controls) that successfully produced motifs possess a DBD that has itself been derived from a DNA transposase. These include CGGBP1, six proteins containing BED-zf domains, six with the related CENBP or Brinker domains, three with transposon-derived MYB/SANT/MADF domains and FLYWCH1 (Supplementary Note 2). The PWMs obtained for CENPB or Brinker TFs were often long and information-rich, consistent with their presumed ancestral role in binding specifically to transposons in a large genome (Extended Data Fig. 9a and Supplementary Note 2). A notable example is JRK, a TF that is found broadly in mammals^44^ and derived from an ancient domesticated Tigger DNA transposon^45^. All DNA transposons, including Tigger, have been transpositionally inactive in the human lineage for over 40 million years and are presumably ‘fossils’. Genomic binding of JRK is enriched for the same family of Tigger elements from which it seems to be derived (Supplementary Note 2). The consensus sequence for this Tigger element family contained PWM-predicted binding sites for JRK in the terminal repeats (Extended Data Fig. 9b), a finding consistent with its presumed ancestral role in transposition. To our knowledge, it is only the second human protein known to retain this quality (the other is SETMAR^46^). These cases represent the simultaneous introduction of a multitude of potential *cis*-regulatory elements, and a TF that binds them, by the same transposon.

## Completing the human TF codebook

**Fig. 5:**
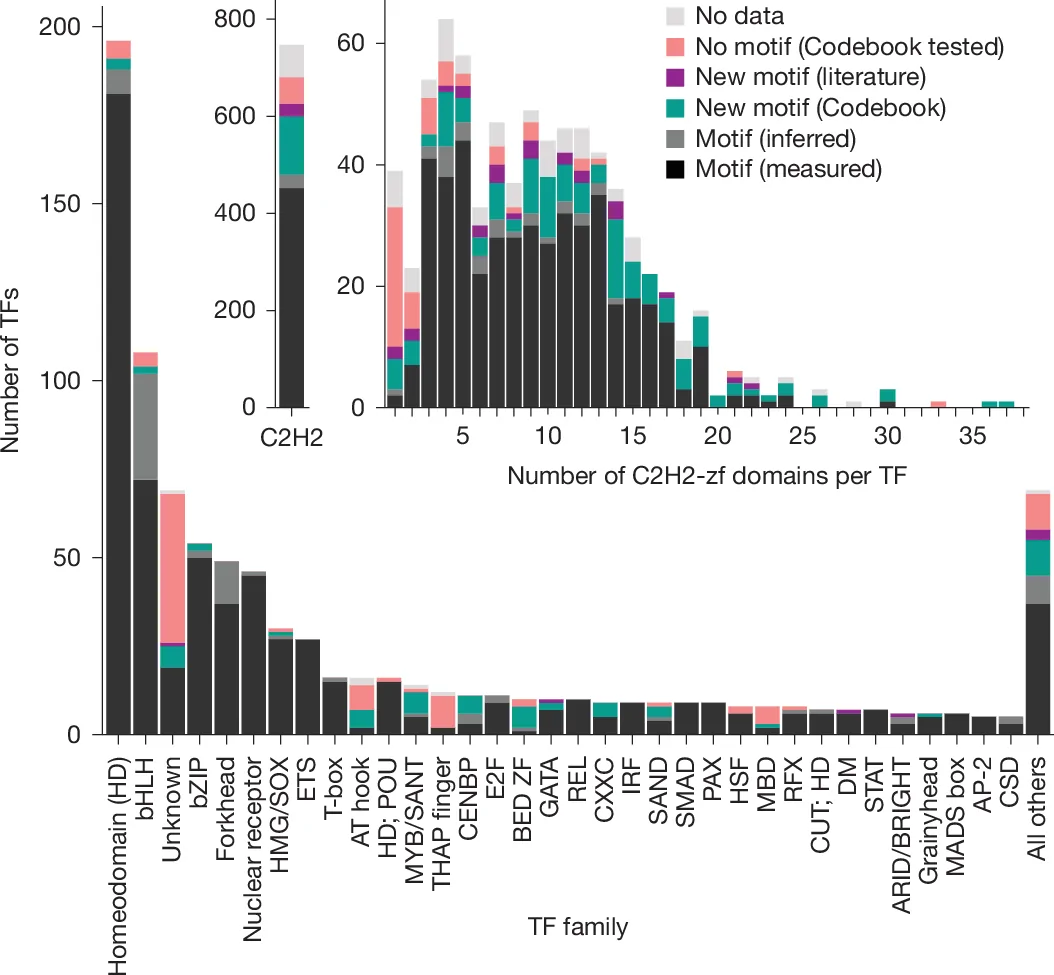
Motif coverage of human TFs by DBD family. Motif coverage across human TFs categorized into structural classes from ref. ^1^. Motifs that were inferred or measured prior to the Codebook project^1^ are distinguished from newly measured motifs. C2H2-zf proteins are shown separately and subdivided by the number of C2H2-zf domains (inset) because of their large numbers, and to illustrate trends. See Supplementary Table 14 for underlying information. Source data

**Extended Data Fig. 10:**
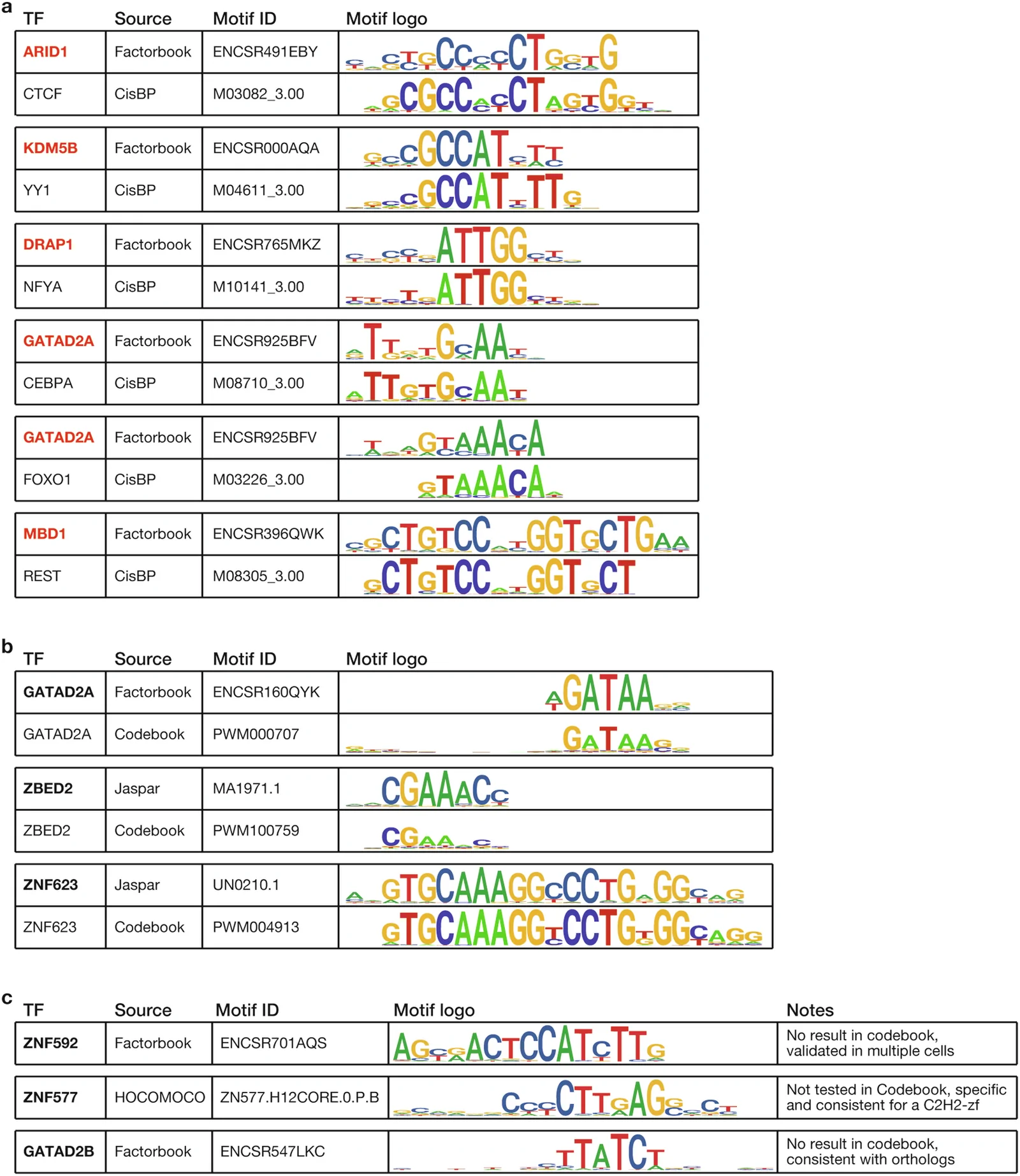
Examples of evaluation of external PWMs. **a**, Cases in which the external PWM matches the PWM of a well-studied TF that is a frequent “contaminant” motif in ChIP-seq^86^. In each example, the top sequence logo represents the external PWM, and the bottom sequence logo represents a highly similar CisBP PWM for a “contaminant” motif. **b**, Cases in which the external PWM (top in each example) is consistent with the Codebook PWM for the same TF (bottom in each example). **c**, External PWM sequence logos that cannot be explained as known contaminants or artifacts, some of which are supported by multiple lines of evidence and thus appear accurate. PWM: position weight matrix^1,7,8,12^. Sequence logos were generated from PWMs obtained from JASPAR^64^, Factorbook^65^, CisBP^53^ and HOCOMOCO^29^. Jaspar PWMs are available under CC BY-NC 4.0 license.

The primary purpose of Codebook was to assess 332 uncharacterized and putative human TFs for sequence-specific DNA-binding activity and to produce trustworthy motifs for them to assemble a complete human TF motif collection. This goal was mostly achieved. Codebook was limited to five assays, with ChIP–seq conducted in only a single cell line, yet slightly more than half of the tested TFs succeeded. Moreover, there is reason to think that many of the unsuccessful putative TFs are probably not DNA-binding proteins or bind DNA with little or no sequence specificity (Supplementary Note 3). As noted above, a subset of the Codebook TFs, as well as other poorly characterized TFs, have been analysed by others since our study began. To evaluate the current scope of known human TF specificities, we surveyed other databases for PWMs for putative TFs that were not included in this study or were not found among the 177 Codebook successes (note that 20 Codebook TFs have recently generated PWMs and external ChIP–seq data, with a comparable performance to Codebook PWMs; Extended Data Fig. 4f–h). A conservative manual curation (see Extended Data Fig. 10 for examples of PWMs that passed curation) identified 33 additional human TFs (that is, beyond the 177 that were successful in Codebook) that have at least one plausible motif available in datasets that have been released since our 2018 TF census^1^ (Supplementary Table 13), which leads to a new total of 1,421 human TFs with characterized sequence specificities (Fig. 5 and Supplementary Table 14). Supplementary Data 1 contains representative PWMs for all 1,421 TFs (see Supplementary Table 10 for motif details).

This enumeration leaves 175 proteins, mostly with conventional DBDs, for which there is still no known DNA-binding motif. Not all proteins with such domains are necessarily TFs, as noted above, and our re-curation of TFs that were unsuccessful in our assays suggests that 83 of these—nearly half—seem unlikely to be bona fide TFs (Supplementary Note 3 and Supplementary Table 14). At the same time, new DBD classes continue to be identified (for example, WH^47,48^). Furthermore, some TFs may bind only to methylated DNA, a possibility we explore in a separate manuscript in this collection^13^. Ongoing advances in the prediction of protein and protein–DNA structures have the potential to identify additional candidates for sequence-specific DNA binding. Thus, although completion of the objective to obtain a motif for every human TF now seems to be much closer, the list of probable human TFs continues to evolve.

An important technical demonstration of the Codebook project is that the simultaneous application of multiple experimental strategies and multiple motif-derivation and motif-scoring strategies was highly beneficial, with no method either dominating or dispensable^12^, a result consistent with previous benchmarking efforts^49,50^. Consequently, many of the Codebook TFs are now among the best-characterized human DNA-binding proteins in terms of their sequence specificity. In particular, obtaining in vivo and in vitro genomic binding profiles, together with assessing binding to randomly generated artificial sequences, is advantageous in that it facilitates disentanglement of direct and indirect binding, the contribution of the cellular environment and the highly non-random nature of the genome sequence. Only for a small handful of the 1,000+ previously characterized TFs is there such a rich combination of data types. A much better perspective on human gene regulation, genome function and evolution may be obtained from the generation of such data for all human TFs.

## Methods

### Plasmids and inserts

Sequences and accompanying information are given in Supplementary Tables 2–4. In brief, we selected Codebook TFs (and their DBDs) from information published in a previous study^1^ and posted at https://humantfs.ccbr.utoronto.ca. Inserts named with a ‘-FL’ suffix correspond to the full-length ORF of a representative isoform of the protein. Those with a ‘-DBD’ suffix contain all of the predicted DBDs in the protein flanked by either 50 amino acids or up to the amino or carboxy terminus of the protein. Those with a ‘-DBD1’, ‘-DBD2’ or ‘-DBD3’ suffix contain a subset of the DBDs present in the proteins; these were manually designed, mainly for large C2H2-zf arrays. Inserts were obtained as recoded synthetic ORFs (BioBasic) flanked by AscI and SbfI sites and subcloned into up to three plasmids: (1) pTH13195, a tetracycline-inducible, N-terminal eGFP-tagged expression vector with FLiP-in recombinase sites^8^; (2) pTH6838, a T7-promoter driven, N-terminal GST-tagged bacterial expression vector^53^; and (3) pTH16500 (pF3A-ResEnz-egfp), a SP6-promoter driven, N-terminal eGFP-tagged bacterial expression vector, modified from pF3A–eGFP^7^ to contain the two restriction sites after eGFP.

### Protein production

Each experiment used a protein expressed from one of the following systems: (1) FLiP-in HEK293 cells (Thermo Fisher Scientific, R78007), induced with doxycycline for 24 h, used for inserts in pTH13195; (2) PURExpress T7 recombinant IVT system (NEB, E6800L), for inserts in pTH6838; or (3) SP6-driven wheat germ extract-based IVT (Promega, L3260), for inserts in pTH16500.

### DNA-binding assays

We followed previously described protocols for ChIP–seq^8^, PBMs^50^ and SMiLE-seq^7^. Detailed descriptions of GHT-SELEX, HT-SELEX, ChIP–seq and SMiLE-seq data collection and initial analyses are provided in the accompanying papers^10–13^. For PBMs, we analysed proteins on two different universal PBM arrays (HK and ME), with differing probe sequences^54^, but did not analyse most C2H2-zfs owing to low success rates with this assay, presumably due to long binding sites. By default, we analysed each protein twice by ChIP–seq (with full-length constructs only)^11^. We analysed each construct by HT-SELEX and GHT-SELEX once by default (that is, as full-length and DBDs), and some constructs were analysed multiple times to examine the impact of experimental variables as the GHT-SELEX method was developed^10^. We ran SMiLE-seq assays for 278 TFs, which corresponded to 388 constructs. Controls were omitted given that most were selected because they already had published SMiLE-seq data, which resulted in 299 TFs with SMiLE-seq-derived motifs. A subset of Codebook proteins (mainly those with unknown DBDs) were also omitted owing to lack of success in all other assays. A randomly selected subset of 82 constructs was analysed multiple times to assess reproducibility across SMiLE-seq experiments. Anti-eGFP antibody (Ab290, Abcam) was used as the major antibody across all the assays (ChIP–seq, GHT-SELEX and western blots), with method-specific amounts^10,11^. Each ChIP reaction used 2 µl undiluted polyclonal antiserum, corresponding to 10 µg total IgG, immobilized on 60 µl protein G magnetic bead suspension (Dynabead 10004D, ThermoFisher). For HT-SELEX and GHT-SELEX, antibody-bead master mixes were prepared by immobilizing 6 µl antiserum on 100 µl protein G sepharose bead slurry (Cytiva, 28-9670-70), and 1 µl aliquots of the resulting mixture were used for each selection, which corresponded to approximately 0.06 µl antiserum (300 ng total IgG) per reaction. For western blotting, membranes were incubated in 15 ml antibody solution diluted 1:5,000, which corresponded to approximately 15 µg total IgG per membrane.

### Data processing and motif derivation

The accompanying paper^12^ describes motif derivation and evaluation in detail. In brief, after initial preprocessing, we obtained a set of ‘true positive’ (likely to be bound) sequences for each individual experiment. A total of 721 out of 4,873 experiments were removed at this step owing to a low number of likely bound sequences or to other technical issues, as documented in Supplementary Table 4. We then applied a suite of tools (listed in Supplementary Table 15) to a training subset of the data from each experiment and tested the resulting motifs on a test subset of the data from the same experiment and on the independent data for the same TF (that is, the test sets from all other experiments performed for the same TF). We used a binary classification regimen for all experiments and all motifs and scored the motifs using a variety of criteria, including the area under the receiver operating characteristic curve (AUROC) and the area under the precision–recall curve. The full set of motifs (as PWMs) is available at Zenodo^55^, and an interactive browser is available at https://mex.autosome.org.

### Systematic filtering of artefactual motifs

While curating the datasets and assembling a reliable motif collection, we accounted for enrichment of similar artefact motifs. These recurrent DNA patterns were detected owing to systematic experimental noise or peculiarities of particular motif discovery tools. For example, in HT-SELEX experiments, an ACGACG motif was often enriched. This sequence is a presumed artefact as it matches the constant flanking region. In experiments with cell lysates, motifs of abundant native proteins in HEK293 cells were sometimes enriched (for example, NFI, YY1 and ETS-family TFs). To minimize the influence of these artefacts, we performed the following tasks: (1) manually compiled a list of recurrent artefact motifs (Supplementary Table 16); and (2) scanned the entire motif collection with MACRO-APE^56^ and removed motifs that were highly similar to those in our catalogue of artefacts. We also removed motifs that matched the constant, non-variable regions of the DNA used in HT-SELEX, GHT-SELEX and SMiLE-seq experiments. We did not filter out ETS-related motifs for ETS-family positive controls, such as ELF3, FLI1 and GABPA. Subsequent to expert curation, we confirmed that enriched *k*-mers in HT-SELEX experiments did not correspond to potential artefacts associated with individual expression systems^10^.

### Evaluation of motif discovery success rate

To estimate the success rate of different motif discovery tools across different platforms, we started with the successful experiments and TFs. For each combination of a platform X (for example, ChIP–seq) and a motif discovery tool Y (for example, Autoseed), we computed the number of experiments (Extended Data Fig. 1c) or TFs (Extended Data Fig. 1d) that produced a motif highly similar to the reference motif (that is, the one manually curated for the TF). For a specific experiment or TF, we took the entire set of candidate motifs generated by that combination of platform X and tool Y and checked whether any of those motifs passed the similarity threshold using a one-pass scan of MACRO-APE (v.3.06)^56^ ScanCollection with the following parameters: -c 0.05 --rough-discretization 10 --precise 100500. Before comparison, the motifs were converted to log-odds PWMs as previously described^12^.

### Experiment evaluation by expert curation

To gauge the success of individual experiments, we implemented an ‘expert curation’ workflow with an initial voting scheme in which a committee of annotators gauged whether individual experiments should be deemed successful (that is, included in subsequent analyses). All experiments were examined by at least three annotators. A subcommittee (A.J., I.V.K. and T.R.H.) jointly resolved all cases of disagreement among initial annotators (around 300 experiments) and then reviewed all successful experiments. Annotators had access to an early version of the MEX portal (https://mex.autosome.org, as previously described^12^), which contains the results of all PWMs scored against all experiments, and they were tasked with gauging whether the experiments produced PWMs that were similar across experiments or scored highly across experiments. Annotators also considered whether the motif was consistent with those for other members of their protein family (for example, BHLHA9 produced an E-box-like motif, CAnCTG) and/or similar between closely related paralogues (for example, ZXDA, ZXDB and ZXDC all produced similar motifs). We also considered whether (and how many) ‘peaks’ were obtained from ChIP–seq or GHT-SELEX, and whether these peaks were common to independent experiments (for example, both ChIP–seq and GHT-SELEX). Annotators were further given a measure of similarity between Codebook PWMs and any PWMs in the public domain, as well as enrichment of known or suspected common contaminant motifs in any experiment.

### Post-evaluation peak processing

After identification of successful experiments, we re-derived peak sets for ChIP–seq and GHT-SELEX experiments to obtain a single peak set for each TF, as described in the accompanying papers^10,11^. In brief, for ChIP–seq, we repeated peak calling using MACS2 (v.2.2.9.1)^57^ and experiment-specific background sets using a previously described method^8^, then merged the peak sets for replicates of the same TF with BEDTools (v.2.30.0) merge^58^ (see accompanying manuscript^11^: ‘ChIP peak replicate analysis and merging’). We derived GHT-SELEX peaks using a new method, MAGIX, that calculates enrichment of reads in each cycle and treats different experiments as independent statistical samples to obtain a single enrichment coefficient per peak^10^.

### Expert motif curation

For this study, to identify a single representative PWM for each TF, we first compiled a set of the highest-scoring candidate PWMs for each TF (as summarized above and in a previous study^12^), then ran additional tests with them, using the reprocessed peak data, and manually evaluated the outputs. We started with the union of 3 sets of 20 PWMs for each TF: the 20 PWMs with the highest AUROC (as calculated using a previously described method^12^) on any successful ChIP–seq experiment for the given TF, any successful GHT-SELEX experiment for the given TF and any successful HT-SELEX experiment for the given TF. These PWMs were selected regardless of the dataset from which they were derived. We then reassessed these PWMs against ChIP–seq and GHT-SELEX data with two parallel methodologies. First, we recalculated the AUROC for each of the candidate top PWMs on the merged, thresholded sets of ChIP-seq peaks (*P* < 10^−10^)^11^ using AffiMX (v.1)^25^ to score each peak. We generated negative sets using BEDTools (v.2.30.0) shuffle^58^ with the -noOverlapping option to create sets of random genomic regions with the same number of peaks and with the same peak-width distribution as the corresponding ChIP–seq peak sets. We used the same technique to calculate AUROC values for GHT-SELEX, with thresholded peak sets (using a ‘kneedle’^59^ specificity value of 30 in the sorted enrichment values^11^). In parallel, we calculated the Jaccard index to measure the overlap between PWM matches (identified using MOODS (v.1.9.4)^51^ with -p 0.001) versus ChIP–seq peaks and GHT-SELEX peaks as two separate measures. The overlap in each case was maximized by applying different thresholds on the peak sets and choosing the cutoff at which the Jaccard index was the highest^10^. We then applied expert curation (by a committee consisting of A.J., T.R.H., K.U.L., A.F., R.R., M.A. and I.Y.) to choose a single representative PWM with high performance on all compiled scores that, all else being equal, also reflected reasonable expectation from the DBD class (including recognition-code-predicted motifs, see accompanying manuscript^10^) and had sufficient IC.

### Motif similarity analysis and clustering

We took two different approaches to determine the number of distinct motifs represented by the Codebook TFs and the number of previously unknown motifs added to the human TF repertoire. In the first approach, we identified the similarity between 1,582 PWMs representing the Codebook TFs (177 PWMs, 177 TFs) and TFs with previously known specificities (1,405 PWMs, 1,211 TFs). The Codebook TF PWM set is the set of representative PWMs for the 177 Codebook proteins with identified motifs. For TFs with previously known specificities, the set of PWMs identified as the ‘best’ in a previous study^1^ was used, except for two TFs (MTF2 and PHF1) that did not have an assigned PWM. For these two TFs, the PWMs were retrieved from CisBP^53^. We used the correlation between pairwise affinities to 150,000 random sequences of length 100, as calculated using MoSBAT (v.1)^25^, as the metric for PWM similarity. We then clustered the 177 Codebook TFs on the basis of these PWM similarities with PCC and average linkage and identified the ideal number of clusters (129) by determining the optimal silhouette value^52^ using the silhouette function from the ‘cluster’ package (v.2.1.8.1) in R (v.4.3.2), which corresponded to splitting PWMs into clusters at a distance of 0.76. We then clustered the entire set of 1,582 motifs and used the same distance to split the PWMs into clusters. This process resulted in 613 clusters, 92 of which contained only Codebook TFs. The set of motifs used in the clustering and their cluster membership are provided in Supplementary Table 10.

Independently, and to obtain a non-redundant set of motifs for MARA analysis, we followed a previously described procedure^60^, but with the addition of Codebook motifs. First, we merged HOCOMOCO (v.12)^29^ motifs with those evaluated in Codebook, preferably selecting those built with ChIPMunk and ranking high in benchmarking^12^ to maintain consistency with HOCOMOCO (v.12), which was fully built with ChIPMunk. Having the joint motif collection, we estimated the motif similarities with MACRO-APE (v.3.0.6)^56^ at the motif *P* value cutoff of 0.0005 and a default matrix discretization parameter (-d) of 1 (increased to 10 for improved precision only for motif pairs with the Jaccard similarity over 0.01 at -d 1). Next, using the pairwise motif similarity matrix, we performed agglomerative clustering (‘average’ linkage) with sklearn (v.1.8.0). The number of clusters was taken to maximize the silhouette score. Clusters of low-quality motifs (HOCOMOCO ‘D’) were discarded. Finally, for each cluster, a single representative motif was taken according to the highest average similarity to all other motifs in the cluster. The non-redundant set of representative motifs is available for download from Zenodo^61^, and the motif clusters annotation is available in Supplementary Table 11.

### Motif degeneracy analysis

To explore whether low IC is an intrinsic feature of some binding motifs, we adjusted the IC of PWMs and tested the prediction accuracy of ChIP–seq and GHT-SELEX binding sites. PWM IC was adjusted on a per-base-pair basis by iteratively scaling probabilities for each base at each position until the PWM reached an average IC of 1 bit per base pair. The script ‘logo_rescale.pl’ is available at GitLab (https://gitlab.sib.swiss/EPD/pwmscan).

### Comparison to external peak sets and PWMs

For comparison, we downloaded ChIP–seq peak sets from GTRD^62^ and ENCODE (4.12.2023)^63^ for all Codebook TFs. We then divided these data into four categories corresponding to the cell type: HEK293/HEK293T, HepG2, K562 and other cells. We preferentially selected the peak sets from GTRD because GTRD has processed the majority of ENCODE consortium experiments, together with many non-ENCODE experiments. When multiple experiments were available for a TF in a cell-type category, we selected the experiment with higher peak counts. If multiple computational methods had been used to derive peak sets for the selected experiment, we chose the peak set using a preferential order of MACS, GEM, SISSRS, PICS and PEAKZILLA. See Supplementary Table 7 for identifiers and metadata of the reference datasets.

For PWM scoring, the external peak sets were used as downloaded, with the exception of peak sets that were generated with the GEM peak caller, which have a peak width of 1 (summit only), and were therefore expanded 250 bases in both directions. For Codebook data, we used the merged and thresholded Codebook ChIP–seq peak sets as in ‘Expert motif curation’. We generated negative peak sets using BEDTools (v.2.30.0) shuffle^58^ with the -noOverlapping option to create sets of random genomic regions with the same number of peaks and the same peak width distribution as the corresponding ChIP–seq peak sets. We downloaded PWMs for all Codebook TFs from JASPAR (2024 version)^64^, HOCOMOCO (v.12)^29^ and Factorbook^65^ (downloaded 15 December 2023) (Supplementary Table 13). We scanned Codebook and external peak sets (and corresponding negative sets) with the representative (that is, expert-curated) Codebook motifs (PWMs) using AffiMX (v.1)^25^ and calculated AUROC values. Furthermore, for the 20 Codebook TFs with a successful Codebook ChIP–seq experiment, a Codebook PWM, an external ChIP–seq experiment and an external PWM, we compared the performance of PWMs across the different peak sets as follows. We first selected a single external PWM for each of the 20 TFs by scanning each PWM for a given TF on each external peak set for the same TF and identifying the PWM that produced the highest AUROC. We then used these highest scoring PWMs to scan the corresponding Codebook data and to calculate AUROC values.

### Curation of external motifs for previously uncharacterized TFs

For this analysis, we considered all proteins that were previously annotated as putative TFs^1^ but did not, at that time, have a credible motif. We downloaded all motifs for these TFs from JASPAR (2024 version)^64^, HOCOMOCO (v.12)^29^ and Factorbook^65^ (downloaded 15 December 2023), which resulted in a set of 484 PWMs that we then manually assessed with the following considerations: (1) whether similar motifs were obtained for the protein from multiple independent datasets; (2) whether the motif was consistent with the structural class of the TF and; (3) whether the motif was likely to describe the inherent specificity of the protein and not, for example, reflect a target site of another TF or a probable artefact. The curated motifs are listed in Supplementary Table 13 with more details in Supplementary Table 10. The PWMs themselves are available in Supplementary Data 1, and examples of artefactual and correct external motifs are shown in Extended Data Fig. 10.

### Identifying and scoring promoter sequences for MARA

MARA requires promoter activity (gene expression) data across samples and motif scores across promoters. The former was downloaded from the FANTOM5 web resource (https://fantom.gsc.riken.jp/5/), hg38_fair+new_CAGE_peaks_phase1and2_tpm_ann.osc.txt, log_2_-transformed with a pseudocount of 0.05 and filtered, leaving only promoters of genes encoded in the nuclear genome and removing time courses, perturbations and human total RNA samples. This process resulted in 209,374 individual promoters and 1,020 samples (including replicates) that belonged to 583 unique samples (142 tissues, 187 primary cells and 254 cell lines; Supplementary Table 12 and Zenodo^61^). Motif scanning was performed on regions from FANTOM5 hg38_fair+new_CAGE_peaks_phase1and2.bed, taking 250 bp upstream and 10 bp downstream from the representative TSS position as indicated in FANTOM5 data. Next, we used SPRY-SARUS (v.2.2.3; https://github.com/autosome-ru/sarus) to compute the sum-occupancy scores^66^ for each representative motif of the motif clusters (see the section ‘Motif similarity analysis and clustering’). The final analysis was performed with 632 motif clusters (130 clusters containing only Codebook motifs, 471 clusters of known motifs and 31 mixed clusters) corresponding to TFs that were jointly expressed >0 in at least one of the FANTOM5 samples.

For MARA, we used MARADONER (v.0.13)^67^, a command-line tool written in Python and available in the PyPi repository. The analysis was performed using maradoner create, maradoner fit and maradoner export with default parameters (https://github.com/autosome-ru/MARADONER).

Conceptually, MARADONER extends the original logic of MARA^36^ and isMARA^68^. The basic assumption is that the promoter activity in each sample is a linear function of sample-specific motif activities, for which each motif represents a set of TFs with shared binding specificity. We used the following matrix-variate linear mixed model:

$$Y={{{\boldsymbol{\mu }}}_{{p}}{{\bf{1}}}_{{s}}}^{{\rm{T}}}+{{\bf{1}}}_{{p}}{{\boldsymbol{\mu }}}_{{s}}+B\,U+E,\,E \sim {\rm{M}}{\rm{N}}(0,{I}_{p},D),\,U \sim {\rm{M}}{\rm{N}}({{{\boldsymbol{\mu }}}_{{\rm{m}}}{{\bf{1}}}_{{s}}}^{{\rm{T}}},\varSigma ,{G}),$$

where *Y* is a matrix of promoter activity in log-scale of shape *p *× *s* (where *p* is the number of promoters and *s* is the total number of samples), **μ**_*p*_ and **μ**_*s*_ are promoter-wise and sample-wise means, respectively, **1**_*n*_ is a vector of ones of length *n*, *B* is a matrix of promoter-level motif scores of shape *p* × *m*, *U* is a random matrix of motif activities of shape *m* × *s*, *E* is a random error/noise matrix of shape *p* × *s*, MN is a matrix-variate normal distribution, *I*_*p*_ is an identity matrix of shape *p* ×* p*,* D* is a diagonal matrix of noise variances of shape *s* × *s*, *Σ* is a *m* × *m* matrix of motif variances, *G* is a diagonal *s* × *s* sample-wise scaling matrix, and **μ**_m_ is a motif-wise mean vector of motif activities. In contrast to the classical MARA, modelling **μ**_m_ allows explicitly distinguishing activators from repressors. The number of unique parameters in each of the diagonal matrices *D* and *G* is equal to *g* (the number of groups, in our case, the number of unique samples excluding replicates), which is less than *s* (the total number of samples), which enables an increase in certainty in the parameter estimates of *D*, *G*.

MARADONER performs estimation via a four-stage restricted maximum likelihood procedure. First, we isolate parameters in *E* by finding a transformation that is orthogonal to the **1**_*p*_, **1**_*s*_, *B* matrices, which enables us to focus on estimating *D* solely. Second, we find a transformation that is orthogonal only to the **1**_*p*_, **1**_*s*_ vectors. This makes estimating parameters in *Σ*,* G* possible given the known parameters in *D*. Third, we find a transformation that is orthogonal to *B* only, and we estimate the total mean effect of the ${{{\boldsymbol{\mu }}}_{{p}}{{\bf{1}}}_{{s}}}^{{\rm{T}}}+{{\bf{1}}}_{{p}}{{\boldsymbol{\mu }}}_{{s}}$ term. Finally, given the knowledge of all other parameters, we estimate the mean motif activity vector **µ**_m_. Then, if we disentangle *U* from $B{{{\boldsymbol{\mu }}}_{{\rm{m}}}{{\bf{1}}}_{{s}}}^{{\rm{T}}}$, the deviation from motif mean matrix $\hat{U}=U-B{{{\boldsymbol{\mu }}}_{{\rm{m}}}{{\bf{1}}}_{{s}}}^{{\rm{T}}}$ can be interpreted as a sample-specific variation in motif activity. $\hat{U}$ is then obtained as a maximum a posteriori estimate. Alongside ‘raw’ maximum a posteriori estimates of $\hat{U}$ for downstream analysis, MARADONER also reports standardized motif activities, which are obtained by dividing each motif activity by the square root of its posterior variance.

The availability of the maximum likelihood estimates of motif-specific variances allows for an ANOVA-like test using the asymptotic properties of maximum likelihood estimate for each motif. To this end, MARADONER performs the Wald test by extracting by square root of the diagonal entries from the asymptotic covariance matrix of parameter estimates that correspond to elements from *Σ* (the standard errors).

MARADONER assumes that the gene expression (or promoter activity, in the case of FANTOM5 CAGE data) is provided in the log-scale. As for the motif-scores matrix *B*, by default, each column is normalized by taking the negative logarithm of its empirical survival function.

### TOP and CTOP peak set analyses

To obtain the TOP sites, we first identified thresholds for ChIP–seq peaks, GHT–SELEX peaks and PWM-derived ‘peaks’ (see below) that maximize the three-way Jaccard metric (overlap or union) of the three sets, with the thresholds calculated for each TF independently. We converted PWM matches (derived using MOODS (v.1.9.4)^51^ using a *P* value cutoff of 0.001) into peaks by merging neighbouring matches with a distance less than 200 bp and re-scoring them using the sum-occupancy for clusters. We then identified TOPs as peaks exceeding these thresholds in all three sets and overlap in all three. To obtain the CTOP sites, we then extracted phyloP scores from the Zoonomia consortium^69^ for each base at each TOP site (and 100 flanking bases), removed sites overlapping the ENCODE Blacklist^70^ or protein-coding sequences (owing to the skew in phyloP scores caused by codons) and applied three different statistical tests for significance of phyloP scores over the PWM match: two that tested association between the IC and the phyloP value at each base position of the PWM (using either PCC or likelihood-ratio test), and one that tested for higher phyloP scores over the PWM match (Wilcoxon test). Greater detail on these specific operations is given in the accompanying manuscripts^10,11^, and the custom R (v.4) script for is available at GitHub (https://github.com/imyellan/Codebook_CTOP_scripts).

### Intersection of TOPs and CTOPs and genomic features

We first clustered all CTOPs using BEDTools (v.2.30.0) merge^58^, with a maximum distance of 100 bp, then intersected them with the following genomic feature sets: basic canonical protein-coding promoters from GENCODE (v.44)^71^, defined as 1000 bp upstream and 500 bp downstream of the canonical TSS; the ‘unmasked CpG island’ track, PhastCons Conserved Elements from the Multiz 470 Mammalian alignment, and RepeatMasker track from UCSC^72^; and ChromHMM HEK293 enhancers^11^. We classified promoters as CpG island or non-CpG island on the basis of the GENCODE basic TSS being within ±50 bp of a CpG island from the unmasked track. We classified the CTOP clusters as associated with a single type of genomic feature in the following order of priority: CpG island associated with a protein coding promoter; other CpG islands; a non-CpG island-associated protein-coding promoter; an enhancer; clusters containing a CTCF-binding site but not overlapping a CpG island, promoter or enhancer; overlapping a transposable element and none of the previous categories; overlapping a non-transposable-element repeat and none of the previous categories; and ‘other’ for CTOP clusters not intersecting any examined features.

### Analysis of ASB

We reasoned that the GHT-SELEX and ChIP–seq experiments facilitate direct assessment of ASB of TFs by quantifying the allelic imbalance of read counts at SNVs. We note that the data were not initially intended for this purpose, and caveats include relatively low read counts, linked SNVs and the fact that HEK293 cells have an abnormal karyotype and this cell line was derived from a single individual. Nonetheless, SNV calling (see below) produced 924,997 variant calls overlapping with dbSNP common SNPs (889,814 variant calls from 361 ChIP–seq experiments and 35,183 from 370 GHT-SELEX multicycle experiments) at 122,364 unique genomic locations (corresponding to distinct rsSNP IDs). Of these, 10,009 SNPs corresponded to 12,060 ASBs of 152 Codebook TFs and 39 positive controls. That is, there was a significant imbalance in the number of sequencing reads supporting the reference or the alternative SNP alleles in ChIP–seq (10,575 ASBs) or GHT-SELEX (1,485 ASBs) read alignment (Extended Data Fig. 5 and Supplementary Table 8). SNP calls and ASBs are available at Zenodo^73^ (https://doi.org/10.5281/zenodo.18224872).

### Variant calling

For variant calling directly from ChIP–seq and GHT-SELEX data, we started by mapping raw ChIP–seq and pre-trimmed GHT-SELEX reads^12^ to the hg38 human genome assembly using bwa-mem (v.0.7.1) with default settings (Extended Data Fig. 5a). Next, we used filter_reads.py (originally taken from stampipes (https://github.com/StamLab/stampipes/tree/encode-release), accessed September 2022) to filter out reads with >2 mismatches and mapping quality <10. Then we followed a previously described workflow^21^ for SNV calling and read counting (https://github.com/autosome-ru/MixALime/tree/main/natcomm_supp_scripts, accessed December 2025):

1. samtools reheader (v.1.16.1) was used to set the identical sample SM field in all alignment files;
2. SNP calling was performed using bcftools mpileup (v.1.10.2)^74^ with --redo-BAQ --adjust-MQ 50 --gap-frac 0.05 --max-depth 10000 and bcftools call with --keep-alts --multiallelic-caller;
3. the resulting SNPs were split into biallelic records using bcftools norm with --check-ref x -m - followed by filtering with bcftools filter -i “QUAL>=10 & FORMAT/GQ>=20 & FORMAT/DP>=10” --SnpGap 3 --IndelGap 10 and bcftools view -m2 -M2 -v snps leaving only biallelic SNPs covered by 10 or more reads;
4. SNPs were annotated using bcftools annotate with --columns ID,CAF,TOPMED and dbSNP (v.151)^75^;
5. heterozygous variants located on the reference chromosomes with genotype quality (GQ) ≥ 20, depth ≥ 10 and allelic counts ≥ 5 on each allele were filtered with awk (v.5.0.1);
6. WASP (v.0.3.4)^76^ was used with bwa-mem and filter_reads.py to account for reference mapping bias;
7. count_tags_pileup_new.py (edited version of the original script count_tags_pileup.py from GitHib (https://github.com/vierstralab/nf-allelic-mapping/tree/main/bin), which was adapted by removing the settings related to DNase-seq specifics) was used to obtain allelic read counts with pysam (v.0.20.0);
8. recode_vcf.py was used to convert the resulting BED files to VCF.

Of note, triallelic SNVs were split into two biallelic records.

### ASB calling and annotation

ASB calling was performed independently for GHT-SELEX and ChIP–seq data. To account for aneuploidy and copy-number variation, the profiles of relative background allelic dosage were reconstructed with BABACHI (v.2.0.26) using default settings^77^ (abstract O3). The allelic imbalance was estimated with MIXALIME (v.2.14.17)^21^, starting with mixalime create. Next, we fitted a marginalized compound negative binomial model (MCNB) using mixalime fit specifying MCNB and setting --window-size to 1,000 and 10,000 for GHT-SELEX and ChIP–seq, respectively, taking into account lower coverage and fewer SNPs called from GHT-SELEX. Finally, we used mixalime test followed by TF-wise mixalime combine to obtain the TF-specific ASB calls. This process resulted in 12,060 identified ASBs at 5% FDR (corrected for multiple tested SNPs).

Technically, the GQ of SNPs and ASBs was much higher than the default threshold, and most of the ASB SNPs were supported by two or more datasets (Extended Data Fig. 5d,e).

We then identified 3,564 ASBs that overlapped a PWM hit (*P* < 0.001) for the associated TF. Of note, ASBs that did not overlap a PWM hit may be marker variants acting indirectly by being linked to ‘causative’ SNVs. For ASBs with PWM hits, we calculated the PWM scores for both alleles and estimated the right-tailed *P* values of those scores against a uniform background distribution using PERFECTOS-APE (v.3.0.6)^78^. The fold change between uncorrected alternative (Alt) and reference (Ref) allele *P* values, log_2_[Alt/Ref], reflected the PWM-predicted allelic preferences, with positive or negative values reflecting the preference for the Alt or Ref allele, respectively. ASBs with an absolute log_2_[fold change] > 1 were labelled as ‘motif concordant’ or ‘motif discordant’, depending on whether the allelic preference exhibited by the greater ChIP–seq or GHT-SELEX read coverage (Ref > Alt or Alt > Ref) was consistent with the difference in the respective PWM scores (Extended Data Fig. 5c).

To globally support the relevance of the Codebook ASB calls, we obtained a joint set of 22,064 ASBs by running MIXALIME (v.2.28.0) multiple_combine for joint aggregation of the allelic imbalance *P* values over all processed datasets (Supplementary Table 9). Next, for the resulting joint set of ASB–SNPs, we estimated the significance of the overlap with GTEx (v.8)^24^, ADASTRA (v.6.1)^22^ and EBI GWAS Catalog (v.1, e115_r2025-12-03_full)^23^ using two-sided Fisher’s exact test.

### Reporting summary

Further information on research design is available in the Nature Portfolio Reporting Summary linked to this article.

## Online content

Any methods, additional references, Nature Portfolio reporting summaries, source data, extended data, supplementary information, acknowledgements, peer review information; details of author contributions and competing interests; and statements of data and code availability are available at https://doi.org/10.1038/s41586-026-10798-9.

## Supplementary information

## Source data

## Data Availability

The sequencing raw data for the ChIP–seq, GHT-SELEX and HT-SELEX experiments have been deposited into the Sequence Read Archive database under identifiers PRJEB78913 (ChIP–seq), PRJEB76622 (GHT-SELEX) and PRJEB61115 (HT-SELEX), whereas SMiLE-seq sequencing data are available at ArrayExpress under identifier E-MTAB-14598. Genomic interval information generated for ChIP–seq and GHT-SELEX have been deposited into the Gene Expression Omnibus database under accessions GSE280248 (ChIP–seq) and GSE278858 (GHT-SELEX). PWMs can be browsed at https://mex.autosome.org and downloaded from Zenodo^55^ (https://zenodo.org/records/15667805; https://doi.org/10.5281/zenodo.15667805). The final motifs generated in this study are available in build 3.1 of the CisBP database under study accession ‘Jolma,2026a’. Information on constructs, experiments, analyses, processed data, comparison tracks and browsable pages with information and results for each TF is available at https://codebook.ccbr.utoronto.ca. SNP calls and ASBs are available at Zenodo^73^ (https://zenodo.org/records/18224872; https://doi.org/10.5281/zenodo.18224871). The data underlying MARA are available at Zenodo^61^ (https://zenodo.org/records/17307845; https://doi.org/10.5281/zenodo.17307845). Source data for Extended Data Fig. 5 is available from GitHub (https://github.com/autosome-ru/perspectives-on-codebook-allele-specificity/tree/main/AS_CHS_GHTS/ASB_Extended_Data_Figure_Data_Figure). The list of downloaded PWMs for all Codebook TFs from JASPAR (2024 version)^64^, HOCOMOCO (v.12)^29^ and Factorbook^65^ (downloaded 15 December 2023) is available in Supplementary Table 13. The list of ChIP–seq peaks downloaded from GTRD^62^ and ENCODE (v.4.12.2023)^63^ is available in Supplementary Table 7. FANTOM5 (ref. ^37^) promoter data are accessible in Supplementary Table 12. Zoonomia 241-mammal phyloP scores (Cactus alignment 2020)^69^ and Multiz 471 Mammal alignment PhastCons were used for conservation analysis, downloaded from UCSC^72^. GENCODE (v.44)^71^ was used for the location of coding promoters. GTEx (v.8)^24^, ADASTRA (v.6.1)^22^ and EBI GWAS Catalog (v.1, e115_r2025-12-03 full)^23^ were used for existing SNP databases. Source data are provided with this paper.

## Code availability

The script for adjusting PWM IC, logo_rescale.pl, is available at GitLab (https://gitlab.sib.swiss/EPD/pwmscan). The code for the identification of TOPs and CTOPs is available at GitHub (https://github.com/imyellan/Codebook_CTOP_scripts). The code used for the SNP-centric analysis is available at GitHub (https://github.com/autosome-ru/perspectives-on-codebook-allele-specificity). MIXALIME is available on GitHub (https://github.com/autosome-ru/mixalime). MARADONER is available on GitHub (https://github.com/autosome-ru/MARADONER).
