# Supplementary material for: An expanded codebook of human transcription factor DNA-binding specificity
Source: Nature. 2026 Aug 5;657(8130):275–83. doi: 10.1038/s41586-026-10798-9 (PMC13538051; doi:10.1038/s41586-026-10798-9)
Supplement: Supplementary file 1 [file 41586_2026_10798_MOESM2_ESM.pdf]

nature portfolio

Corresponding author(s): Timothy R. Hughes
Ivan V. Kulakovskiy
Bart Deplancke

Last updated by author(s): May 11, 2026

Reporting Summary

Nature Portfolio wishes to improve the reproducibility of the work that we publish. This form provides structure for consistency and transparency in reporting. For further information on Nature Portfolio policies, see our Editorial Policies and the Editorial Policy Checklist.

Statistics

For all statistical analyses, confirm that the following items are present in the figure legend, table legend, main text, or Methods section.

n/a	Confirmed	
☐	☒	The exact sample size (*n*) for each experimental group/condition, given as a discrete number and unit of measurement
☒	☐	A statement on whether measurements were taken from distinct samples or whether the same sample was measured repeatedly
☐	☒	The statistical test(s) used AND whether they are one- or two-sided<br>*Only common tests should be described solely by name; describe more complex techniques in the Methods section.*
☒	☐	A description of all covariates tested
☐	☒	A description of any assumptions or corrections, such as tests of normality and adjustment for multiple comparisons
☐	☒	A full description of the statistical parameters including central tendency (e.g. means) or other basic estimates (e.g. regression coefficient) AND variation (e.g. standard deviation) or associated estimates of uncertainty (e.g. confidence intervals)
☐	☒	For null hypothesis testing, the test statistic (e.g. *F*, *t*, *r*) with confidence intervals, effect sizes, degrees of freedom and *P* value noted<br>*Give P values as exact values whenever suitable.*
☒	☐	For Bayesian analysis, information on the choice of priors and Markov chain Monte Carlo settings
☒	☐	For hierarchical and complex designs, identification of the appropriate level for tests and full reporting of outcomes
☐	☒	Estimates of effect sizes (e.g. Cohen's *d*, Pearson's *r*), indicating how they were calculated

Our web collection on statistics for biologists contains articles on many of the points above.

Software and code

Policy information about availability of computer code

Data collection	No software was used in data collection

Data analysis

Python 3, R (v.4.3.2), and BEDTools (v2.30.0) are used for various analyses and calculations.
R 'cluster' package (v.2.1.8.1) and Python 'sklearn' (v1.8.0) were used for clustering.
Position weight matrices (PWMs) were identified using numerous tools, described in an accompanying manuscript (Voronstov et al.).
PWM scanning was performed with both MOODS (v1.9.4) and AffiMx (MoSBAT v1).
MoSBAT (v1) was used for PWM comparisons.
ChIP peak calling was performed with MACS2 (v2.2.9.1) .
GHT-SELEX peaks were called using MAGIX: https://github.com/csglab/MAGIX
PWM information content was increased using the logo_rescale.pl script available at https://gitlab.sib.swiss/EPD/pwmscan
Code for triple overlap site identification and conservation analysis is available at https://github.com/imyellan/Codebook_CTOP_scripts
Allele-specific binding analysis was performed using: bwa-mem(v0.7.1), stampipes (accessed Sept. 2022), samtools (v1.16.1),bcftools (v1.10.2), awk (v5.0.1), WASP (v0.3.4), pysam (v0.20.0), BABACHI (v2.0.26), PERFECTOS-APE (v3.0.6), and MIXALIME (v2.14.7 and v2.28.0 for multiple_combine, available at https://github.com/autosome-ru/mixalime).
The code used for the SNP-centric analysis is available at Github:
https://github.com/autosome-ru/perspectives-on-codebook-allele-specificity
Motif activity response analysis and related motif clustering involved SPRY-SARUS (v2.2.3), MACRO-APE (v3.0.6), and MARADONER (v0.13).
The command-line Python reimplementation of MARA (MARADONER) is available at https://github.com/autosome-ru/MARADONER.
IQTree (v3) was used for making maximum-likelihood phylogram).
AlphaFold3 was used for structural predictions.
MAFFT (version 7) L-INS-i was used to make phylogenetic trees derived from DBD sequence alignments.

For manuscripts utilizing custom algorithms or software that are central to the research but not yet described in published literature, software must be made available to editors and reviewers. We strongly encourage code deposition in a community repository (e.g. GitHub). See the Nature Portfolio guidelines for submitting code & software for further information.

Data

Policy information about availability of data

All manuscripts must include a data availability statement. This statement should provide the following information, where applicable:

- Accession codes, unique identifiers, or web links for publicly available datasets
- A description of any restrictions on data availability
- For clinical datasets or third party data, please ensure that the statement adheres to our policy

The sequencing raw data for the ChIP-seq, GHT-SELEX, and HT-SELEX experiments have been deposited into the SRA database under identifiers PRJEB78913 (ChIP-seq), PRJEB76622 (GHT-SELEX), and PRJEB61115 (HT-SELEX), while SMiLE-seq sequencing data is available in ArrayExpress under identifier E-MTAB-14598. Genomic interval information generated for ChIP-seq and GHT-SELEX have been deposited into GEO under accessions GSE280248 (ChIP-seq) and GSE278858 (GHT-SELEX). PWMs can be browsed at https://mex.autosome.org and downloaded at from Zenodo[54] (https://zenodo.org/records/15667805) [doi:10.5281/zenodo.15667805]. The final motifs generated in this study are available in build 3.1 of the CisBP database under study accession “Jolma,2026a”. Information on constructs, experiments, analyses, processed data, comparison tracks, and browsable pages with information and results for each TF is available at https://codebook.ccbr.utoronto.ca. SNP calls and ASBs are available at Zenodo[75] (https://zenodo.org/records/18224872) [doi:10.5281/zenodo.18224871]. The data underlying MARA are available at Zenodo[61] (https://zenodo.org/records/17307845) [doi:10.5281/zenodo.17307845]. Source data for Extended Data Fig. 5 is available from GitHub: https://github.com/autosome-ru/perspectives-on-codebook-allele-specificity/tree/main/AS_CHS_GHTS/ASB_Extended_Data_Figure_Data_Figure. The list of downloaded PWMs for all Codebook TFs from JASPAR[64] (2024 version), HOCOMOCO[29] (v12) and Factorbook[65] (downloaded 15.12.2023) is available in Supplementary Table 13. The list of ChIP-seq peaks downloaded from GTRD[62] and ENCODE (4.12.2023) [63] is available in Supplementary Table 7. FANTOM5[37] promoter data is accessible in Supplementary Table 12. Zoonomia 241-mammal phyloP scores (Cactus alignment 2020)[67] and Multiz 471 Mammal alignment PhastCons were used for conservation analysis, downloaded from UCSC[70]. GENCODE v44[69] was used for the location of coding promoters. GTEx v8[24], ADASTRA v6.1[22], and EBI GWAS Catalog v1 e115_r2025-12-03 full[23] were used for existing SNP databases.

Research involving human participants, their data, or biological material

Policy information about studies with human participants or human data. See also policy information about sex, gender (identity/presentation), and sexual orientation and race, ethnicity and racism.

Reporting on sex and gender	N/A
Reporting on race, ethnicity, or other socially relevant groupings	N/A
Population characteristics	N/A
Recruitment	N/A
Ethics oversight	N/A

Note that full information on the approval of the study protocol must also be provided in the manuscript.

Field-specific reporting

Please select the one below that is the best fit for your research. If you are not sure, read the appropriate sections before making your selection.

☒ Life sciences ☐ Behavioural & social sciences ☐ Ecological, evolutionary & environmental sciences

For a reference copy of the document with all sections, see nature.com/documents/nr-reporting-summary-flat.pdf

Life sciences study design

All studies must disclose on these points even when the disclosure is negative.

Sample size

Experiments were performed on 332 TFs with uncharacterized binding specificities and 61 controls (see Supplementary Table 1 for the list of proteins along with their details). The primary purpose of Codebook was to assess 332 uncharacterized and putative human TFs for sequence-specific DNA-binding activity, and identify their motifs to assemble a complete human TF motif collection.
The uncharacterized TFs were chosen based on the set of human TFs identified in Lambert et al. 2018 (doi: 10.1016/j.cell.2018.01.029) lacking a known binding motif.
The controls were chosen to represent the types of DNA-binding domains found in the uncharacterized TFs, particularly the C2H2-zf domain as 179 of the uncharacterized TFs contained this domain (54%). Thus, 34 of the controls were C2H2-zf TFs.
Each TF was assayed with up to five different binding assays, using up to three different protein sources. A full list of experiments is available in Supplementary Table 4, constructs are listed in Supplementary Table 2, and plasmids are listed in Supplementary Table 3.

Data exclusions

We describe a collection of 4,873 experiments. We excluded 721 experiments from further analysis due to low peak numbers (ChIP-seq and GHT-SELEX experiments). For the remaining 4,152 experiments, a panel of experts reviewed each individual experiment to determine if the experiment was consistent with experiments for the same TF and homologs across replicates and platforms, further details can be found in the Methods section under "Experiment evaluation by expert curation". Expert review resulted in 1,353 experiments deemed successful (1,073 for uncharacterized TFs and 280 for control TFs). Unsuccessful experiments were not included in further analyses, however, all experimental data, whether successful or unsuccessful has been made publicly available (see Data Availability Statement). Supplementary Table 4 lists each experiment along with whether or not it was successful and Extended Data Fig. 1 shows a visualization of the proportion of successful experiments for controls and uncharacterized TFs across experimental platforms and protein sources.

Replication

PBM
PBM experiments have built in replication. In a PBM experiment, two arrays (HK and ME) with differing probe sequences are used, and in order for an experiment to be successful, the same motif must be identified by both. Of the 173 TFs assayed by PBM, 39 successfully produced a motif.

For the remaining 4 assays, multiple replicates were performed for some TFs. Across the study, where replicates were not all successful on the same experimental platform for a given TF, they were determined to be successful due to production of the same motif for the TF across different experimental platforms, and/or due to similarity of the produced motif to homologs.

ChIP-seq
373 TFs were assayed with ChIP-seq. 35 TFs had only 1 replicate. For the 338 TFs with more than 1 replicate, 184 failed in all replicates, 24 had only 1 successful replicate and 130 had 2 or more successful replicates.
375 plasmids were assayed with ChIP-seq. 35 plasmids had only 1 replicate. For the 340 plasmids with more than 1 replicate, 184 failed in all replicates, 24 had only 1 successful replicate and 132 had 2 or more successful replicates.

GHT-SELEX
392 TFs were assayed with GHT-SELEX. 21 TFs had only 1 replicate. For the 371 TFs with more than 1 replicate, 194 failed in all replicates, 61 had only 1 successful replicate and 116 had 2 or more successful replicates.
1,320 plasmids were assayed with GHT-SELEX. 1,118 plasmids had only 1 replicate. For the 202 plasmids with more than 1 replicate, 141 failed in all replicates, 9 had only 1 successful replicate and 52 had 2 or more successful replicates.

HT-SELEX
392 TFs were assayed with HT-SELEX. 21 TFs had only 1 replicate. For the 371 TFs with more than 1 replicate, 191 failed in all replicates, 61 had only 1 successful replicate and 119 had 2 or more successful replicates.
1,330 plasmids were assayed with HT-SELEX. 1,112 plasmids had only 1 replicate. For the 218 plasmids with more than 1 replicate, 154 failed in all replicates, 10 had only 1 successful replicate and 54 had 2 or more successful replicates.

SMiLE-seq
299 TFs were assayed with SMiLE-seq. 146 TFs had only 1 replicate. For the 153 TFs with more than 1 replicate, 102 failed in all replicates, 25 had only 1 successful replicate and 26 had 2 or more successful replicates.
390 plasmids were assayed with SMiLE-seq. 307 plasmids had only 1 replicate. For the 83 plasmids with more than 1 replicate, 46 failed in all replicates, 21 had only 1 successful replicate and 16 had 2 or more successful replicates.

Further details for ChIP-seq, GHT-SELEX, HT-SELEX, and SMiLE-seq replicates can be found in the accompanying papers.

Randomization

ChIP-seq experiments were distributed across batches to balance replicates, experimenters, sequencing runs, TF DNA-binding domain subtypes, and control versus uncharacterized TFs.
Other experiments were performed systematically in batches, ranging from tens of parallel experiments (PBM or SMiLE-seq platforms) to 96-well plate formats used for HT-SELEX and GHT-SELEX. Proteins were assigned largely according to practical considerations such as protein availability and alphabetical order by gene/protein symbol. Formal randomization was not applicable because the study was designed as a systematic survey of DNA-binding activity across many putative TFs rather than as a comparison between randomized treatment groups.

Systematic batching also minimized unnecessary reagent use and experimental handling, and reduced the risk of sample tracking errors or cross-contamination.

Blinding	Researchers were not blinded to the identity of the transcription factors. Blinding is not relevant to the study as identical experimental and data analysis pipelines are used for all transcription factors regardless of whether their identities are known or unknown. In addition, the study did not involve subjective assessment of outcomes or comparison between treatment groups, and the experimental readouts were analyzed systematically. Blinding would have also increased unnecessary reagent use and experimental handling, and increased the risk of sample tracking errors or cross-contamination.

Reporting for specific materials, systems and methods

We require information from authors about some types of materials, experimental systems and methods used in many studies. Here, indicate whether each material, system or method listed is relevant to your study. If you are not sure if a list item applies to your research, read the appropriate section before selecting a response.

Materials & experimental systems

n/a	Involved in the study	
☐	☒	Antibodies
☐	☒	Eukaryotic cell lines
☒	☐	Palaeontology and archaeology
☒	☐	Animals and other organisms
☒	☐	Clinical data
☒	☐	Dual use research of concern
☒	☐	Plants

Methods

n/a	Involved in the study	
☐	☒	ChIP-seq
☒	☐	Flow cytometry
☒	☐	MRI-based neuroimaging

Antibodies

Antibodies used	Anti-eGFP antibody (Ab290, Abcam)
Validation	Anti-eGFP antibody (Ab290, Abcam, https://www.abcam.com/en-us/products/primary-antibodies/gfp-antibody-ab290) was used as the major antibody across all the assays (ChIP-seq, GHT-SELEX and Western blots), with method specific amounts. Each ChIP reaction used 2 µl of undiluted polyclonal antiserum, corresponding to 10 µg total IgG, immobilized on 60 µl of protein G magnetic bead suspension (Dynabead 10004D, ThermoFisher). For HT-SELEX and GHT-SELEX, antibody-bead master mixes were prepared by immobilizing 6 µl antiserum on 100 µl Protein G Sepharose bead slurry (Cytiva, 28-9670-70), and 1 µl aliquots of the resulting mixture were used for each selection, corresponding to approximately 0.06 µl antiserum (300 ng total IgG) per reaction. For western blotting, membranes were incubated in 15 ml antibody solution diluted 1:5,000, corresponding to approximately 15 µg total IgG per membrane.

Eukaryotic cell lines

Policy information about cell lines and Sex and Gender in Research

Cell line source(s)	HEK293 Flp-In T-REx cells 608 (Invitrogen)
Authentication	This Flp-In T-REx HEK293 cell line is a common cell line that contains a recombination site. We successfully obtained hundreds of recombinants. Our cells are isogenic cell lines, each transgenic cell line specifically expressed the specific TF that was inserted to the Flp site after doxycycline treatment (verified by EGFP expression), and further confirmed using the sequencing results.
Mycoplasma contamination	Tested negative by PCR.
Commonly misidentified lines (See ICLAC register)	HEK293 Flp-In T-REx cells are not listed as commonly misidentified cells

Plants

Seed stocks	*Report on the source of all seed stocks or other plant material used. If applicable, state the seed stock centre and catalogue number. If plant specimens were collected from the field, describe the collection location, date and sampling procedures.*
Novel plant genotypes	*Describe the methods by which all novel plant genotypes were produced. This includes those generated by transgenic approaches, gene editing, chemical/radiation-based mutagenesis and hybridization. For transgenic lines, describe the transformation method, the number of independent lines analyzed and the generation upon which experiments were performed. For gene-edited lines, describe the editor used, the endogenous sequence targeted for editing, the targeting guide RNA sequence (if applicable) and how the editor was applied.*
Authentication	*Describe any authentication procedures for each seed stock used or novel genotype generated. Describe any experiments used to assess the effect of a mutation and, where applicable, how potential secondary effects (e.g. second site T-DNA insertions, mosiacism, off-target gene editing) were examined.*

ChIP-seq

Data deposition

☒ Confirm that both raw and final processed data have been deposited in a public database such as GEO.

☒ Confirm that you have deposited or provided access to graph files (e.g. BED files) for the called peaks.

Data access links<br>*May remain private before publication.*	The sequencing raw data for the ChIP-seq experiments have been deposited to the SRA database under identifier PRJEB78913. Genomic interval information generated for ChIP-seq has been deposited into GEO under the accession GSE280248.
Files in database submission	The list of all the files produced are available in Supplementary Table 2 of the accompanying paper (Razavi et al.) and https://codebook.ccbr.utoronto.ca.
Genome browser session<br>(e.g. UCSC)	No longer applicable

Methodology

Replicates	Replicates are outlined in Supplementary Table 2 of the accompanying paper (Razavi et al.)
Sequencing depth	20M reads per sample (targeted depth)
Antibodies	Anti-eGFP antibody (Ab290, Abcam)
Peak calling parameters	MACS2 with sample-specific controls, described in detail in the manuscript.
Data quality	Expert curation and quality control for all the experiments are described in an accompanying manuscript (Voronstov et al.).
Software	MACS2
