# Supplementary material for: An expanded codebook of human transcription factor DNA-binding specificity
Source: Nature. 2026 Aug 5;657(8130):275–83. doi: 10.1038/s41586-026-10798-9 (PMC13538051; doi:10.1038/s41586-026-10798-9)
Supplement: Supplementary file 2 [file 41586_2026_10798_MOESM1_ESM.docx]

**An expanded codebook of human transcription factor DNA binding specificity**

Arttu Jolma**^1,15^**, Kaitlin U. Laverty**^1,2,3,15^**, Ali Fathi**^1,3,15^**, Ally W.H. Yang**^1,15^**, Isaac Yellan**^1,3,15^**, Ilya E. Vorontsov**^4,15^**, Antoni J. Gralak**^5,6^**, Judith F. Kribelbauer-Swietek**^5,6,7^**, Sachi Inukai**^5,6^**, Rozita Razavi**^1^**, Mihai Albu**^1^**, Alexander Brechalov**^1^**, Zain M. Patel**^1,3^**, Vladimir Nozdrin**^8^**, Georgy Meshcheryakov**^9,10^**, Andrey Buyan**^9,10^**, Ivan Kozin**^4,8,9^**, Sergey Abramov**^4,11^**, Alexandr Boytsov**^4,11^**, The Codebook Consortium*, Quaid D. Morris^2^, Matthew T. Weirauch**^12^**, Oriol Fornes**^13^**, Vsevolod J. Makeev**^4,#^**, Jan Grau**^14^**, Ivo Grosse**^14^**, Philipp Bucher**^6^**, Bart Deplancke**^5,6^****, Ivan V. Kulakovskiy**^4,9,10^****, and Timothy R. Hughes**^1,3^****

**^1^**Donnelly Centre, University of Toronto, Toronto, ON M5S 3E1, Canada

**^2^**Computational and Systems Biology Program, Sloan Kettering Institute, Memorial Sloan Kettering Cancer Center, New York, NY 10065, USA

**^3^**Department of Molecular Genetics, University of Toronto, Toronto, ON M5S 1A8, Canada

**^4^**Vavilov Institute of General Genetics, Russian Academy of Sciences, 119991, Moscow, Russia

**^5^**Laboratory of Systems Biology and Genetics, Institute of Bioengineering, School of Life Sciences, École Polytechnique Fédérale de Lausanne, 1015, Lausanne, Switzerland

**^6^**Swiss Institute of Bioinformatics, 1015, Lausanne, Switzerland

**^7^**Department of Biological Sciences, University of Southern California, Los Angeles, CA, USA

**^8^**Faculty of Bioengineering and Bioinformatics, Lomonosov Moscow State University, 119991, Moscow, Russia

**^9^**Institute of Protein Research, Russian Academy of Sciences, 142290, Pushchino, Russia

**^10^**Institute of Biochemistry and Genetics, Ufa Federal Research Centre of Russian Academy of Sciences, 450054, Ufa, Russia

**^11^**Altius Institute for Biomedical Sciences, Seattle, WA 98121, USA

**^12^**Center for Autoimmune Genomics and Etiology, Cincinnati Children’s Hospital Medical Center, Cincinnati, OH, USA; Divisions of Allergy and Immunology, Biomedical Informatics, Human Genetics, and Developmental Biology, Cincinnati Children’s Hospital Medical Center, Cincinnati, OH, USA; Department of Pediatrics, University of Cincinnati College of Medicine, Cincinnati, OH, USA

**^13^**Department of Medical Genetics, Centre for Molecular Medicine and Therapeutics, BC Children’s Hospital Research Institute, University of British Columbia, Vancouver, BC V5Z 4H4, Canada

**^14^**Institute of Computer Science, Martin Luther University Halle-Wittenberg, 06099, Halle, Germany

**^15^**These authors contributed equally

*A list of authors and their affiliations appears at the end of the paper.

**^#^**Present address: Cancer Research UK National Biomarker Centre, University of Manchester, Manchester, M20 4BX, UK

**To whom correspondence should be addressed: [bart.deplancke@epfl.ch](mailto:bart.deplancke@epfl.ch), ivan.kulakovskiy@gmail.com, t.hughes@utoronto.ca

[Article I.	Supplementary Discussion	3](#_Toc231818703)

[**Section 1.01**	**Supplementary Note 1. Under-characterized DBDs, including the SKI/SNO/DAC and C-Clamp domains**	3](#_Toc231818704)

[Section 1.02	Supplementary Note 2.Transposon-derived TFs	7](#_Toc231818705)

[**Section 1.03**	**Supplementary Note 3. Putative TFs that were unsuccessful in Codebook experiments (“Unsuccessful TFs”)**	11](#_Toc231818706)

[**Section 1.04**	**Case by case re-assessment of unsuccessful putative TFs**	15](#_Toc231818707)

[(a)	Description	15](#_Toc231818708)

[(a)	C2H2 zinc finger proteins that are unlikely to be sequence specific TFs	16](#_Toc231818709)

[(b)	C2H2 zinc finger proteins that are potential sequence specific TFs	23](#_Toc231818710)

[(c)	Unsuccessful proteins with canonical C2H2 arrays (potential TFs)	27](#_Toc231818711)

[(d)	Other known-DBD-containing proteins that are unlikely to be sequence specific TFs	28](#_Toc231818712)

[(e)	Other known-DBD-containing proteins that are potential sequence specific TFs	36](#_Toc231818713)

[(f)	Proteins with unknown DBDs that are unlikely to be sequence specific TFs	42](#_Toc231818714)

[(g)	Potential TFs with unknown DNA-binding domain	52](#_Toc231818715)

[**Section 1.05**	**SUPPLEMENTARY TABLE TITLES AND LEGENDS**	55](#_Toc231818716)

[**Section 1.06**	**Supplementary information references**	57](#_Toc231818717)

# Article I. **Supplementary Discussion**

## Section 1.1 **Supplementary Note 1. Under-characterized DBDs, including the SKI/SNO/DAC and C-Clamp domains**

**Introduction**

Six Codebook proteins that lacked canonical DBDs yielded successful experiments, and therefore motifs (CGGBP1, NACC2, TCF20, PURB, DACH1, and DACH2). All six appear to represent DBDs that were poorly described at the outset of the Codebook study. We and others have recently described CGGBP1 as the founding member of an extensive family of eukaryotic TFs derived from the DBDs of transposons, which may be related to the BED-type Zinc Finger found in ZBED TFs^87,88^. NACC2 contains a BEN domain, which over the last decade has been established as typically a sequence-specific DBD^89-91^. TCF20 contains a potential AT-hook^92^ (below the conventional Pfam scoring threshold) and yielded an AT-hook-like motif. PURB is composed largely of three copies of the PUR (Purine-rich-element binding) domain; it yielded a motif on four different PBM assays and in one independent SMiLE-seq experiment after additional analysis^13^ (resembling ACCnAC/GTnGGT), which is unlike its previously established binding site (CTTCCCTGGAAG)^93^. The sequence specificity of this protein thus remains enigmatic.

DACH1 and DACH2 are paralogs that yielded very similar motifs. They contain a SKI/SNO/DAC domain, a winged helix-turn-helix (HTH)-type DBD shared with their *Drosophila* *melanogaster* ortholog Dachshund. Members of this family have been previously described as binding to specific DNA sequences: a Forkhead-like motif (different from the one we obtained) was previously described for human DACH1^94^, and human SKI was reported to bind GTCTAGAC in SELEX^95^. The SKI/SNO/DAC domain is also believed to possess other, non-DNA binding biochemical functions, however. For example, the SKI/SNO/DAC of *D. melanogaster* Dac is necessary for transcription activation in yeast two-hybrid assays, potentially implying a protein-protein interaction function^96^. Previous structural and biochemical analysis of the SKI/SNO/DAC domain of SKOR1/2 also suggests that in these proteins the domain has a protein-interaction function^97,98^

In addition to these six examples, the sequence specificity of SLC2A4RG and ZNF395 – both Codebook proteins with only a single C2H2-zf – resides in their C-clamp, rather than the C2H2-zf. The C-clamp is also present in ZNF704, which has a published HT-SELEX PWM that is virtually identical to that of SLC2A4RG and ZNF395 (resembling the sequence CCGGCCGG)^80^. The C-clamp domain is perhaps best known for its presence in two members of the TCF/LEF family in humans, in which it augments the DNA sequence specificity of the HMG domain^99^. The C-clamps in ZNF704, ZNF395, and SLC2A4RG have also been previously described as binding to shorter, unmethylated sequences containing mainly C and G^100,101^, most typically “CCGG”. These sequences are also enriched in the Codebook datasets, but the apparent dimeric site “CCGGCCGG” is even more preferred in several different assays^12^.

**SKI/SNO/DAC and C-clamp potentially represent expansive families of transcription factors**

***SKI/SNO/DAC.*** Both of the last two domain classes (SKI/SNO/DAC and C-clamp) are distributed broadly across animals, and thus may represent large classes of unexplored DBDs. We therefore sought to further characterize their phylogenetic distribution, the structural basis of their ability to bind DNA, and the DNA-binding specificities of putative transcription factors encoding these domains across animals.

Interpro^102^ lists over 7,000 proteins containing a SKI/SNO/DAC domain, entirely in metazoans. DACH1 and DACH2 orthologs are prevalent across vertebrates, with many duplicates retained in salmonids due to their lineage-specific whole-genome duplication (**Extended Data Fig. 2a**). Phylogenetic analysis of SKI/SNO/DAC domains reveals four major groups. DACH (containing DACH1/2, and invertebrate Dac), SKOR (SKOR1/2 in vertebrates and *D. melanogaster* Fuss), and SKIDA1, which appears to be restricted to vertebrates, are clearly defined (**Extended Data Fig. 2b**). Vertebrate SKI and SKIL and *D. melanogaster* Snoo appear to be early diverging genes; more detailed analysis would be necessary to confidently state that they form a clade, but they are more similar to each other than to members of the other three groups.

We tested a representative set of 21 SKI/SNO/DAC domains across animals, as well as all seven human proteins encoding these domains, using HT-SELEX. DACH1 and DACH2 yielded the same motifs as previously. SKIDA1 also produced a clear DNA-binding motif (**Extended Data Fig. 2b**). In addition, while SKI failed in our follow-up assays, its paralog SKIL was among the original Codebook set, in which it produced a PWM in only a single HT-SELEX experiment, and thus did not pass our quality control. The PWM generated in this assay is, however, concordant with the SELEX-derived consensus sequence GTCTAGAC by Nicol et al. 1998 for SKI, from only 33 SELEX ligands^95^. Both our SKIL experiment and the Nicol et al. data are based on cellular extracts, and as these proteins were unsuccessful with other protein production methods, they may require a post-transcriptional modification, bind as heterodimers with an unknown protein, or potentially contact DNA indirectly. None of the other SKI/SNO/DAC family members yielded a clear positive result.

In support of direct DNA binding by at least a subset of the SKI/SNO/DAC class, Alphafold3^103^ predicted that the DACH1 HTH inserts into the major groove precisely at the PWM-predicted binding site within an extended DNA sequence (**Extended Data Fig. 2c, left**). We also modeled full-length and SKI/SNO/DAC-only SKI and SKIL protein sequences with a sequence from Nicol et al 1998^95^ in AlphaFold3, but the molecules were not predicted to interact. By contrast, the SKIDA1 SKI/SNO/DAC was predicted (albeit with a low iPTM = 0.26) to interact with a high-scoring HT-SELEX sequence at precisely its motif, with a similar configuration as DACH1 (**Extended Data Fig. 2c, right**).

***C-clamp.*** Interpro^102^ lists over 8,000 proteins containing the SMART^104^ C-clamp domain, entirely in metazoans and a handful of closely related protists. Ensembl has called one-to-one orthologs across most animals for all but one of the human C-clamp containing proteins (**Extended Data Fig. 2d**), again with whole-genome derived duplicates in salmonids across most of the genes. By contrast, SLC2A4RG appears to be a tetrapod-specific paralog, as synteny-supported one-to-one orthologs only exist through to amphibians in Ensembl^105^. Phylogenetic analysis of the C-clamp from human and representative non-human organisms clearly divides it into two major clades – the Glut4EF/ZNF395 family containing ZN704 and SLC2A4G, and the LEF/TCF family (**Extended Data Fig. 2e**).

Like SKI/SNO/DAC, we assayed a representative set of C-clamp domains on HT-SELEX, selected from diverse animals as well as all five human proteins encoding C-clamps. Of the 17 proteins tested, we successfully derived 9 high-quality C-clamp-like motifs (**Extended Data Fig. 2e**). The TCF-proteins were tested with both the C-clamp alone, the HMG alone, and the full-length protein including both domains.

The C-clamp domains of human TCF7 and TCF7L2 both failed when assayed alone. The full-length protein for TCF7L2, however, did derive both a clear HMG motif as well as a C-clamp-like motif (A/G)CCG, albeit at a lower enrichment (**Extended Data Fig. 2e**). We did not derive a compound motif containing the signatures of both DBDs, but the enrichment of the C-clamp motif is consistent with its involvement in binding “helper sites” with the consensus (A/G)CCG near HMG motifs, with the C-clamp’s inclusion in some TCF isoforms modulating binding site usage downstream of Wnt signaling^99^. The failure to enrich a bipartite motif with static spacing may be due to flexibility in orientation and distance between the HMG and helper motifs^106^. The C-clamp domain alone from *C. elegans* Pop-1 (a TCF-like protein) binds a nearly identical motif to the C-clamp motif obtained from the longer TCF7L2 construct, however, consistent with the deep conservation of Wnt signaling across animals, and the importance of the bipartite motif in activation of downstream targets^107^.

An Alphafold3-predicted structure of SLC2A4RG is consistent with the C2H2-zf not serving as the main determinant of DNA-binding, and instead with the C-clamp(s) binding the major groove precisely at the PWM-predicted binding site(s) within an extended DNA sequence (**Extended Data Fig. 2f**). The predicted structure is virtually identical to the experimentally determined structure: the SLC2A4RG C-clamp in complex with a single GCCG-containing sequence^100^ possesses the same configuration a monomer, with the four cysteine-residues in the C-clamp domain coordinating a Zn2+ ion. A unique contribution of our predicted structure is that the C2H2 zinc-finger may contact the major groove, potentially stabilizing the interaction. Several of our tested C-clamp constructs exclude the C2H2-zf, however (see next section), so at least in the case of the GlutEF/ZNF395 family, the C-clamp is sufficient for high-affinity binding.

An Alphafold3-predicted structure of SLC2A4RG is consistent with the C2H2-zf not serving as the main determinant of DNA-binding, and instead with the C-clamp(s) binding the major groove precisely at the PWM-predicted binding site(s) within an extended DNA sequence (**Extended Data Fig. 2f**). The predicted structure is virtually identical to the experimentally determined structure: The SLC2A4RG C-clamp in complex with a single GCCG-containing sequence^100^ possesses the same configuration a monomer, with the four cysteine-residues in the C-clamp domain coordinating a Zn2+ ion. A unique contribution of our predicted structure is that the C2H2 zinc-finger may contact the major groove, potentially stabilizing the interaction. Several of our tested C-clamp constructs exclude the C2H2-zf, however (see **Supplementary Note 3**), so at least in the case of the GlutEF/ZNF395 family, the C-clamp is sufficient for high-affinity binding.

## Section 1.2 Supplementary Note 2.Transposon-derived TFs

**Introduction**

Domestication is the process by which a transposon coding sequence is exapted by the host through fusion to an existing protein (often called ‘exonization’ as a TE exon is captured by a host gene), or as an entirely new gene^108^. The most widespread and consistent path of TE contribution to transcription factors is in the form of DBDs derived from DNA transposons^43^. In the transposon, this DBD is needed to identify the cognate genome-integrated transposon in *trans*, typically by recognizing motifs in the transposon’s Terminal Inverted Repeats (TIRs), which flank the transposase ORF. TE-derived DBDs, now within host genes, have been and continue to be identified across animals.

We identified new DNA-binding motifs for sixteen previously known domesticated Codebook proteins (and two controls), with previously putative TF function. These include CGGBP1 from *hAT-19*^87^, five proteins containing BED-zf domains derived from *hAT-Ac* and *hAT-Charlie*^109^, six with the related CENBP or Brinker domains from *Tigger* and *Pogo* elements^110^, three with *PIF-Harbinger*-derived Myb/SANT^111^ or MADF domains, and FLYWCH1 from a *Mutator*-related transposon^112^. We therefore sought to characterize the evolutionary relationships of these proteins, as well as investigate their interactions with genomic transposon fragments.

**Pogo and Tigger-derived transcription factors**

The *Tigger*/*Pogo*-derived TFs with new Codebook motifs are involved in a range of biological processes and implicated in disease^113-115^. They include TIGD3, 4, 5, and 7, JRK, and the more distantly related POGK. All of these proteins share a bipartite DBD comprised of two HTH domains, with the N-terminal domain being of the “Pipsqueak (Psq)” family^116^ in Interpro^102^, and the C-terminal domain simply a “CENPB-type HTH”^117^. In POGK, the Psq domain is replaced with a “Brinker”-type HTH^118^, but retains the C-terminal CENPB-type HTH. Based on phylogenetic analyses, *Tigger*/*Pogo* have been subject to multiple independent domestication events across tetrapods, although the precise evolutionary histories remain uncertain^83,84^. TIGD7 and JRK do at least seem to be closely related, and may have arisen from a single domestication event in the common ancestor of mammals^110^. The PWMs obtained for *Tigger*/*Pogo*-derived TFs are often long and information-rich, which may be one strategy by which their ancestral TEs bound specifically to cognate elements integrated across a large genome (**Extended Data Fig. 9a**).

All DNA transposons, including *Tigger*/*Pogo*, have been extinct in the human lineage for over 40 million years^111^. Nonetheless, genomic binding of JRK is enriched for binding to a subset of *Tigger*/*Pogo* elements, particularly Tigger15a, and the consensus sequence for Tigger15a has a PWM-predicted binding site for JRK in the TIRs of these elements (**Extended Data Fig. 9b**), consistent with its presumed ancestral role in transposition. A TBLASTN search of the JRK protein sequence against Dfam^119^ *Tigger/Pogo* consensus DNA sequences also reveals that JRK has the strongest sequence homology to Tigger15a, suggesting that Tigger15a is the family, or is closely related to the family, from which JRK (and perhaps TIGD7) was domesticated. To our knowledge, it is only the third human protein known to still bind its ancestral transposons, including SETMAR, derived from a fusion in anthropoid primates between a *Tc1-mariner* (a distantly related superfamily to *Tigger/Pogo*) transposon and a SET domain^46^, and JRK’s relative CENPB, as the CENPB box has been noted to resemble the Tigger2 transposon’s TIRs^120^. These cases apparently represent the simultaneous introduction of a multitude of *cis*-regulatory elements and the TF that binds them, which may be a mechanism by which many transposons have been domesticated as TFs, such as the bat KRABINER^121^.

**BED-type zinc fingers are universal DNA-binding domains**

Phylogenetic analysis of the ZBED family of transcription factors suggests that they are derived from at least three different domestication events: ZBED1 from *hAT-Ac,* ZBED2, 3, 4, 6 from a different *hAT-Ac* domestication, and ZBED5, 7 (aka ZMYM6), 8 (aka FAM200C), and 9 (aka SCAND3) from a distinct family, *hAT-Charlie*^109^. Of these, only ZBED1 and ZBED6 have been closely investigated for DNA-binding activity, binding TGTCG(C/T)GA(C/T)A and GCTCG consensus sequences, respectively^85,122^. Codebook tested all remaining ZBEDs other than ZBED7 and 8, and in addition, tested the BED-zf domains of FAM200B, a poorly characterized protein closely related to ZBED9, and GTF2IRD2, which is a fusion of two GTF2I DNA-binding domains with an N-terminal *hAT-Charlie*^123^.

All yielded high-quality motifs save for ZBED3, consistent with a previous study that found it to have a largely cytosolic localization, instead functioning as a Wnt signaling regulator via direct interaction with Axin^124^. CGGBP1 possesses a BED-zf-like DBD and is domesticated from *hAT-19* transposons, which form a distinct family from *Ac* and *Charlie*^87,88^. A phylogeny created using zf-BED and zf-BED-like domains from these domesticated TFs clearly recapitulates the known relationships among the three *hAT* families, as well as the evolutionary domestication histories of the ZBEDs (**Extended Data Fig. 9c**).

Like *Tigger/Pogo*-derived TFs, the *hAT*-derived TFs exhibit broad motif diversity both in sequence and length, although they often feature a CpG dinucleotide followed by 1-2 A’s and ~3 C’s (**Extended Data Fig. 9c**). Unlike *Tigger/Pogo*, most BED-zf motifs are short, between 6-7 nucleotides; this may reflect how *hAT* transposases recognize and bind their cognate integrated transposons, with the BED-zf binding subterminal repeats distributed at both ends of the transposon, between the TIRs and transposase ORF^125^.

Interestingly, while GTF2IRD2 has GTF2I DBDs, assays using constructs containing just the GTF2I DBDs failed, while full-length protein constructs including the BED-zf were successful. The derived motif is also reminiscent of the other *hAT-Charlie*-derived DBDs (**Extended Data Fig. 9c**); these lines of evidence suggest that it is, in fact, the BED-zf domain of GTF2IRD2 that is conferring sequence-specific binding.

**Origin of *PIF-Harbinger*-derived transcription factors**

While the origins of several functionally diverse classes of DNA-binding proteins can be attributed to domestication from transposons^43^, some of these DBDs may have been originally acquired from a host gene through a very ancient domain capture event. For example, the Myb domain, a tri-helical HTH, is characteristic of *PIF-Harbinger* elements^126^ and of *PIF-Harbinger*-derived TFs, but is also widely distributed across eukaryotes in genes with no known homology to TEs^127^. As a result, it cannot be confidently stated yet whether all Myb DBDs are derived from *PIF-Harbinger*, or if only a subset are.

Codebook tested a number of Myb/SANT encoding proteins, including DMTF1, TERF1, TTF1, MYSM1, TERB1, MSANTD1, MSANTD4, MYPOP. However, only the latter three have homology to *PIF-Harbinger* transposons^111^, although all three yielded high-confidence motifs (**Extended Data Fig. 9d**). We have also previously derived motifs for the *PIF-Harbinger-*derived MSANTD3 and NAIF1^128^ (the NAIF1 motif is also consistent with a previously identified consensus binding sequence^129^). A unique characteristic of *PIF-Harbinger* transposons is that the Myb DBD and the catalytic domain are encoded on separate ORFs^130^. In all of the TFs domesticated from *PIF-Harbinger,* the protein encodes the Myb DBD with no detectable catalytic domain remnants (unlike many of the *hAT* and *Tigger/Pogo* TFs above), which is perhaps mediated by it being encoded on a separate ORF that can be exonized into another protein, or domesticated in isolation.

**Structural prediction of FLYWCH1-DNA binding**

FLYWCH1 encodes five FLYWCH-type zinc-fingers, DBDs that are encoded by the *Phantom* family of *Mutator* DNA transposons^112^. We revealed that FLYWCH1 binds to a degenerate motif, with two high information content CpG dinucleotides, and two lower information content ones (**Extended Data Fig. 9d**). FLYWCH1 had poor overlap between ChIP-seq, GHT-SELEX, and PWM hits, and all TOPs are restricted to a ~6kb region on chromosome 10, and does not have enrichment for promoters, enhancers, or the “dark genome” in our datasets. FLYWCH1 has been tentatively identified as a tumour suppressor and DNA-damage response protein in a handful of studies^131-133^, and may be involved in the recruitment of H3K9me3-associated proteins^134^. We therefore sought to gain a clearer understanding of FLYWCH1 binding activity, which could drive further study into its functional role in chromatin regulation.

While an unpublished NMR solution structure of the fifth FLYWCH1 zinc-finger exists (PDB: 2RPR), it is unclear which zinc-fingers are essential for conferring DNA-binding. To generate an initial hypothesis of how FLYWCH1 interacts with its binding site, we generated an Alphafold3^103^ structure of the five FLYWCH1 zinc fingers with a high-confidence TOP sequence. While the pTM and iPTM scores are both low (0.29 and 0.33 respectively), the overall structure is consistent with our observed motif, with zinc-fingers 4 and 5 inserting into major grooves containing the highest-information content CpG dinucleotides (**Extended Data Fig. 9e**), and zinc-fingers 1 and 2 interacting with much lower-information positions, which at this binding site encode slightly suboptimal GG dinucleotides. As a CpG-binding protein, binding-site affinity might be influenced by DNA methylation, which may partially explain the low overlap between ChIP-seq and GHT-SELEX. FLYWCH1 SMiLE-seq assays were unsuccessful, so it is currently unclear how DNA methylation may influence the binding activity of the protein^13^.

**Codebook TFs are frequently enriched for binding specific families of transposons**

The Codebook data also underscore that many TFs bind preferentially and intrinsically to specific repeat classes. These interactions are explored in greater detail in the accompanying manuscripts^10,11^. Binding to endogenous retroelements is known to be a common property of the KRAB-domain-containing C2H2-zf (KZNF) subfamily in vivo^41^, but until now it has not been clear that the recruitment is defined almost entirely by the sequence specificity of the KZNFs alone. The combination of assays, particularly GHT-SELEX, extends earlier observations by pinpointing the exact binding sites and demonstrating that these proteins typically have high specificity for these elements, because they bind preferentially to precisely the same elements *in vitro*. Binding preferentially to retroelements is not limited to KZNFs but includes other C2H2-zf proteins and other classes of TFs. For example, binding sites for ZBED9 are enriched for binding to Alus, and some *hAT-Charlie* elements^11^, the family from which it was originally domesticated^109^.

## Section 1.3 **Supplementary Note 3. Putative TFs that were unsuccessful in Codebook experiments (“Unsuccessful TFs”)**

Here, we discuss potential reasons for the failure of 155 Codebook proteins to yield motifs. We first examined overall trends observed amongst the unsuccessful proteins, which we discuss here, and then consider each protein individually. By extension from the success rate for positive controls (58/61, or 95%), we would expect 314 successes out of the 332 Codebook proteins but obtained only 177. Thus, we sought explanations for the remaining 155 (= 332 - 177).

We first broke the 155 proteins into three main classes (**Extended Data Fig. 3a**), which we consider in turn: the C2H2-zfs (59 failures out of 179 attempted), all other known DBDs (53 failures out of 104 attempted), and those lacking a known DBD (43 failures out of 49 attempted). Each class has specific characteristics: the C2H2-zfs are best known for binding as tandem arrays, other DBDs generally have individual known structural characteristics (and most are represented by known structures), while those lacking a known DBD are mainly isolated cases with limited support in the literature and no DNA-protein structures.

The Codebook Consortium analysis was based on our previous curation of potential TFs, which we published in the database “HumanTFs”^1^, and as our purpose was to compile a list for direct experimental evaluation of their DNA binding capability, our inclusion criteria were lenient. A large fraction of the proteins were included simply based on homology (typically, because they contained a likely DNA-binding domain), even if there was no direct evidence and on the other hand, we included proteins even if their sequence-specific DNA binding activity was based only on a single study and there was no other supporting evidence. Proteins that were included based on homology had overall high success rates, whereas proteins based on scant evidence were unsuccessful in all but a few notable exceptions.

**C2H2-zf proteins**

The C2H2-zf proteins typically bind DNA as a tandem array with each C2H2-zf offset from the next by three bases. This arrangement places constraints on the “linker” in between the C2H2-zf domains. Roughly half of all linkers in human C2H2-zf arrays are seven amino acids in length. Consistent with the importance binding as a tandem array with appropriate spacing, Codebook C2H2-zf proteins that contained at least a pair of DBDs separated by 3-10 amino acids showed a high success rate (112/132, or 84.9%), unlike those with only noncanonical gaps (4/14, or 28.6%) and those with only a single C2H2-zf domain (5/21, or 23.8%). Furthermore, the experiments were unsuccessful for 9 proteins that contain only “unconventional” C2H2-zf domains, which were included based on literature evidence of containing a C2H2, but that did not pass HMMer-based prediction as used in CisBP (P-value of less than 10^-5^) (**Extended Data Fig. 3b**).

We then manually examined all of the C2H2-zfs in these three categories that failed. For a minority, there is credible evidence in the literature that they may bind DNA (**Supplementary Table 6, Supplementary Document 1, and Extended Data Fig. 3c**). For 21 of them, however, there is insufficient evidence and we have classified these as unlikely to be TFs.

Considering the low success rates for non-array C2H2 proteins, we examined, case-by-case, the successful proteins in this category. There were five proteins with only a single C2H2-domain that succeeded in yielding a motif; two of them (SLC2A4RG and ZNF395) appear to use the adjacent C-clamp to bind DNA (discussed above). Another two (ZNF503 and ZNF703) are close paralogs that were successful only in ChIP-seq experiments. According to previous data, ZNF703 is a chromatin corepressor component that may not bind DNA directly, but through other proteins^135^; we note that the ChIP-seq experiments enriched posterior homeodomain reminiscent CATAAA sites and several other motifs in both experiments. The last, ZBTB5, is likely to be a genuine TF that binds DNA specifically with just a single C2H2 domain by recognizing combinations of palindromic GATC-sequences. BTB domains are known to mediate homodimerization, suggesting that the domain manages to bind DNA with a single DBD through cooperative binding. Thus, it represents an unusual case.

Analyses resulted in four successful proteins with noncanonical gaps. MYT1L represents a well-characterized exception known through analysis of its paralog MYT1. Its C2H2 domains are of a specific C2H2C (IPR036060) subtype, that are known to be able to bind DNA either individually or as cooperative combinations with a different kind of mechanism than what is used in array based C2H2 proteins – the two domains do not enter major groove as an array, but instead the long disordered region separating them allows the domains to bind opposite to each other and recognizing a palindromic consensus sequence^136,137^. The second protein, TSHZ2, contains a homeodomain in addition to its widely gapped C2H2 domains (that are folded in the expected manner based on AlphaFold3), and the ATCA-consensus that it binds is similar to the sites bound by PBX homeodomains. ZNF618 is a poorly characterized protein that contains 4 canonical (and consistently folded on AlphaFold3) C2H2 domains, and additionally an enzymatic domain belonging to the Ribonuclease H-like domain family. The Codebook evidence is from lysate-based SELEX experiments and could potentially represent indirect binding. Finally, CASZ1 represents an interesting case, as it recognizes very long target sites with gapped A or T stretch PWMs. Both CisBP and protein domain databases predict that this protein has 11 canonical C2H2 domains separated by noncanonical gaps; however, AlphaFold3 predicts that all the repetitive domains predicted as C2H2 are in fact noncanonical C2H2 protein domains with a more complex fold. When compared against canonical DNA-binding C2H2 domains, they have an additional beta-sheet (i.e., three rather than two antiparallel beta-sheets), and there is a long linker between the first two beta-sheets that folds as part of the structure. Additionally, these domains have a longer putative DNA recognition helix than what is present in typical DNA-binding C2H2 domains. Conservation analysis of CASZ1 previously detected the sequence features that fold into these domains and predicted them as a novel type of zinc finger domain called “para-ZNF”^138^ but this domain type does not appear to be listed in databases.

Overall, 21 out of the 59 failures of C2H2-zf protein can be explained by either the number, type, or spacing of the C2H2-zfs.

**Other DBD families**

Fifty-three (53) of the unsuccessful proteins had a predicted protein domain of a type that has been shown to bind DNA in sequence specific manner in either the same protein or homologous proteins (**Extended Data Fig. 3a**). For most of these DBDs, there are DNA-protein structures and other biochemical data available for either the same protein, or others of the same DBD class. We used these data, as well as Alphafold3 predictions, and other literature, to assess whether the unsuccessful proteins have properties that are consistent with sequence-specific DNA binding.

In many cases, the unsuccessful proteins had features that would likely prevent them from engaging in sequence-specific binding. For example, the RFX domain in RFX8 is truncated and missing a DNA-contacting region. SGSM2 is annotated as containing a BED family ZF; however, Alphafold3 predicted that the putative ZBED domain is not folded into a ZBED but is a part of a larger folded protein domain. Thus, these proteins are likely unable to bind DNA in a sequence-specific manner. In addition, some of the unsuccessful Codebook proteins now have motifs in databases and other studies in the literature.

We assessed all 59 proteins on the basis of available information, and gauged whether each appeared most likely to be a sequence-specific TF (24 cases), a low-specificity DNA-binding protein and/or cofactor (20 cases), or neither (9 cases).

Altogether there are 24 potential false negatives and 29 apparent true negatives, among the “Other” DBD families. Nine that we classified as true negatives represent the THAP domain, which appears to have both low DNA affinity and low DNA sequence specificity, and seven have the AT-hook DNA binding peptide motif, which has low sequence specificity and is often found in cofactors and chromatin remodeling enzymes **(Extended Data Fig. 3c)^139^**.

**“Unknown” DBD proteins**

We applied the same criteria as for the “Other” DBD class to those with no known DNA-binding domain. Most of these proteins (34/43) had been included in the Codebook study on the basis of a single previous publication, typically including SELEX and/or EMSA. For most of these (30/34), no additional evidence has since emerged in support of sequence-specific DNA binding, while some now have other known functions; e.g. recombinant KCNIP3 was reported to bind DNA by EMSA^140^, but it has a well-established role in binding and controlling specific potassium channels^141^.

Ten of the 43 do appear to represent likely sequence-specific DNA-binding proteins (**Supplementary Table 6** and the following Case by case re-assessment). Upon re-examination of our own data, several do appear to display sequence specificity, but did not pass our stringent success criteria. These include RAG1 and SKIL. Most of the remainder are believed to require cofactors to^21,71^ bind DNA. For example, PDB:4NDY shows CENPS and CENPX binding DNA in an intertwined and asymmetric manner, consistent with binding as an obligate heterodimer. The Codebook *in vitro* experiments were performed with single proteins; thus, failure of these proteins could easily represent false negatives. Most (33/43) appear more likely to represent true negatives.

**Summary**

Compiling the results above, we can explain 21+29+33 = 83 of the 155 unsuccessful Codebook TFs as likely to be true negatives (54%). For others, the expert curation identified explanations for false negatives (e.g., obligate cofactors), which will guide future efforts to obtain high-quality PWMs for these proteins.

## Section 1.4 **Case by case re-assessment of unsuccessful putative TFs**

### (a) Description

This analysis contains case-by-case manual assessment of the proteins that did not yield successful experiments in the Codebook Consortium project. They are organized by DNA-binding domain (DBD) composition and result of the re-assessment.

Assessment objective is to see whether manual examination of existing knowledge, and in some cases the Codebook data itself, suggest that they are more likely than not to be potential sequence specific TFs (false negatives), or whether the available evidence instead supports them being true negatives.

In many cases of the unsuccessful proteins that we assessed as unlikely TFs, the main evidence for sequence specific DNA-binding activity is an early individual study without any further evidence. In most of these studies, the DNA binding was observed with Electrophoretic Mobility Shift Assay (EMSA) or SELEX experiments analyzed with low read count sequencing. There are many mechanisms that can lead to false positives in these types of analyzes: **1)** Binding can be indirect, in many cases the proteins have now further evidence for being transcriptional cofactors that either bind DNA indirectly or show only nonspecific binding, such as contacts with DNA backbone; **2)** Proteins can be DNA-binding, but with practically no specificity; **3)** The proteins have been binding to fully or partially single-stranded DNA, which can exist in reactions due to stoichiometric differences of annealed oligomers, or in case of SELEX, if DNA has been amplified with too many PCR cycles and denatured strands have then annealed to wrong counterparts, leading to products with correctly hybridized constant regions and partially single stranded randomized regions – this mechanism can even cause enrichment of aptameric DNA molecules; **4)** SELEX-ligand pool can have lost too much diversity or reflect a PCR-artifact and; **5)** Binding conditions may be too permissible, i.e. capturing DNA-protein interactions with too small affinity to be relevant in physiological conditions.

Based on these factors we decided that in the absence of any other definitive information, isolated sources, such as EMSA, SELEX, or single-locus reporter and ChIP-seq assays do not conclusively show that a protein has sequence specific DNA-binding activity.

All of the SELEX-associated artifacts were observed within Codebook SELEX, but they are likely rare amongst our curated experiments, because we: 1) cross-validated the results from multiple analyses; 2) implemented experimental strategies to reduce their effect (such as an additional double-stranding step carried out with additional primers after each PCR amplification); and 3) because the hundreds of parallel experiments made it possible to detect recurrent artifacts that would have seemed like a real signals if we analyzed only an individual experiment.

For each protein, we show an assessment of previous evidence, a brief summary of the Codebook experiments, a table of assays that it was analyzed with, and a link to the MEX website^12^, that can be used to examine PWMs generated for the protein and their scores across the datatypes.

### (a) C2H2 zinc finger proteins that are unlikely to be sequence specific TFs

Note that “Unconventional C2H2 domain” is defined as a domain that is described as a C2H2 zinc finger (C2H2-zf) domain in some of the databases or literature, but did not meet the recommended HMMer detection threshold of Per-sequence Eval < 0.01 and Per-domain conditional Eval < 0.01, that is used in CisBP (Pfam model: PF00096).

**FAM170A (Unconventional C2H2) is not likely to be a TF.** This protein was included based on it containing an unconventional C2H2-zf domain. A single study predicted that it contains an atypical C2H2-zf domain, localizes to the nucleus and overexpression upregulates other genes^142^. However, based on AlphaFold3 (AF3), the protein does not contain any C2H2-zf domains.

**Codebook experiment summary**: No PBM or ChIP-seq experiments passed quality control. All other experiment types were performed but none of them yielded specific PWMs.

| **FAM170A** | **ChIP** | **GHT** | **HT** | **SMS** | **PBM** | **Lysate** | **eGFP-IVT** | **IVT** |
| --- | --- | --- | --- | --- | --- | --- | --- | --- |
| **Tested?** | TRUE | TRUE | TRUE | TRUE | FALSE | TRUE | TRUE | TRUE |

**KIN (Unconventional C2H2) is not likely to be a TF.** This protein was included based on it containing an unconventional C2H2-zf domain. It is also known as "KIN17 DNA and RNA binding protein". Based on previous EMSA data the protein binds curved DNA associated with erroneous recombination^143^, whereas recent evidence point towards it regulating transcription of ribosomal RNA encoding genes^144^. The C2H2-like domain is a specific “KIN17” C2H2 (IPR056767). There are multiple lines of evidence that this protein binds nucleic acids in the context of DNA metabolism, but no indications pointing to sequence specificity or regulation of transcription^145^. AF3 predicts that the single C2H2 folds properly, but also that it interacts with other regions of the protein, which could interfere with the DNA-binding activity.

**Codebook experiment summary**: No PBM. All other experiment types were performed but none of them yielded specific PWMs.

| **KIN** | **ChIP** | **GHT** | **HT** | **SMS** | **PBM** | **Lysate** | **eGFP-IVT** | **IVT** |
| --- | --- | --- | --- | --- | --- | --- | --- | --- |
| **Tested?** | TRUE | TRUE | TRUE | TRUE | FALSE | TRUE | TRUE | TRUE |

**TRAFD1 (Unconventional C2H2) is not likely to be a TF.** This protein was included based on it containing an unconventional C2H2-zf domain. It is described as negatively regulating IRF3 and NFKB1, but there is no evidence for DNA-binding, and it appears to operate upstream in the signalling cascade^146^. The C2H2-like domain is IPR049439 (TRAFD1/XAF1, zinc finger). AF3 predicts that the protein has 4 canonically folded C2H2-like domains, but they are located immediately adjacent to each other, which is likely incompatible for binding to DNA as a C2H2-zf array.

**Codebook experiment summary**: No PBM or ChIP-seq experiments passed quality control. All experiment types were performed but none of them yielded specific PWMs.

| **TRAFD1** | **ChIP** | **GHT** | **HT** | **SMS** | **PBM** | **Lysate** | **eGFP-IVT** | **IVT** |
| --- | --- | --- | --- | --- | --- | --- | --- | --- |
| **Tested?** | TRUE | TRUE | TRUE | TRUE | FALSE | TRUE | TRUE | TRUE |

**ZNF326 (Unconventional C2H2) is likely an RNA binding protein.** This protein was included based on it containing an unconventional C2H2-zf domain. ZNF326 is a member of the DBIRD complex that regulates splicing by binding to PolII in an RNA-independent manner^147^. The protein domain is of "AKAP95" C2H2 subtype as in AKAP8 proteins. In the AF3 prediction, the C2H2-zf domain is followed immediately by an extra alpha-helix.

**Codebook experiment summary**: No Lysate SELEX or PBM. ChIP-seq did not pass quality control. All experiment types were performed but none of them yielded specific PWMs.

| **ZNF326** | **ChIP** | **GHT** | **HT** | **SMS** | **PBM** | **Lysate** | **eGFP-IVT** | **IVT** |
| --- | --- | --- | --- | --- | --- | --- | --- | --- |
| **Tested?** | TRUE | TRUE | TRUE | TRUE | FALSE | FALSE | TRUE | TRUE |

**ZNF474 (Unconventional C2H2) is not likely to be a TF.** This protein contains a single C2H2-zf domain based on our standard threshold and multiple "C2HC/C3H-type" domains (IPR049899) according to pfam predictions. AF3 predicts that the most C-terminal domain is folded canonically, whereas three other domains have noncanonical features, including a much longer recognition helix and folded domains adjacent to both sides of the C2H2-zf domain. This structure appears incompatible with the established C2H2 DNA binding mode.

**Codebook experiment summary**: No PBM or ChIP-seq experiments passed quality control. None of the other experiments enriched specific PWMs.

| **ZNF474** | **ChIP** | **GHT** | **HT** | **SMS** | **PBM** | **Lysate** | **eGFP-IVT** | **IVT** |
| --- | --- | --- | --- | --- | --- | --- | --- | --- |
| **Tested?** | TRUE | TRUE | TRUE | TRUE | FALSE | TRUE | TRUE | TRUE |

**ANKZF1 (Single C2H2) is not likely to be a TF.** This protein was included based on it containing a potential C2H2-zf. AF3 predicts the correct fold for the putative C2H2-zf, but the protein is known to be an enzyme that cleaves polypeptidyl-tRNAs.

**Codebook experiment summary**: All experiment types were performed, but none of them yielded specific PWMs.

| **ANKZF1** | **ChIP** | **GHT** | **HT** | **SMS** | **PBM** | **Lysate** | **eGFP-IVT** | **IVT** |
| --- | --- | --- | --- | --- | --- | --- | --- | --- |
| **Tested?** | TRUE | TRUE | TRUE | TRUE | TRUE | TRUE | TRUE | TRUE |

**CPXCR1 (Single C2H2) is not likely to be a TF.** This protein was included based on it containing a potential C2H2-zf. Very little is known about this protein except that it is associated with cleft palate. Based on STRING database the protein it interacts with endo- and lysosomal proteins VTA1 (Vesicle Trafficking 1) and CHMP3 (Charged Multivesicular Body Protein 3). AF3 does not predict correct fold for the putative C2H2-zf sequence.

**Codebook experiment summary**: All experiment types were performed but none of them yielded specific PWMs.

| **CPXCR1** | **ChIP** | **GHT** | **HT** | **SMS** | **PBM** | **Lysate** | **eGFP-IVT** | **IVT** |
| --- | --- | --- | --- | --- | --- | --- | --- | --- |
| **Tested?** | TRUE | TRUE | TRUE | TRUE | TRUE | TRUE | TRUE | TRUE |

**DZIP1 (Single C2H2) is not likely to be a TF.** The C2H2-zf domain is folded properly based on AF3 prediction, but this protein is cytosolic, and has a known role in organization of cilia^148^.

**Codebook experiment summary**: All experiment types were performed but none of them yielded specific PWMs.

| **DZIP1** | **ChIP** | **GHT** | **HT** | **SMS** | **PBM** | **Lysate** | **eGFP-IVT** | **IVT** |
| --- | --- | --- | --- | --- | --- | --- | --- | --- |
| **Tested?** | TRUE | TRUE | TRUE | TRUE | TRUE | TRUE | TRUE | TRUE |

**EEA1 (Single C2H2) is not likely to be a TF.** This protein was included based on it containing a potential C2H2-zf domain. The C2H2-zf domain is folded properly based on AF3 prediction. It is known to be a cytosolic and endosomal protein, however, that associates with RAB5A and membranes, and binds phospholipid vesicles^149^.

**Codebook experiment summary**: No ChIP-seq or Lysate SELEX experiments. None of the other experiments enriched specific PWMs.

| **EEA1** | **ChIP** | **GHT** | **HT** | **SMS** | **PBM** | **Lysate** | **eGFP-IVT** | **IVT** |
| --- | --- | --- | --- | --- | --- | --- | --- | --- |
| **Tested?** | FALSE | TRUE | TRUE | TRUE | TRUE | FALSE | TRUE | TRUE |

**L3MBTL4 (Single C2H2) is likely a transcriptional cofactor.** The putative C2H2-zf domain corresponding sequence does not fold into a C2H2-zf domain based on AF3, but is instead a part of globular domain. Its closest paralog L3MBTL3 interacts with histone demethylase KDM1A and RBPJ to repress enhancers^150^. Consistently with its paralog L3MBTL3, it makes protein–protein interactions with chromatin proteins, including RBPJ. An RBPJ-like PWM is enriched in our ChIP-seq data.

**Codebook experiment summary**: No PBM experiment. ChIP-seq experiments show enrichment of RBPJ-like PWMs with “RNTCCCRG” consensus. There is no clear PWM enrichment in other data.

| **L3MBTL4** | **ChIP** | **GHT** | **HT** | **SMS** | **PBM** | **Lysate** | **eGFP-IVT** | **IVT** |
| --- | --- | --- | --- | --- | --- | --- | --- | --- |
| **Tested?** | TRUE | TRUE | TRUE | TRUE | FALSE | TRUE | TRUE | TRUE |

**PRMT3 (Single C2H2) is likely a transcriptional cofactor.** PRMT3 is a protein arginine methyltransferase that is known to modify many proteins, including histone H4^151^. Based on the NMR structure of the mouse homolog, PDB:1WIR^152^, the predicted C2H2-zf sequence is a part of larger folded protein domain and not a real C2H2-zf domain.

**Codebook experiment summary**: No PBM or ChIP-seq experiments passed quality control. None of the other experiments enriched specific PWMs.

| **PRMT3** | **ChIP** | **GHT** | **HT** | **SMS** | **PBM** | **Lysate** | **eGFP-IVT** | **IVT** |
| --- | --- | --- | --- | --- | --- | --- | --- | --- |
| **Tested?** | TRUE | TRUE | TRUE | TRUE | FALSE | TRUE | TRUE | TRUE |

**RBSN (Single C2H2) is not likely to be a TF.** This protein was included based on it containing a potential C2H2-zf. The C2H2-like sequence folds properly based on AF3. It is also known as rabenosyn, and it has a well-established role in membrane trafficking and recycling of endosomes. The Codebook ChIP-seq data produced peaks, suggesting that some proportion of the protein is localized in nucleus and that it may have chromatin associated roles.

**Codebook experiment summary**: No PBM experiment. ChIP-seq reads are enriched in CG-rich sequences, and lysate-based SELEX reads are enriched in NRF1 sites. None of the other experiments enriched specific PWMs.

| **RBSN** | **ChIP** | **GHT** | **HT** | **SMS** | **PBM** | **Lysate** | **eGFP-IVT** | **IVT** |
| --- | --- | --- | --- | --- | --- | --- | --- | --- |
| **Tested?** | TRUE | TRUE | TRUE | TRUE | FALSE | TRUE | TRUE | TRUE |

**ZNF385A and ZNF385B (Single C2H2) are potential RNA-binding proteins**. Many studies have shown that these paralogs function as RNA-binding proteins^153,154^. The SMART database lists three Matrin U1-like C2H2-zf domains for these proteins, and these are supported by AF3 prediction that shows three C2H2-like folded domains, but with an additional alpha-helix following the C2H2-zf domain, and the domains are separated by long gaps, which would be incompatible with the established C2H2-zf DNA-binding mode. SMiLE-seq experiments showed enrichment TTTAA motif for ZNF385B, but there was no support from other analyses^13^.

**Codebook experiment summary**: None of the experiments enriched specific PWMs.

| **ZNF385A** | **ChIP** | **GHT** | **HT** | **SMS** | **PBM** | **Lysate** | **eGFP-IVT** | **IVT** |
| --- | --- | --- | --- | --- | --- | --- | --- | --- |
| **Tested?** | TRUE | TRUE | TRUE | FALSE | FALSE | TRUE | FALSE | TRUE |
| **ZNF385B** | **ChIP** | **GHT** | **HT** | **SMS** | **PBM** | **Lysate** | **eGFP-IVT** | **IVT** |
| **Tested?** | TRUE | TRUE | TRUE | TRUE | FALSE | TRUE | TRUE | TRUE |

**ZNF804A (Single C2H2) is not likely to be a TF.** This protein was included based on it containing a potential C2H2-zf. Protein-protein interactions show that it interacts with many alternative splicing associated RNA-binding proteins^155^. The protein has been also shown to bind to STAT2 and it potentially affects its transport from cytoplasm to nucleus as a part of interferon response^156^.

**Codebook experiment summary**: No PBM or ChIP-seq experiments passed quality control. None of the other experiments enriched specific PWMs.

| **ZNF804A** | **ChIP** | **GHT** | **HT** | **SMS** | **PBM** | **Lysate** | **eGFP-IVT** | **IVT** |
| --- | --- | --- | --- | --- | --- | --- | --- | --- |
| **Tested?** | TRUE | TRUE | TRUE | TRUE | FALSE | TRUE | TRUE | TRUE |

**ZMAT4 (C2H2 with noncanonical gaps) is not likely to be a TF.** This protein has been annotated to have four (4) potential C2H2-zf domains. The C2H2-zf domains are all “Matrin U1” domains (IPR003604), however, which are associated with RNA binding, and have an extra alpha helix adjacent to the C2H2-zf domain in AF3 predictions. ZMAT4 interacts with RNA-binding- and ubiquitinoylation-associated proteins, according to the STRING database.

**Codebook experiment summary**: No Lysate SELEX or PBM experiments. ChIP-seq did not pass quality control. None of the other experiments enriched specific PWMs.

| **ZMAT4** | **ChIP** | **GHT** | **HT** | **SMS** | **PBM** | **Lysate** | **eGFP-IVT** | **IVT** |
| --- | --- | --- | --- | --- | --- | --- | --- | --- |
| **Tested?** | TRUE | TRUE | TRUE | TRUE | FALSE | FALSE | TRUE | TRUE |

**ZNF207 (C2H2 with noncanonical gaps) is not likely to be a TF.** This protein has been annotated to have two (2) potential (and overlapping) C2H2-zf domains. AF3 predicts that the overlapping C2H2-zfs do not fold into a C2H2-zf domain, but instead into a completely different protein fold. ZNF207 also has also a very well characterized role as a kinetochore- and microtubule-binding protein^157,158^.

**Codebook experiment summary**: No eGFP-IVT SELEX or PBM experiments. All other types of experiments were performed and none enriched specific PWMs.

| **ZNF207** | **ChIP** | **GHT** | **HT** | **SMS** | **PBM** | **Lysate** | **eGFP-IVT** | **IVT** |
| --- | --- | --- | --- | --- | --- | --- | --- | --- |
| **Tested?** | TRUE | TRUE | TRUE | TRUE | FALSE | TRUE | FALSE | TRUE |

**ZNF318 (C2H2 with noncanonical gaps) is not likely to be a TF.** This protein has been annotated to have two (2) potential C2H2-zf domains, which are also Matrin U1-type zinc finger domains^159^ (IPR003604), typically associated with RNA binding. Based on AF3, these domains do not fold into individual C2H2-zf domains, but instead, fold together with additional structural features.

**Codebook experiment summary**: No PBM experiment. None of the other experiments enriched specific PWMs.

| **ZNF318** | **ChIP** | **GHT** | **HT** | **SMS** | **PBM** | **Lysate** | **eGFP-IVT** | **IVT** |
| --- | --- | --- | --- | --- | --- | --- | --- | --- |
| **Tested?** | TRUE | TRUE | TRUE | TRUE | FALSE | TRUE | TRUE | TRUE |

**ZNF385C (C2H2 with noncanonical gaps) is not likely to be a TF.** This protein has been annotated to have two (2) potential C2H2-zf domains. The SMART-database lists it as having four Matrin U1 subtype C2H2-zf domains (IPR003604), however, which are associated with RNA binding. The C2H2-like domains do fold into the expected structure, but they have an extra alpha helix on AF3 predictions, which would be inconsistent with the typical C2H2-zf DNA-binding mode. **Codebook experiment summary**: No PBM or ChIP-seq experiments passed quality control. None of the other experiments enriched specific PWMs.

| **ZNF385C** | **ChIP** | **GHT** | **HT** | **SMS** | **PBM** | **Lysate** | **eGFP-IVT** | **IVT** |
| --- | --- | --- | --- | --- | --- | --- | --- | --- |
| **Tested?** | TRUE | TRUE | TRUE | TRUE | FALSE | TRUE | TRUE | TRUE |

**ZNF598 (C2H2 with noncanonical gaps) is not likely to be a TF.** This protein has been annotated as having five (5) potential C2H2-zf domains. A subset fold in the expected manner on AF3. This protein is a known ubiquitin ligase, however, which functions in translation quality control.

**Codebook experiment summary**: No PBM or ChIP-seq experiments passed quality control. None of the other experiments enriched specific PWMs.

| **ZNF598** | **ChIP** | **GHT** | **HT** | **SMS** | **PBM** | **Lysate** | **eGFP-IVT** | **IVT** |
| --- | --- | --- | --- | --- | --- | --- | --- | --- |
| **Tested?** | TRUE | TRUE | TRUE | TRUE | FALSE | TRUE | TRUE | TRUE |

**ZUP1 (C2H2 with noncanonical gaps) is a potential RNA-binding protein**. This protein has two potential C2H2-zf domains, which fold as expected on AF3. It has been reported to use its zinc fingers as a viral RNA-binding protein that functions as a part of the MAVS complex, which activates IRF3 and NFKB TFs^160^. The protein also has also well-established role as a de-ubiquitinase functioning in DNA damage response^160^.

**Codebook experiment summary**: No eGFP-IVT SELEX or PBM experiments. ChIP-seq did not pass quality control. None of the other experiments enriched specific PWMs.

| **ZUP1** | **ChIP** | **GHT** | **HT** | **SMS** | **PBM** | **Lysate** | **eGFP-IVT** | **IVT** |
| --- | --- | --- | --- | --- | --- | --- | --- | --- |
| **Tested?** | TRUE | TRUE | TRUE | TRUE | FALSE | TRUE | FALSE | TRUE |

### (b) C2H2 zinc finger proteins that are potential sequence specific TFs

**AKAP8 and AKAP8L (Unconventional C2H2) are potential TFs.** These proteins were included based on containing an unconventional C2H2-zf domain. AKAP8 is also known as AKAP95 and is associated with many different roles. In a previous study, AKAP8/AKAP/95 bound to G/C-rich DNA according to SELEX^161^ and associates with rDNA promoters according to Interpro. Their C2H2-zf domain is of type “AKAP95-type 2 domain” that is associated with RNA binding. Other lines of evidence, however point towards them being RNA-binding proteins are that they are associated with alternative splicing^162^ or participating in subcellular targeting of cyclic AMP-dependent protein kinase A^163^.

**Codebook experiment summary**: All experiment types were performed but none of them yielded specific PWMs.

| **AKAP8** | **ChIP** | **GHT** | **HT** | **SMS** | **PBM** | **Lysate** | **eGFP-IVT** | **IVT** |
| --- | --- | --- | --- | --- | --- | --- | --- | --- |
| **Tested?** | TRUE | TRUE | TRUE | TRUE | TRUE | TRUE | TRUE | TRUE |
| **AKAP8L** | **ChIP** | **GHT** | **HT** | **SMS** | **PBM** | **Lysate** | **eGFP-IVT** | **IVT** |
| **Tested?** | TRUE | TRUE | TRUE | TRUE | TRUE | TRUE | TRUE | TRUE |

**ZNF706 (Unconventional C2H2) is a potential TF.** This protein was included based on containing an unconventional C2H2-zf domain. This tiny protein (76AA) works in controlling stem cell state via regulating KLF4 expression^164^. The study establishing this role did not decipher the mechanism, however.

**Codebook experiment summary**: No PBM or ChIP-seq experiment. Other experiment trypes were performed but without specific PWM enrichment.

| **ZNF706** | **ChIP** | **GHT** | **HT** | **SMS** | **PBM** | **Lysate** | **eGFP-IVT** | **IVT** |
| --- | --- | --- | --- | --- | --- | --- | --- | --- |
| **Tested?** | FALSE | TRUE | TRUE | TRUE | FALSE | TRUE | TRUE | TRUE |

**ZNF750 (Unconventional C2H2) is likely a transcriptional cofactor and potentially a TF.** The main source of evidence for the role of ZNF750 as a TF is that it has a potential C2H2-zf domain, which is classified as a specific subtype “Zinc finger protein 750-like” (IPR039064). AF3 does not predict any C2H2-zf folded regions within the protein, however. A ChIP-seq study concluded that it binds “NNSCYNNRGGSW” motif, which is indistinguishable from target specificities of TFAP2 proteins. The same study has also mass-spectrometry data that shows that this protein interacts strongly with TFAP2A and thus the binding is likely indirect^165^. The same study also indicates that it interacts with KLF4, and consistent with this observation, an earlier study shows that it may Influence KLF4 expression^166^.

**Codebook experiment summary**: No PBM experiment. All experiment types were performed but none of them yielded specific PWMs.

| **ZNF750** | **ChIP** | **GHT** | **HT** | **SMS** | **PBM** | **Lysate** | **eGFP-IVT** | **IVT** |
| --- | --- | --- | --- | --- | --- | --- | --- | --- |
| **Tested?** | TRUE | TRUE | TRUE | TRUE | FALSE | TRUE | TRUE | TRUE |

**CCDC17 (Single C2H2) is likely a transcriptional cofactor and a potential TF.** This protein was included based on it containing a potential C2H2-zf domain. Very little is known about this protein except that it makes strong protein–protein interactions with RNA PolII and cohesin components. The C2H2-zf domain is folded properly based on AF3 prediction.

**Codebook experiment summary**: No ChIP-seq experiment. Other experiments were performed but without specific PWM enrichment.

| **CCDC17** | **ChIP** | **GHT** | **HT** | **SMS** | **PBM** | **Lysate** | **eGFP-IVT** | **IVT** |
| --- | --- | --- | --- | --- | --- | --- | --- | --- |
| **Tested?** | FALSE | TRUE | TRUE | TRUE | TRUE | TRUE | TRUE | TRUE |

**ZMAT1 (Single C2H2) is a potential TF.** This protein was included based on it containing a potential C2H2-zf. Little is known about this protein; It interacts mostly with RBPs and has been suggested to function either as RBP or TF. On AF3, the N-terminal sequence folds into a C2H2-zf fold that is followed directly by a long alpha-helix.

**Codebook experiment summary**: No SMS or PBM experiments. ChIP-seq did not pass quality control. All other experiment types were performed but none of them yielded specific PWMs.

| **ZMAT1** | **ChIP** | **GHT** | **HT** | **SMS** | **PBM** | **Lysate** | **eGFP-IVT** | **IVT** |
| --- | --- | --- | --- | --- | --- | --- | --- | --- |
| **Tested?** | TRUE | TRUE | TRUE | FALSE | FALSE | TRUE | TRUE | TRUE |

**ZNF365 (Single C2H2) is a potential TF.** The C2H2-zf domain in this protein mediates interaction between it and a complex that contains PARP1 and MRE11^167^. ZNF365 contains a Ku C terminal like domain that is thought to be involved in DNA-damage repair pathways. AF3 predicts that the predicted C2H2-zf domain folds properly.

**Codebook experiment summary**: No ChIP-seq or PBM experiments. Other experiments were performed but without specific PWM enrichment.

| **ZNF365** | **ChIP** | **GHT** | **HT** | **SMS** | **PBM** | **Lysate** | **eGFP-IVT** | **IVT** |
| --- | --- | --- | --- | --- | --- | --- | --- | --- |
| **Tested?** | FALSE | TRUE | TRUE | TRUE | FALSE | TRUE | TRUE | TRUE |

**ZNF428 (Single C2H2) is a potential TF.** This protein was included based on it containing a potential C2H2-zf. Little is known about this protein, but AF3 predicts that the C2H2-zf is folded properly, and that it is the only folded domain in the protein.

**Codebook experiment summary**: No PBM experiment. eGFP-IVT based HT-SELEX enriched specific “CCGTA” motifs but the enrichment was weak and none of the other experiments supported the result.

| **ZNF428** | **ChIP** | **GHT** | **HT** | **SMS** | **PBM** | **Lysate** | **eGFP-IVT** | **IVT** |
| --- | --- | --- | --- | --- | --- | --- | --- | --- |
| **Tested?** | TRUE | TRUE | TRUE | TRUE | FALSE | TRUE | TRUE | TRUE |

**ZNF608 (Single C2H2) is a potential TF.** This protein was included based on it containing a potential C2H2-zf. Little is known about this protein. AF3 predicts that the C2H2-zf is folded properly.

**Codebook experiment summary**: No PBM experiments. All other experiment types were performed but without specific PWM enrichment.

| **ZNF608** | **ChIP** | **GHT** | **HT** | **SMS** | **PBM** | **Lysate** | **eGFP-IVT** | **IVT** |
| --- | --- | --- | --- | --- | --- | --- | --- | --- |
| **Tested?** | TRUE | TRUE | TRUE | TRUE | FALSE | TRUE | TRUE | TRUE |

**ZNF609 (Single C2H2) is a potential TF.** This protein was included based on it containing a potential C2H2-zf. Little is known about this protein. AF3 predicts that the C2H2-zf is folded properly. The corresponding gene makes a circ-RNA that has recently been studied extensively.

**Codebook experiment summary**: No PBM or ChIP-seq experiments passed quality control. All experiment types were performed but none of them yielded specific PWMs.

| **ZNF609** | **ChIP** | **GHT** | **HT** | **SMS** | **PBM** | **Lysate** | **eGFP-IVT** | **IVT** |
| --- | --- | --- | --- | --- | --- | --- | --- | --- |
| **Tested?** | TRUE | TRUE | TRUE | TRUE | FALSE | TRUE | TRUE | TRUE |

**KAT7 (Single C2H2) is a transcriptional cofactor and potentially a TF**. This protein was included based on it containing a potential C2H2-zf domain. However, the protein is a well-established H3K14 acetyltransferase. AF3 also predicts that its putative C2H2-zf sequence does not fold into a C2H2-zf domain. There is an external ChIP-seq-derived CG-rich PWM, which is potentially a false positive derived from indirect binding.

**Codebook experiment summary**: No PBM experiment. ChIP-seq enriches weakly CG-rich PWMs but there is no PWM enrichment in other datasets.

| **KAT7** | **ChIP** | **GHT** | **HT** | **SMS** | **PBM** | **Lysate** | **eGFP-IVT** | **IVT** |
| --- | --- | --- | --- | --- | --- | --- | --- | --- |
| **Tested?** | TRUE | TRUE | TRUE | TRUE | FALSE | TRUE | TRUE | TRUE |

**PEG3 (C2H2 with noncanonical gaps) is a potential transcriptional factor.** This protein has been annotated to have 11 potential C2H2-zf domains. Previous studies have analyzed the target specificity of PEG3 with ChIP-seq and EMSA^168^, and ChIP-qPCR and EMSA^169^. PWM construction in these studies involved non-standard data analysis methods, and the resulting PWMs are in disagreement. AF3 predicts that some of the putative 13 C2H2-zf domains fold correctly.

**Codebook experiment summary**: No PBM experiment. All other experiment types were performed but without specific PWM enrichment.

| **PEG3** | **ChIP** | **GHT** | **HT** | **SMS** | **PBM** | **Lysate** | **eGFP-IVT** | **IVT** |
| --- | --- | --- | --- | --- | --- | --- | --- | --- |
| **Tested?** | TRUE | TRUE | TRUE | TRUE | FALSE | TRUE | TRUE | TRUE |

**PRDM8 (C2H2 with noncanonical gaps) is a transcriptional cofactor and potentially a TF.** This protein has been annotated to have 2 potential C2H2-zf domains. The same study that established PRDM8 as a histone methyltransferase (H3K9me3) included ChIP-seq, which did not result in a specific PWMs^170^. AF3 predicts that both C2H2-zf domains fold correctly, but the first one has a very long recognition helix and the second one is in the extreme C-terminus of the protein.

**Codebook experiment summary**: No PBM experiment. All other experiment types were performed but without specific PWM enrichment.

| **PRDM8** | **ChIP** | **GHT** | **HT** | **SMS** | **PBM** | **Lysate** | **eGFP-IVT** | **IVT** |
| --- | --- | --- | --- | --- | --- | --- | --- | --- |
| **Tested?** | TRUE | TRUE | TRUE | TRUE | FALSE | TRUE | TRUE | TRUE |

**ZFPM2 (C2H2 with noncanonical gaps) is a transcriptional cofactor and potentially a TF.** This protein has been annotated to have 3 potential C2H2-zf domains. ZFPM2 is also known as Friend Of GATA 2 (FOG2), and FOGs are known to use some of their zinc fingers to interact with the GATA proteins and other TFs^171^. It is still unclear whether these proteins have intrinsic target specificity. Its paralog ZFPM1 enriched a specific GATA-consensus PWM. The protein used in that experiment was produced in wheat germ extract system and it could be co-precipitating a wheat GATA protein. AF3 predicts that the protein has more than three domains with C2H2-like fold.

**Codebook experiment summary**: No PBM experiment. All other experiment types were performed but without specific PWM enrichment.

| **ZFPM2** | **ChIP** | **GHT** | **HT** | **SMS** | **PBM** | **Lysate** | **eGFP-IVT** | **IVT** |
| --- | --- | --- | --- | --- | --- | --- | --- | --- |
| **Tested?** | TRUE | TRUE | TRUE | TRUE | FALSE | TRUE | TRUE | TRUE |

**ZNF644 (C2H2 with noncanonical gaps) is a potential TF.** This protein has been annotated to have 2 potential C2H2-zf domains. The C2H2-zf domains fold correctly in AF3 predictions. Very little is known about the protein.

**Codebook experiment summary**: No PBM experiment. ChIP-seq reads are enriched in YY1 sites. None of the other experiment types yielded specific PWMs.

| **ZNF644** | **ChIP** | **GHT** | **HT** | **SMS** | **PBM** | **Lysate** | **eGFP-IVT** | **IVT** |
| --- | --- | --- | --- | --- | --- | --- | --- | --- |
| **Tested?** | TRUE | TRUE | TRUE | TRUE | FALSE | TRUE | TRUE | TRUE |

### (c) Unsuccessful proteins with canonical C2H2 arrays (potential TFs)

The following proteins contain standard C2H2-zf arrays. We did not annotate them on a case-by-case basis and they are all potential TFs. SMiLE-seq experiments showed motif enrichment for JAZF1 and ZBTB46, but there was no support from other analyses ^13^.

| TF name | #C2H2 | **ChIP** | **GHT** | **HT** | **SMS** | **PBM** | **Lysate** | **eGFP-IVT** | **IVT** |
| --- | --- | --- | --- | --- | --- | --- | --- | --- | --- |
| AC008770 | 13 | TRUE | TRUE | TRUE | TRUE | TRUE | FALSE | TRUE | TRUE |
| CHAMP1 | 5 | TRUE | TRUE | TRUE | TRUE | TRUE | TRUE | TRUE | TRUE |
| JAZF1 | 2 | TRUE | TRUE | TRUE | TRUE | FALSE | TRUE | TRUE | TRUE |
| PRDM2 | 5 | TRUE | TRUE | TRUE | TRUE | FALSE | TRUE | TRUE | TRUE |
| ZBTB46 | 3 | TRUE | TRUE | TRUE | TRUE | FALSE | TRUE | TRUE | TRUE |
| ZNF230 | 8 | TRUE | TRUE | TRUE | TRUE | FALSE | TRUE | TRUE | TRUE |
| ZNF280B | 6 | TRUE | TRUE | TRUE | TRUE | FALSE | TRUE | TRUE | TRUE |
| ZNF280D | 9 | TRUE | TRUE | TRUE | TRUE | FALSE | TRUE | TRUE | TRUE |
| ZNF446 | 3 | TRUE | TRUE | TRUE | TRUE | FALSE | TRUE | TRUE | TRUE |
| ZNF469 | 3 | TRUE | TRUE | TRUE | TRUE | FALSE | FALSE | TRUE | TRUE |
| ZNF579 | 9 | TRUE | TRUE | TRUE | TRUE | FALSE | FALSE | FALSE | TRUE |
| ZNF592 | 5 | TRUE | TRUE | TRUE | TRUE | FALSE | TRUE | TRUE | TRUE |
| ZNF630 | 13 | TRUE | TRUE | TRUE | TRUE | FALSE | TRUE | TRUE | TRUE |
| ZNF639 | 8 | TRUE | TRUE | TRUE | TRUE | FALSE | TRUE | TRUE | TRUE |
| ZNF654 | 3 | TRUE | TRUE | TRUE | TRUE | FALSE | TRUE | TRUE | TRUE |
| ZNF687 | 10 | TRUE | TRUE | TRUE | TRUE | FALSE | TRUE | TRUE | TRUE |
| ZNF688 | 2 | TRUE | TRUE | TRUE | TRUE | FALSE | TRUE | TRUE | TRUE |
| ZNF717 | 21 | TRUE | TRUE | TRUE | TRUE | FALSE | TRUE | TRUE | TRUE |
| ZNF763 | 6 | TRUE | TRUE | TRUE | TRUE | FALSE | TRUE | TRUE | TRUE |
| ZNF781 | 4 | TRUE | TRUE | TRUE | TRUE | FALSE | TRUE | TRUE | TRUE |
| ZNF844 | 7 | TRUE | TRUE | TRUE | TRUE | FALSE | TRUE | TRUE | TRUE |
| ZNF91 | 33 | FALSE | TRUE | TRUE | TRUE | FALSE | FALSE | FALSE | TRUE |
| ZSCAN18 | 2 | TRUE | TRUE | TRUE | TRUE | FALSE | TRUE | FALSE | TRUE |

### (d) Other known-DBD-containing proteins that are unlikely to be sequence specific TFs

**ARID2 (ARID/BRIGHT; RFX) is likely a low specificity transcriptional cofactor**. This protein has an ARID/BRIGHT DBD. It participates as a component in some forms of the SWI/SNF remodeler^172^. In the structures existing for this protein^173^, it either does not contact DNA (PDB:7VDV) but instead binds the histone core, or makes very minor backbone contacts (PDB:7Y8R). The structures contain only a part of this protein, and there is no clear evidence that would rule out sequence specific DNA binding. Among ARID family proteins, however, only ARID3 and ARID5 subgroups are known to possess strong sequence specificity.

**Codebook experiment summary**: No SMS experiment. The protein showed strong enrichment for CTCF sites in ChIP-seq. All other experiment types were performed but without specific PWM enrichment.

| **ARID2** | **ChIP** | **GHT** | **HT** | **SMS** | **PBM** | **Lysate** | **eGFP-IVT** | **IVT** |
| --- | --- | --- | --- | --- | --- | --- | --- | --- |
| **Tested?** | TRUE | TRUE | TRUE | FALSE | TRUE | TRUE | TRUE | TRUE |

**AHDC1 (AT hook) is likely a low specificity transcriptional cofactor**. This protein has an AT-hook DNA-binding peptide motif, which would suggest interaction with DNA, but on the basis of the fact that it has many protein-protein interactions with other TFs (including dozens of C2H2-zf proteins), it could be a cofactor.

**Codebook experiment summary**: Only ChIP-seq and Lysate-based SELEX experiments. ChIP-seq yielded motif, but is not supported by other experiments.

| **AHDC1** | **ChIP** | **GHT** | **HT** | **SMS** | **PBM** | **Lysate** | **eGFP-IVT** | **IVT** |
| --- | --- | --- | --- | --- | --- | --- | --- | --- |
| **Tested?** | TRUE | TRUE | TRUE | FALSE | FALSE | TRUE | FALSE | FALSE |

**AKNA (AT hook) is not likely to be a TF**. This protein has an AT-hook DNA-binding peptide motif. Has a well-established role in centrosome formation. It was included in the study because it has been suggested to moonlight as TF through binding AT-rich promoters of CD40 and CD40L and coordinate their expression^174^.

**Codebook experiment summary**: All experiment types were performed but none of them yielded specific PWMs.

| **AKNA** | **ChIP** | **GHT** | **HT** | **SMS** | **PBM** | **Lysate** | **eGFP-IVT** | **IVT** |
| --- | --- | --- | --- | --- | --- | --- | --- | --- |
| **Tested?** | TRUE | TRUE | TRUE | TRUE | TRUE | TRUE | TRUE | TRUE |

**CBX2 (AT hook) is likely a low specificity transcriptional cofactor.** This protein has an AT-hook DNA-binding peptide motif. CBX2 functions in the polycomb repressive complex, binding trimethylated H3K27 with its chromodomain, and the AT-hook may contribute into this interaction^175^.

**Codebook experiment summary**: No IVT SELEX or PBM experiments. Other experiment types were performed but without specific PWM enrichment.

| **CBX2** | **ChIP** | **GHT** | **HT** | **SMS** | **PBM** | **Lysate** | **eGFP-IVT** | **IVT** |
| --- | --- | --- | --- | --- | --- | --- | --- | --- |
| **Tested?** | TRUE | TRUE | TRUE | TRUE | FALSE | TRUE | TRUE | FALSE |

**DOT1L (AT hook) is likely a low specificity transcriptional cofactor**. This protein has an AT-hook DNA-binding peptide motif**.** It is known to be a histone methyltransferase. The putative AT-hook may contribute to DNA interactions but is unlikely to define target locations. The protein binds the histone core in PDB:6J99 and makes only very minor contacts with DNA backbone^176^.

**Codebook experiment summary**: Only ChIP-seq and Lysate SELEX experiments. Other experiment types were performed but without specific PWM enrichment.

| **DOT1L** | **ChIP** | **GHT** | **HT** | **SMS** | **PBM** | **Lysate** | **eGFP-IVT** | **IVT** |
| --- | --- | --- | --- | --- | --- | --- | --- | --- |
| **Tested?** | TRUE | TRUE | TRUE | FALSE | FALSE | TRUE | FALSE | FALSE |

**PHF20 (AT hook) is likely a low specificity transcriptional cofactor**. This protein has an AT-hook DNA-binding peptide motif. PHF20 is known to bind di-methylated H3K4me2 and to function as a part of "nonspecific lethal" protein complex that regulates gene expression through histone acetyltransferase activity^177^.

**Codebook experiment summary**: It has only ChIP-seq data, with no specific enrichment.

| **PHF20** | **ChIP** | **GHT** | **HT** | **SMS** | **PBM** | **Lysate** | **eGFP-IVT** | **IVT** |
| --- | --- | --- | --- | --- | --- | --- | --- | --- |
| **Tested?** | TRUE | FALSE | FALSE | FALSE | FALSE | FALSE | FALSE | FALSE |

**PRR12 (AT hook) is likely a low specificity transcriptional cofactor**. This protein has an AT-hook DNA-binding peptide motif. It has been recently shown to interact with cohesin complex through NIPBL^178^. Based on protein–protein interactions, it also associates with TFs and cofactors, consisted with a role as a transcriptional cofactor.

**Codebook experiment summary**: Only ChIP-seq and Lysate SELEX experiments. ChIP-seq reads are enriched posterior homeodomain sites. No clear enrichment in Lysate SELEX experiments.

| **PRR12** | **ChIP** | **GHT** | **HT** | **SMS** | **PBM** | **Lysate** | **eGFP-IVT** | **IVT** |
| --- | --- | --- | --- | --- | --- | --- | --- | --- |
| **Tested?** | TRUE | TRUE | TRUE | FALSE | FALSE | TRUE | FALSE | FALSE |

**SCML4 (AT hook) is likely a low specificity transcriptional cofactor**. This protein has an AT-hook DNA-binding peptide motif. Very little is known about the protein. It associates with TFs and cofactors based on protein–protein interactions.

**Codebook experiment summary**: No SMS experiment. All other types of experiments were tested, with no clear motifs.

| **SCML4** | **ChIP** | **GHT** | **HT** | **SMS** | **PBM** | **Lysate** | **eGFP-IVT** | **IVT** |
| --- | --- | --- | --- | --- | --- | --- | --- | --- |
| **Tested?** | TRUE | TRUE | TRUE | FALSE | TRUE | TRUE | TRUE | TRUE |

**SGSM2 (BED ZF) is unlikely to be a TF**. The protein was included because it was predicted to contain a ZBED domain. However, AF3 predicts that the associated sequence does not fold into a ZBED domain but is instead a part of larger folded protein domain. So, this protein is not a ZBED. The protein has GTPase activity and is thought to function in membrane trafficking.

**Codebook experiment summary**: There is an enrichment for CTCF sites in ChIP-seq. Other experiment types were performed but without specific PWM enrichment.

| **SGSM2** | **ChIP** | **GHT** | **HT** | **SMS** | **PBM** | **Lysate** | **eGFP-IVT** | **IVT** |
| --- | --- | --- | --- | --- | --- | --- | --- | --- |
| **Tested?** | TRUE | TRUE | TRUE | TRUE | TRUE | TRUE | TRUE | TRUE |

**NCOA1, NCOA2 and NCOA3 (bHLH) are likely non-DNA-binding transcriptional cofactors.** The main source of evidence for their role as TFs is that they have an apparent bHLH domain. There is no previous evidence of them interacting with DNA directly, however; They are best known as coactivators (for which they are named). The HLH domain is known to function as a protein-protein interaction interphase for other TFs.

**Codebook experiment summary**: The NCOA family proteins were systematically unsuccessful in Codebook experiments. Protein showed enrichment for CG-rich sites in ChIP-seq. Other experiment types were performed but without specific PWM enrichment.

| **NCOA1** | **ChIP** | **GHT** | **HT** | **SMS** | **PBM** | **Lysate** | **eGFP-IVT** | **IVT** |
| --- | --- | --- | --- | --- | --- | --- | --- | --- |
| **Tested?** | TRUE | TRUE | TRUE | FALSE | TRUE | TRUE | TRUE | TRUE |
| **NCOA2** | **ChIP** | **GHT** | **HT** | **SMS** | **PBM** | **Lysate** | **eGFP-IVT** | **IVT** |
| **Tested?** | TRUE | TRUE | TRUE | TRUE | TRUE | TRUE | TRUE | TRUE |
| **NCOA3** | **ChIP** | **GHT** | **HT** | **SMS** | **PBM** | **Lysate** | **eGFP-IVT** | **IVT** |
| **Tested?** | TRUE | TRUE | TRUE | FALSE | TRUE | TRUE | TRUE | TRUE |

**ZC3H8 (CCCH ZF) is an RNA-binding protein.** A recombinant protein. EMSA experiments on the protein’s mouse ortholog has indicated that it binds DNA specifically^179^. However, the protein has been shown to be a sequence specific RNA-binding protein that binds “GCUUGY” consensus based on HTR-SELEX^180^, and overall, the CCCH-zf domains are known to be RNA-binding proteins. RNA-binding proteins can often also bind single-stranded DNA.

**Codebook experiment summary**: No ChIP-seq experiment. All other experiment types were performed but without specific PWM enrichment.

| **ZC3H8** | **ChIP** | **GHT** | **HT** | **SMS** | **PBM** | **Lysate** | **eGFP-IVT** | **IVT** |
| --- | --- | --- | --- | --- | --- | --- | --- | --- |
| **Tested?** | FALSE | TRUE | TRUE | TRUE | TRUE | TRUE | TRUE | TRUE |

**ZGPAT (CCCH ZF) is likely a transcriptional cofactor and a potential RNA-binding protein**. This protein has a CCCH-zf domain, which is best known for binding RNA. In published analyses, it binds “RRRGGAGGAGA” consensus based on SELEX performed with purified protein, and cell extract-based EMSA. As with ZC3H8 above, we note that RNA-binding proteins can often bind single-stranded DNA. The same study also shows mass spectrometry evidence that it binds NuRD complex^181^.

**Codebook experiment summary**: All experiment types were performed but none of them yielded specific PWMs.

| **ZGPAT** | **ChIP** | **GHT** | **HT** | **SMS** | **PBM** | **Lysate** | **eGFP-IVT** | **IVT** |
| --- | --- | --- | --- | --- | --- | --- | --- | --- |
| **Tested?** | TRUE | TRUE | TRUE | TRUE | TRUE | TRUE | TRUE | TRUE |

**HMGN3 (HMG/Sox) is likely a low specificity DNA-binding cofactor**. This protein has an HMG DBD of a type that is typically thought to be non-specific DNA-binding domain. HMGN3 binds nucleosomal DNA^182^.

**Codebook experiment summary**: ChIP-seq did not pass quality control. Other experiment types were performed but without specific PWM enrichment.

| **HMGN3** | **ChIP** | **GHT** | **HT** | **SMS** | **PBM** | **Lysate** | **eGFP-IVT** | **IVT** |
| --- | --- | --- | --- | --- | --- | --- | --- | --- |
| **Tested?** | TRUE | TRUE | TRUE | TRUE | TRUE | TRUE | TRUE | TRUE |

**POU5F2 (Homeodomain; POU) is not likely to be a TF.** All of the Codebook experiments were unsuccessful, whereas its closest paralog POU5F1 (that was run as a positive control) was successful in all methods except for ChIP-seq. Furthermore, there is no clear evidence of sequence specific DNA-binding activity even though its paralog, POU5F1, is one of the best characterized human TFs.

When compared against the structural data for POU5F1, all base contacting residues in POU5F2 appear to be intact. The protein has less basic intrinsically disordered spacer between the POU and the homeodomain, and the homeodomain has a shorter DNA recognition helix that misses several K and R residues that make electrostatic interactions with DNA backbone in the POU5F1 structure. Taken together, the evidence suggests that POU5F2 has lost its sequence specific DNA-binding capability.

**Codebook experiment summary**: This protein showed strong enrichment for posterior homeodomain sites in ChIP-seq, which could be due to indirect binding, but there was no enrichment for expected POU class target sites. None of the other experiment types enriched specific PWMs.

| **POU5F2** | **ChIP** | **GHT** | **HT** | **SMS** | **PBM** | **Lysate** | **eGFP-IVT** | **IVT** |
| --- | --- | --- | --- | --- | --- | --- | --- | --- |
| **Tested?** | TRUE | TRUE | TRUE | TRUE | TRUE | TRUE | TRUE | TRUE |

**BAZ2A (MBD) is likely a low specificity DNA-binding cofactor**. This protein encodes a subtype of the MBD domain, called a TAM domain, that can bind unmethylated DNA in a non-specific manner, as well as a potential AT-hook DNA-binding peptide motif. It binds DNA backbone (PDB:7FHJ, 7MWL, and 7MWH)^183^ and also contacts acetylated histones.

**Codebook experiment summary**: Only ChIP-seq and Lysate SELEX experiments. ChIP-seq did not pass quality control. None of the other experiment types yielded specific PWMs.

| **BAZ2A** | **ChIP** | **GHT** | **HT** | **SMS** | **PBM** | **Lysate** | **eGFP-IVT** | **IVT** |
| --- | --- | --- | --- | --- | --- | --- | --- | --- |
| **Tested?** | TRUE | TRUE | TRUE | FALSE | FALSE | TRUE | FALSE | FALSE |

**BAZ2B (MBD; AT hook) is likely a low specificity DNA-binding cofactor**. This is a close paralog of BAZ2A (above).

**Codebook experiment summary**: Only ChIP-seq and Lysate SELEX experiments, with no specific PWM enrichment.

| **BAZ2B** | **ChIP** | **GHT** | **HT** | **SMS** | **PBM** | **Lysate** | **eGFP-IVT** | **IVT** |
| --- | --- | --- | --- | --- | --- | --- | --- | --- |
| **Tested?** | TRUE | TRUE | TRUE | FALSE | FALSE | TRUE | FALSE | FALSE |

**PIN1 (MBD) is not likely to be a TF**. A specific isoform of this protein contains a putative MBD domain; however, the isoform is not found in other animals and may be spurious. In addition, the protein is a peptidyl-prolyl cis/trans isomerase that is not associated with chromatin.

**Codebook experiment summary**: No SMS experiment. All other experiment types were tested without a clear PWM enrichment. None of the *in vitro* experiments were performed with methylated DNA.

| **PIN1** | **ChIP** | **GHT** | **HT** | **SMS** | **PBM** | **Lysate** | **eGFP-IVT** | **IVT** |
| --- | --- | --- | --- | --- | --- | --- | --- | --- |
| **Tested?** | TRUE | TRUE | TRUE | FALSE | TRUE | TRUE | TRUE | TRUE |

**RFX8 (RFX) is not likely to be a TF.** This protein encodes an RFX domain, however based on structural data for paralogs, the RFX domain is truncated – it is missing a region that makes backbone contacts with DNA, and thus has likely lost its DNA-binding functionality.

**Codebook experiment summary:** ChIP-seq did not pass quality control. All experiment types were performed but none of them yielded specific PWMs.

| **RFX8** | **ChIP** | **GHT** | **HT** | **SMS** | **PBM** | **Lysate** | **eGFP-IVT** | **IVT** |
| --- | --- | --- | --- | --- | --- | --- | --- | --- |
| **Tested?** | TRUE | TRUE | TRUE | TRUE | TRUE | TRUE | TRUE | TRUE |

**THAP (THAP finger) proteins appear to be relatively low-affinity DNA binding TFs (and possibly low-specificity).** The nine THAP-proteins tested in Codebook yielded clear promoter-located peaks in the ChIP-seq experiments. The ChIP-seq data also enriched PWMs for a variety of well-known promoter TFs but did not show enrichment of specific motifs in any other experiments. We examined existing protein–DNA structures data for THAP proteins: There is an X-ray crystallography structure for *D. melanogaster* DmTHAP (**PDB: 3KDE^184^**) and an NMR structure of human THAP1 (**PDB:2KO0^185^**). The THAPs are clearly DNA-binding proteins that bind DNA with their C2CH zinc finger domains, and at least in these two examples, form also direct contacts with DNA bases. Based on previous studies, however, both dmTHAP and THAP1 bind their specific target sites with low affinities of 160nM and >480nM, respectively. We propose that DNA binding of these proteins may be dependent on the presence of other promoter-associated chromatin components.

| **THAP10** | **ChIP** | **GHT** | **HT** | **SMS** | **PBM** | **Lysate** | **eGFP-IVT** | **IVT** |
| --- | --- | --- | --- | --- | --- | --- | --- | --- |
| **Tested?** | TRUE | TRUE | TRUE | TRUE | TRUE | TRUE | TRUE | TRUE |
| **THAP11** | **ChIP** | **GHT** | **HT** | **SMS** | **PBM** | **Lysate** | **eGFP-IVT** | **IVT** |
| **Tested?** | TRUE | TRUE | TRUE | TRUE | TRUE | TRUE | TRUE | TRUE |
| **THAP2** | **ChIP** | **GHT** | **HT** | **SMS** | **PBM** | **Lysate** | **eGFP-IVT** | **IVT** |
| **Tested?** | TRUE | TRUE | TRUE | TRUE | TRUE | TRUE | TRUE | TRUE |
| **THAP4** | **ChIP** | **GHT** | **HT** | **SMS** | **PBM** | **Lysate** | **eGFP-IVT** | **IVT** |
| **Tested?** | TRUE | TRUE | TRUE | FALSE | TRUE | TRUE | TRUE | TRUE |
| **THAP5** | **ChIP** | **GHT** | **HT** | **SMS** | **PBM** | **Lysate** | **eGFP-IVT** | **IVT** |
| **Tested?** | TRUE | TRUE | TRUE | TRUE | TRUE | TRUE | TRUE | TRUE |
| **THAP6** | **ChIP** | **GHT** | **HT** | **SMS** | **PBM** | **Lysate** | **eGFP-IVT** | **IVT** |
| **Tested?** | TRUE | TRUE | TRUE | TRUE | TRUE | TRUE | TRUE | TRUE |
| **THAP7** | **ChIP** | **GHT** | **HT** | **SMS** | **PBM** | **Lysate** | **eGFP-IVT** | **IVT** |
| **Tested?** | TRUE | TRUE | TRUE | TRUE | TRUE | TRUE | TRUE | TRUE |
| **THAP8** | **ChIP** | **GHT** | **HT** | **SMS** | **PBM** | **Lysate** | **eGFP-IVT** | **IVT** |
| **Tested?** | TRUE | TRUE | TRUE | TRUE | TRUE | FALSE | TRUE | TRUE |
| **THAP9** | **ChIP** | **GHT** | **HT** | **SMS** | **PBM** | **Lysate** | **eGFP-IVT** | **IVT** |
| **Tested?** | TRUE | TRUE | TRUE | TRUE | TRUE | FALSE | TRUE | TRUE |

### (e) Other known-DBD-containing proteins that are potential sequence specific TFs

**KDM5B (ARID/BRIGHT) is a potential TF.** The main source of evidence for the role of this protein as a TF is its ARID-BRIGHT domain, and an external experiment that is enriched with a credible motif (**Supplementary Data 1**)

**Codebook experiment summary**: No SMS experiment. All other experiment types were performed but none of them yielded specific PWMs.

| **KDM5B** | **ChIP** | **GHT** | **HT** | **SMS** | **PBM** | **Lysate** | **eGFP-IVT** | **IVT** |
| --- | --- | --- | --- | --- | --- | --- | --- | --- |
| **Tested?** | TRUE | TRUE | TRUE | FALSE | TRUE | TRUE | TRUE | TRUE |

**ZBED3 (BED ZF) a potential TF**. This protein has a ZBED DBD. There are no indications of it not being sequence specific (see **Supplementary Discussion** “Transposon-derived TFs” for more discussion on this structural class).

**Codebook experiment summary**: ChIP-seq did not pass quality control. All other experiment types were performed but without specific PWM enrichment

| **ZBED3** | **ChIP** | **GHT** | **HT** | **SMS** | **PBM** | **Lysate** | **eGFP-IVT** | **IVT** |
| --- | --- | --- | --- | --- | --- | --- | --- | --- |
| **Tested?** | TRUE | TRUE | TRUE | TRUE | TRUE | TRUE | TRUE | TRUE |

**SOHLH1 (bHLH) is a Potential TF.** This protein has a well-established bHLH DBD. Indirect evidence suggests that it is a sequence specific TF that potentially binds DNA as a heterodimer with SOHLH2^186^.

**Codebook experiment summary**: ChIP-seq did not pass quality control. All other experiment types were performed but none of them yielded specific PWMs.

| **SOHLH1** | **ChIP** | **GHT** | **HT** | **SMS** | **PBM** | **Lysate** | **eGFP-IVT** | **IVT** |
| --- | --- | --- | --- | --- | --- | --- | --- | --- |
| **Tested?** | TRUE | TRUE | TRUE | TRUE | TRUE | TRUE | TRUE | TRUE |

**DMRTB1 (DM) is a likely TF**. This protein has a DM domain. Some of the DM-domain-containing proteins are well-established TFs and an external experiment is enriched with a credible motif (**Supplementary Data 1**).

**Codebook experiment summary**: All experiment types were performed but none of them yielded specific PWMs.

| **DMRTB1** | **ChIP** | **GHT** | **HT** | **SMS** | **PBM** | **Lysate** | **eGFP-IVT** | **IVT** |
| --- | --- | --- | --- | --- | --- | --- | --- | --- |
| **Tested?** | TRUE | TRUE | TRUE | TRUE | TRUE | TRUE | TRUE | TRUE |

**GATAD2B (GATA) is a likely TF**. This protein has a GATA domain. Some of the GATA-domain proteins are well-established TFs and an external experiment is enriched with a credible motif (**Data S1**)

**Codebook experiment summary**: All experiment types were performed but none of them yielded specific PWMs.

| **GATAD2B** | **ChIP** | **GHT** | **HT** | **SMS** | **PBM** | **Lysate** | **eGFP-IVT** | **IVT** |
| --- | --- | --- | --- | --- | --- | --- | --- | --- |
| **Tested?** | TRUE | TRUE | TRUE | TRUE | TRUE | TRUE | TRUE | TRUE |

**GTF2IRD2B (GTF2I-like and potentially also a BED-zf like domain) is likely a sequence specific TF**. We tested this protein and its close paralog GTF2IRD2, the latter of which was successful and enriched a clear motif in ChIP-seq and lysate-based SELEX experiments. Both of these proteins have a GTF2I-like DBD that binds DNA specifically based on SELEX data from an individual study that characterized GTF2I^187^. Additionally, they have a C-terminal domain belonging to IPR012337 “Ribonuclease H-like superfamily” that is related to BED-zfs and could also be the DBD that binds the observed PWM (see **Supplementary Discussion** “Transposon-derived TFs”**,** for further discussion of the BED-zf domains).

Old SELEX data for GTF2I is discordant with the PWM enriched in our GTF2IRD2 experiments. GTF2I does not have the BED-zf-like domain, and the discrepancy could be due to this. Examination of the literature experiment for GTF2I shows that it has lost diversity during the selection cycles (as many of the reads are identical) and thus, the older comparison PWM may be erroneous even if the GTF2IRD2 site is enriched by GTF2I.

**Codebook experiment summary**: ChIP-seq did not pass quality control. All other experiment types were performed but none of them were enriched in any motifs.

| **GTF2IRD2B** | **ChIP** | **GHT** | **HT** | **SMS** | **PBM** | **Lysate** | **eGFP-IVT** | **IVT** |
| --- | --- | --- | --- | --- | --- | --- | --- | --- |
| **Tested?** | TRUE | TRUE | TRUE | TRUE | TRUE | TRUE | TRUE | TRUE |

**ADNP and ADNP2 (Homeodomain) are transcriptional cofactors with potential intrinsic specificity.** The main source of evidence for their role as a TFs is that they each possess a homeodomain DBD. In addition, they are predicted to have many loosely spaced noncanonical C2H2-zf domains (predicted only by SMART). Based on AF3, some of the predicted C2H2-zf domains in ADNP fold correctly but are directly adjacent to other kind of domains or even interact with them, whereas in ADNP2, one of the C2H2-zf domains is within unstructured context. Recent research has shown that these proteins are components of ChAHP and ChAHP2 chromatin remodeling complexes, respectively, which also contain CHD4 and HP1, and function to silence repeat elements. It is conceivable that their target sites are defined mostly by H3K9 trimethylation^188^, but it is also possible that they encode sequence specificity that is not detectable by our assays.

**Codebook experiment summary**: ChIP-seq experiments displayed a strong enrichment for CTCF target sites, and additionally, ADNP enriched in an unknown motif. They were tested and unsuccessful with all other approaches, however.

| **ADNP** | **ChIP** | **GHT** | **HT** | **SMS** | **PBM** | **Lysate** | **eGFP-IVT** | **IVT** |
| --- | --- | --- | --- | --- | --- | --- | --- | --- |
| **Tested?** | TRUE | TRUE | TRUE | TRUE | TRUE | TRUE | TRUE | TRUE |
| **ADNP2** | **ChIP** | **GHT** | **HT** | **SMS** | **PBM** | **Lysate** | **eGFP-IVT** | **IVT** |
| **Tested?** | TRUE | TRUE | TRUE | TRUE | TRUE | TRUE | TRUE | TRUE |

**NANOGNB (Homeodomain) is a potential TF.** This protein has a homeodomain DBD. Very little is known about the protein. AF3 predicts that the DNA recognition helix is much longer than what is required for binding DNA.

**Codebook experiment summary**: All experiment types were performed but none of them yielded specific PWMs.

| **NANOGNB** | **ChIP** | **GHT** | **HT** | **SMS** | **PBM** | **Lysate** | **eGFP-IVT** | **IVT** |
| --- | --- | --- | --- | --- | --- | --- | --- | --- |
| **Tested?** | TRUE | TRUE | TRUE | TRUE | TRUE | TRUE | TRUE | TRUE |

**ZHX2 and ZHX3 (Homeodomain) are transcriptional cofactors with potential intrinsic specificity.** ZHX1, ZHX2, and ZHX3 have all four homeodomain-like domains and potentially two C2H2-zf domains. AF3 predicts that both ZHX2 and ZHX3 have a single properly folded C2H2-zf domain, whereas the putative second C2H2-zf domain is folded into a larger domain. Furthermore, it predicts that the first C2H2-zf domain interacts with other parts of the protein, potentially inhibiting it from interacting with DNA. Relatively little is known about these proteins, but they likely form homo- and heterodimers with each other, and interact with the heterotrimeric NFY TF complex, leading to repression of transcription^189^. The only direct evidence of direct DNA binding by these proteins is that ZHX1 recognizes a low information content “ACG” site in previous PBM assays^52^. Interaction with NFY, combined with large number of homeodomains, yet showing very low information content target site in a single experiment, suggests that these proteins are likely cofactors, but they could also have inherent specificity that impacts the selection or function of loci to which they are recruited.

**Codebook experiment summary**: All experiment types were performed for both proteins but without specific PWM enrichment,

| **ZHX2** | **ChIP** | **GHT** | **HT** | **SMS** | **PBM** | **Lysate** | **eGFP-IVT** | **IVT** |
| --- | --- | --- | --- | --- | --- | --- | --- | --- |
| **Tested?** | TRUE | TRUE | TRUE | TRUE | TRUE | TRUE | TRUE | TRUE |
| **ZHX3** | **ChIP** | **GHT** | **HT** | **SMS** | **PBM** | **Lysate** | **eGFP-IVT** | **IVT** |
| **Tested?** | TRUE | TRUE | TRUE | TRUE | TRUE | TRUE | TRUE | TRUE |

**HSFX1 and HSFX2 (HSF) are potential TFs.** These proteins have an HSF domain, which is the main source of evidence for a role as a TF. Little is known about both proteins, and there is no evidence besides negative results in our assays to indicate that they are not TFs.

**Codebook experiment summary**: ChIP-seq did not pass quality control. All other experiment types were performed but none of them yielded specific PWMs.

| **HSFX1** | **ChIP** | **GHT** | **HT** | **SMS** | **PBM** | **Lysate** | **eGFP-IVT** | **IVT** |
| --- | --- | --- | --- | --- | --- | --- | --- | --- |
| **Tested?** | TRUE | TRUE | TRUE | TRUE | TRUE | TRUE | TRUE | TRUE |
| **HSFX2** | **ChIP** | **GHT** | **HT** | **SMS** | **PBM** | **Lysate** | **eGFP-IVT** | **IVT** |
| **Tested?** | TRUE | TRUE | TRUE | TRUE | TRUE | TRUE | TRUE | TRUE |

**MBD4 and MBD6 (MBD) are likely sequence specific TFs.** These proteins have an MBD domain. MBD4 binds CpG hemi-methylated DNA (one of the chains is methylated and the second one is not) in the crystal structure PDB:2MOE^190^. Some sources suggest that MBD6 does not bind methylated DNA and the MBD domain instead facilitates protein-protein interactions^191^.

**Codebook experiment summary**: No PWM enrichment in any assay; *in vitro* experiments were performed with unmethylated DNA.

| **MBD4** | **ChIP** | **GHT** | **HT** | **SMS** | **PBM** | **Lysate** | **eGFP-IVT** | **IVT** |
| --- | --- | --- | --- | --- | --- | --- | --- | --- |
| **Tested?** | TRUE | TRUE | TRUE | TRUE | TRUE | TRUE | TRUE | TRUE |
| **MBD6** | **ChIP** | **GHT** | **HT** | **SMS** | **PBM** | **Lysate** | **eGFP-IVT** | **IVT** |
| **Tested?** | TRUE | TRUE | TRUE | TRUE | TRUE | TRUE | TRUE | TRUE |

**SETDB2 (MBD) is likely a sequence specific TF.** This protein has a MBD domain, which is the main source of evidence for a role as a TF. At least some MBD domains bind CpG-methylated DNA.

**Codebook experiment summary**: ChIP-seq did not pass quality control. All experiment types were performed but none of them yielded specific PWMs. None of the *in vitro* experiments were performed with methylated DNA.

| **SETDB2** | **ChIP** | **GHT** | **HT** | **SMS** | **PBM** | **Lysate** | **eGFP-IVT** | **IVT** |
| --- | --- | --- | --- | --- | --- | --- | --- | --- |
| **Tested?** | TRUE | TRUE | TRUE | TRUE | TRUE | TRUE | TRUE | TRUE |

**MTERF2, MTERF3, and MTERF4 (mTERF) are Potential TFs**. The main source of evidence for a role as a TF is that they have an mTERF DBD. DNA recognition of the most well characterized paralog MTERF1 has been characterized with a crystal structure **PDB:3MVA^192^**. MTERF1 terminates mitochondrial transcription through binding DNA with a mechanism that leads to a large strain on the DNA shape and flips out a base pair from the double-helical context. The binding site is very long.

**Codebook experiment summary**: No SMS experiment. ChIP-seq did not pass quality control. All other experiment types were performed but none of them yielded specific PWMs.

| **MTERF2** | **ChIP** | **GHT** | **HT** | **SMS** | **PBM** | **Lysate** | **eGFP-IVT** | **IVT** |
| --- | --- | --- | --- | --- | --- | --- | --- | --- |
| **Tested?** | TRUE | TRUE | TRUE | FALSE | TRUE | TRUE | TRUE | TRUE |
| **MTERF3** | **ChIP** | **GHT** | **HT** | **SMS** | **PBM** | **Lysate** | **eGFP-IVT** | **IVT** |
| **Tested?** | FALSE | TRUE | TRUE | FALSE | TRUE | FALSE | TRUE | TRUE |
| **MTERF4** | **ChIP** | **GHT** | **HT** | **SMS** | **PBM** | **Lysate** | **eGFP-IVT** | **IVT** |
| **Tested?** | TRUE | TRUE | TRUE | FALSE | TRUE | TRUE | TRUE | TRUE |

**TERB1 (Myb/SANT) is a potential TF.** This protein has a Myb/SANT domain, which is the main source of evidence for a role as a TF. It contains only a single Myb domain (with slightly less similarity to a SANT domain model). This domain has been shown to be involved in protein-protein interactions^193^, but this may not be mutually exclusive with DNA-binding.

**Codebook experiment summary**: All experiment types were performed but none of them yielded specific PWMs.

| **TERB1** | **ChIP** | **GHT** | **HT** | **SMS** | **PBM** | **Lysate** | **eGFP-IVT** | **IVT** |
| --- | --- | --- | --- | --- | --- | --- | --- | --- |
| **Tested?** | TRUE | TRUE | TRUE | TRUE | TRUE | TRUE | TRUE | TRUE |

**NFX1 and NFXL1 (NFX) are potential TFs.** NFX1 has been indicated to be a TF, based on EMSA experiments performed with recombinant *E. coli*-expressed protein^194^ and, for NFXL1, there are recent PWMs derived from ENCODE ChIP-seq (available in Factorbook database). One of the Factorbook motifs is selected in this study as a representative PWM for NFXL1 (**Supplementary Table 13** and **Supplementary Data 1**). The two datasets are discrepant, however. Based on the old EMSA study, NFX1 binds to DNA fragment with a sequence: 5’ CCCTTCCCCTAGCAAGAGATG 3’, (underscored part is the genomic site predicted by the authors), whereas some of the ChIP-seq-derived PWMs for NFXL1 are somewhat similar to PAX sites and others are low complexity G- and A-rich sequences. We selected one of the PAX-like PWMs as the representative PWM (Consensus: “CYRARGTCACASAGC”).

Additional evidence supporting NFX domains as DBDs is an HT-SELEX experiment in *E. coli* that derived PWM for a fruit fly ortholog, *stc* (shuttlecraft), with very different consensus sequence “TATCAYAWKRTGATA"^195^. This discrepancy is expected, however, as both the number of NFX domains (11 in NFXL1 and 7 in *stc*) and their sequences have diverged substantially.

On the other hand, there is evidence that links these proteins to RNA-associated functions^196^, and both NFX1 and NFXL1 are associated very clearly with ribosomes based on protein-protein interactions listed in STRING database, casting some doubt on whether these proteins are TFs with inherent specificity.

**Codebook experiment summary**: All experiment types were performed for NFX1 and all but ChIP-seq for NFXL1. None of them yielded specific PWMs.

| **NFX1** | **ChIP** | **GHT** | **HT** | **SMS** | **PBM** | **Lysate** | **eGFP-IVT** | **IVT** |
| --- | --- | --- | --- | --- | --- | --- | --- | --- |
| **Tested?** | TRUE | TRUE | TRUE | TRUE | TRUE | TRUE | TRUE | TRUE |
| **NFXL1** | **ChIP** | **GHT** | **HT** | **SMS** | **PBM** | **Lysate** | **eGFP-IVT** | **IVT** |
| **Tested?** | FALSE | TRUE | TRUE | TRUE | TRUE | FALSE | TRUE | TRUE |

**SP110 (SAND) is a potential TF**. This protein has a SAND domain. Many SAND domain TFs are supported by modern systematic datasets. Mouse Sp110 bound DNA in PBM experiments, but the human and mouse proteins are diverged.

**Codebook experiment summary**: ChIP-seq did not pass quality control, and none of the other experiments enriched in motifs.

| **SP110** | **ChIP** | **GHT** | **HT** | **SMS** | **PBM** | **Lysate** | **eGFP-IVT** | **IVT** |
| --- | --- | --- | --- | --- | --- | --- | --- | --- |
| **Tested?** | TRUE | TRUE | TRUE | TRUE | TRUE | TRUE | TRUE | TRUE |

**TBPL1 (TBP) is a potential TF.** This protein has a TBP domain. The Codebook ChIP-seq was enriched in typical promoter motifs, suggesting that this protein functions in a similar fashion as its paralog TBP. It is a relatively distant paralog of TBP, and the only member of the TBP family that lacks the ability to bind the TATA box^197^. *In vitro*, purified TLF-TFIIA binds directly to the NF1 promoter^198^, so it may be an obligate heteromer.

**Codebook experiment summary**: No SMS experiment. ChIP-seq is enriched in promoter-specific signals including YY1 and NRF1. None of the other assays enriched any motifs.

| **TBPL1** | **ChIP** | **GHT** | **HT** | **SMS** | **PBM** | **Lysate** | **eGFP-IVT** | **IVT** |
| --- | --- | --- | --- | --- | --- | --- | --- | --- |
| **Tested?** | TRUE | TRUE | TRUE | FALSE | TRUE | TRUE | TRUE | TRUE |

### (f) Proteins with unknown DBDs that are unlikely to be sequence specific TFs

**CENPA (Unknown) is not likely to be a TF.** The main source of evidence for a role as a TF was PDB:3AN2. This protein is a histone H3 variant protein that binds same way as H3 in a regular histone, based on crystal structure PDB:3AN2.

**Codebook experiment summary:** No SMS experiment. All other experiment types were performed but without specific PWM enrichment.

| **CENPA** | **ChIP** | **GHT** | **HT** | **SMS** | **PBM** | **Lysate** | **eGFP-IVT** | **IVT** |
| --- | --- | --- | --- | --- | --- | --- | --- | --- |
| **Tested?** | TRUE | TRUE | TRUE | FALSE | TRUE | TRUE | TRUE | TRUE |

**AEBP1 (Unknown) is not likely to be a TF.** This protein was not annotated to have any likely DBDs. It has been suggested to moonlight as a TF, and to bind DNA based on EMSA^199^ and ChIP^200^. It has a well characterized function as a protease (carboxypeptidase), however.

**Codebook experiment summary**: No SMS experiment. ChIP-seq did not pass quality control. All other experiment types were performed, but none of them yielded specific PWMs.

| **AEBP1** | **ChIP** | **GHT** | **HT** | **SMS** | **PBM** | **Lysate** | **eGFP-IVT** | **IVT** |
| --- | --- | --- | --- | --- | --- | --- | --- | --- |
| **Tested?** | TRUE | TRUE | TRUE | FALSE | TRUE | TRUE | TRUE | TRUE |

**ARHGAP35 (Unknown) is not likely to be a TF**. This protein was not annotated to have any likely DBDs. It has been indicated to bind DNA specifically based on EMSA and nuclease protection assay data^201^. It has a well characterized function as A GTPase-activating protein (GAP) functioning in RhoGAP signaling, however^202^. Its interactions with Rho and Rac GTPases and other cytoskeleton components are also well supported by systematic analyses on protein-protein interaction data in STRING database.

**Codebook experiment summary**: No SMS experiment. ChIP-seq did not pass quality control. All other experiment types were performed, but none of them yielded specific PWMs.

| **ARHGAP35** | **ChIP** | **GHT** | **HT** | **SMS** | **PBM** | **Lysate** | **eGFP-IVT** | **IVT** |
| --- | --- | --- | --- | --- | --- | --- | --- | --- |
| **Tested?** | TRUE | TRUE | TRUE | FALSE | TRUE | TRUE | TRUE | TRUE |

**CC2D1A (Unknown) is not likely to be a TF.** This protein was not annotated to have any likely DBDs. It has been shown to bind DNA specifically on recombinant protein based EMSA and nuclease protection assay data^203^. However, it makes strong interactions with endosomal transport associated proteins

**Codebook experiment summary**: No IVT SELEX, SMS or PBM experiments. ChIP-seq did not pass quality control. All other experiment types were performed but none of them yielded specific PWMs.

| **CC2D1A** | **ChIP** | **GHT** | **HT** | **SMS** | **PBM** | **Lysate** | **eGFP-IVT** | **IVT** |
| --- | --- | --- | --- | --- | --- | --- | --- | --- |
| **Tested?** | TRUE | TRUE | TRUE | FALSE | FALSE | TRUE | TRUE | FALSE |

**CENPT (Unknown) is a centromere associated protein complex component and not likely to be a TF.** This protein was not annotated to have any likely sequence specific DNA-binding domains. It contains an archaeal histone-like fold (CBF_NFY), however, and has a known role in centromere assembly. It makes DNA backbone contacts and potentially has a minor groove inserting arginine in cryo-EM structure 7R5S^204^. In the structure it is intertwined together with CENPW and can likely bind DNA with it as a heterodimer, based on DNA contacts the binding is likely to have low specificity.

**Codebook experiment summary**: No SMS experiments. ChIP-seq did not pass quality control. All other experiment types were performed but none of them yielded specific PWMs.

| **CENPT** | **ChIP** | **GHT** | **HT** | **SMS** | **PBM** | **Lysate** | **eGFP-IVT** | **IVT** |
| --- | --- | --- | --- | --- | --- | --- | --- | --- |
| **Tested?** | TRUE | TRUE | TRUE | FALSE | TRUE | TRUE | TRUE | TRUE |

**CHCHD3 (Unknown) is not likely to be a TF.** This protein was not annotated to have any likely DBDs. Sequence specific binding activity has been indicated by EMSA utilizing recombinant protein produced in *E. coli*, showing that it can specially bind the hBAG-1 promoter^205^. However, the protein is a known mitochondrial membrane scaffold protein.

**Codebook experiment summary**: No SMS experiment. ChIP-seq did not pass quality control. All other experiment types were performed but none of them yielded specific PWMs.

| **CHCHD3** | **ChIP** | **GHT** | **HT** | **SMS** | **PBM** | **Lysate** | **eGFP-IVT** | **IVT** |
| --- | --- | --- | --- | --- | --- | --- | --- | --- |
| **Tested?** | TRUE | TRUE | TRUE | FALSE | TRUE | TRUE | TRUE | TRUE |

**CSRNP1, CSRNP2, and CSRNP3 (Unknown) may be transcriptional cofactors.** These proteins were not annotated to have any likely DBDs. All three paralogs (CSRNP1, CSRNP2, and CSRNP3) were reported to bind an “AGAGTG” consensus in SELEX experiments performed with recombinant HEK293 expressed proteins^206^. However, the study has insufficient details about the SELEX experiments for evaluation of its results, including entire sequences of the 30-bp DNA fragments. Based on protein-protein interactions and mostly nuclear localization, these proteins could be cofactors.

**Codebook experiment summary**: All experiment types were performed but only SMiLE-seq^13^ showed enrichment of specific PWMs.

| **CSRNP1** | **ChIP** | **GHT** | **HT** | **SMS** | **PBM** | **Lysate** | **eGFP-IVT** | **IVT** |
| --- | --- | --- | --- | --- | --- | --- | --- | --- |
| **Tested?** | TRUE | TRUE | TRUE | TRUE | TRUE | TRUE | TRUE | TRUE |
| **CSRNP2** | **ChIP** | **GHT** | **HT** | **SMS** | **PBM** | **Lysate** | **eGFP-IVT** | **IVT** |
| **Tested?** | TRUE | TRUE | TRUE | TRUE | TRUE | TRUE | TRUE | TRUE |
| **CSRNP3** | **ChIP** | **GHT** | **HT** | **SMS** | **PBM** | **Lysate** | **eGFP-IVT** | **IVT** |
| **Tested?** | TRUE | TRUE | TRUE | TRUE | TRUE | TRUE | TRUE | TRUE |

**GLMP (Unknown) is not likely to be a TF or a cofactor.** This protein was not annotated to have any likely DBDs. Sequence specificity is indicated by an EMSA experiment with a recombinant protein, showing that it binds GATCCGCCCGCTTGTGGCCAACTGGCTCCAGTCAC^207^. It is a known lysosomal membrane protein, however.

**Codebook experiment summary**: No SMS experiment. ChIP-seq did not pass quality control. All other experiment types were performed but none of them yielded specific PWMs.

| **GLMP** | **ChIP** | **GHT** | **HT** | **SMS** | **PBM** | **Lysate** | **eGFP-IVT** | **IVT** |
| --- | --- | --- | --- | --- | --- | --- | --- | --- |
| **Tested?** | TRUE | TRUE | TRUE | FALSE | TRUE | TRUE | TRUE | TRUE |

**GPBP1 and GPBP1L1 (Unknown) are unlikely to be TFs.** These proteins were not annotated to have any likely DBDs. The main source of evidence for a role of GPBP1 (also known as Vasculin) as a TF is a single study with a recombinant protein based EMSA showing that it binds DNA^208^, however another study shows that it has an “extended AT-hook” DNA-binding peptide motif that has stronger affinity towards RNA than DNA^209^. GPBP1L1 is a close paralog of GPBP1, but very little is known of it. GPBP1 and GPBP1L1 form protein–protein interactions mostly with RNA processing connected proteins.

**Codebook experiment summary**: No SMS experiment. ChIP-seq did not pass quality control. All other experiment types were performed but none of them yielded specific PWMs.

| **GPBP1** | **ChIP** | **GHT** | **HT** | **SMS** | **PBM** | **Lysate** | **eGFP-IVT** | **IVT** |
| --- | --- | --- | --- | --- | --- | --- | --- | --- |
| **Tested?** | TRUE | TRUE | TRUE | FALSE | TRUE | TRUE | TRUE | TRUE |
| **GPBP1L1** | **ChIP** | **GHT** | **HT** | **SMS** | **PBM** | **Lysate** | **eGFP-IVT** | **IVT** |
| **Tested?** | TRUE | TRUE | TRUE | FALSE | TRUE | TRUE | TRUE | TRUE |

**KCNIP3 (Unknown) is not likely to be a TF**. This protein was not annotated to have any likely DBDs. Sequence specific DNA binding activity was shown by recombinant protein expression based EMSA experiment^140^. However, the protein has a well-established role in binding to and controlling specific types of potassium channels. This protein has been researched extensively but there is very little further evidence of sequence specific DNA binding activity.

**Codebook experiment summary**: No SMS experiments. All other experiment types were performed but none of them yielded specific PWMs.

| **KCNIP3** | **ChIP** | **GHT** | **HT** | **SMS** | **PBM** | **Lysate** | **eGFP-IVT** | **IVT** |
| --- | --- | --- | --- | --- | --- | --- | --- | --- |
| **Tested?** | TRUE | TRUE | TRUE | FALSE | TRUE | TRUE | TRUE | TRUE |

**NKRF (Unknown) is not likely to be a TF**. This protein was not annotated to have any likely DBDs. It is known to regulate NFKB activity, indicating a role in transcriptional regulation. EMSA experiments performed with induced nuclear extracts have indicated binding to AATTTCCTCTGA^210^ and to AATTCCCTGA^211^. A recent study, however, indicates that NKRF binds NFKB directly to inhibit its activity^212^. Protein interaction data in STRINGdb suggest a role in RNA editing.

**Codebook experiment summary**: ChIP-seq did not pass quality control. All other experiment types were performed but none of them yielded specific PWMs.

| **NKRF** | **ChIP** | **GHT** | **HT** | **SMS** | **PBM** | **Lysate** | **eGFP-IVT** | **IVT** |
| --- | --- | --- | --- | --- | --- | --- | --- | --- |
| **Tested?** | TRUE | TRUE | TRUE | TRUE | TRUE | TRUE | TRUE | TRUE |

**NME2 (Unknown) is not likely to be a TF**. This protein was not annotated to have any likely DBDs. One study indicated that it binds to specific sequences in nuclear extract based EMSA^213^, and another that it associated with telomeres based on ChIP-seq^214^. The protein is an enzyme, however (nucleoside diphosphate kinase) and the DNA in the structure PDB:3BBB^215^ is a single-stranded dinucleotide. Taken together, and with the negative Codebook results, the evidence suggests that it is not a sequence specific TF.

**Codebook experiment summary**: No ChIP-seq experiment. All other experiments performed but without specific PWM enrichment.

| **NME2** | **ChIP** | **GHT** | **HT** | **SMS** | **PBM** | **Lysate** | **eGFP-IVT** | **IVT** |
| --- | --- | --- | --- | --- | --- | --- | --- | --- |
| **Tested?** | FALSE | TRUE | TRUE | TRUE | TRUE | TRUE | TRUE | TRUE |

**PA2G4 (Unknown) is likely a low specificity DNA-binding cofactor**. This protein was not annotated to have any likely DBDs. It binds CGGCAAAAAGG repeats, based on EMSA performed with recombinant protein^216^. Many other lines of evidence including cryo-EM structure **PDB: 6SXO** show that it is a double-stranded ribosomal RNA binding protein and a co-regulator^217,218^.

**Codebook experiment summary**: No SMS experiment. ChIP-seq did not pass quality control. All other experiment types were performed but none of them yielded specific PWMs.

| **^219^G4** | **ChIP** | **GHT** | **HT** | **SMS** | **PBM** | **Lysate** | **eGFP-IVT** | **IVT** |
| --- | --- | --- | --- | --- | --- | --- | --- | --- |
| **Tested?** | TRUE | TRUE | TRUE | FALSE | TRUE | TRUE | TRUE | TRUE |

**PCGF2 and PCGF6 (Unknown) are likely to be transcriptional cofactors.** These paralogs were not annotated to have any likely DBDs, as RING type zinc finger domain in them is a protein-protein interaction interphase used to contact other RING domains. A study from 1995 study showed that PCGF2 binds “GACTNGACT“ based on SELEX and EMSA performed with cell expressed recombinant proteins, and that binding can be achieved also in EMSA performed with *E. coli* expressed protein^219^. Substantial amount of research has since shown alternative recruitment strategies for these protein complexes^220^. The PCGF2 and PCGF6 proteins function in a PRC1.2 and PRC1.6 polycomb complexes, respectively. PRC1.2 has been shown to be recruited mainly through CBX protein mediated interactions where the chromodomain of the CBX contacts H3K27me3 to silence chromatin. PRC1.6, on the other hand, has been shown to be recruited by heterodimeric combinations of MAX+MNT (bHLH) and E2F6+DP1 (E2F), and concordantly, our ChIP-seq experiments show enrichement of “CACGTG”- and “GGCGGGAAA”-containing PWMs^220^.

**Codebook experiment summary**: No SMS experiment for either. ChIP-seq experiments for PCGF2 did not pass quality control, whereas PCGF6 enriched in “CACGTG”-sequences bound by many bHLH TFs and CG-rich sequences. None of the other experiment types yielded specific PWMs.

| **PCGF2** | **ChIP** | **GHT** | **HT** | **SMS** | **PBM** | **Lysate** | **eGFP-IVT** | **IVT** |
| --- | --- | --- | --- | --- | --- | --- | --- | --- |
| **Tested?** | TRUE | TRUE | TRUE | FALSE | TRUE | TRUE | TRUE | TRUE |
| **PCGF6** | **ChIP** | **GHT** | **HT** | **SMS** | **PBM** | **Lysate** | **eGFP-IVT** | **IVT** |
| **Tested?** | TRUE | TRUE | TRUE | FALSE | TRUE | TRUE | TRUE | TRUE |

**PLSCR1 (Unknown) is not likely to be a TF.** This protein was not annotated to have any likely DBDs. Recombinant protein based EMSA evidence indicates that this enzyme can bind to a “GTAACCATGTGGA” sequence that is present in the IP3R1 promoter^221^. Protein has been researched very extensively^222^, it is best characterized as membrane bound enzyme (phospholipid scramblase), that localizes mostly to plasma membrane but is likely actively transported to intracellular compartments as response to various stimuli. In nucleus it has been shown to associate with several transcription factors and through them to different promoters^222^ Based on these lines of evidence, and that it did not enrich specific PWMs in any of our analyses it functions most likely as a cofactor.

**Codebook experiment summary**: No SMS experiment and ChIP-seq did not pass quality control. No other experiment types enriched specific PWMs.

| **PLSCR1** | **ChIP** | **GHT** | **HT** | **SMS** | **PBM** | **Lysate** | **eGFP-IVT** | **IVT** |
| --- | --- | --- | --- | --- | --- | --- | --- | --- |
| **Tested?** | TRUE | TRUE | TRUE | FALSE | TRUE | TRUE | TRUE | TRUE |

**PREB (Unknown) is not likely to be a TF.** This protein was not annotated to have any likely DBDs. EMSA experiments indicated that it binds DNA^223^; however, many other lines of evidence link it to functioning in ER-Golgi transport as a “guanine nucleotide exchange factor” (GEF) that regulates the assembly of the coat protein complex II/COPII in endoplasmic reticulum (ER) to Golgi vesicle-mediated transport.

**Codebook experiment summary**: No SMS experiment. ChIP-seq did not pass quality control. All other experiment types were performed but none of them yielded specific PWMs.

| **PREB** | **ChIP** | **GHT** | **HT** | **SMS** | **PBM** | **Lysate** | **eGFP-IVT** | **IVT** |
| --- | --- | --- | --- | --- | --- | --- | --- | --- |
| **Tested?** | TRUE | TRUE | TRUE | FALSE | TRUE | TRUE | TRUE | TRUE |

**RBCK1 (Unknown) is not likely to be a TF.** This protein was not annotated to have any likely DBDs, and it has a well-established ubiquitin ligase. EMSA and SELEX indicated that it bound TGG trinucleotide repeats *in vitro*^224^. To our knowledge, there have been no other reports regarding its sequence specificity or function as a TF since 1998. Thus, altogether, it has poorly defined DNA sequence/structure specificity and limited support as a TF.

**Codebook experiment summary**: No SMS or PBM experiments. ChIP-seq did not pass quality control, and other experiment types did not enrich specific PWMs.

| **RBCK1** | **ChIP** | **GHT** | **HT** | **SMS** | **PBM** | **Lysate** | **eGFP-IVT** | **IVT** |
| --- | --- | --- | --- | --- | --- | --- | --- | --- |
| **Tested?** | TRUE | TRUE | TRUE | FALSE | FALSE | TRUE | TRUE | TRUE |

**REXO4 (Unknown) is not likely to be a TF.** This protein does not have any apparent DBDs, and it is a DNA/RNA exonuclease. An EMSA-based study indicated that it can enhance the ability of ESR2 to bind its targets^225^.

**Codebook experiment summary**: Only IVT based SELEX experiment. No specific PWM enrichment.

| **REXO4** | **ChIP** | **GHT** | **HT** | **SMS** | **PBM** | **Lysate** | **eGFP-IVT** | **IVT** |
| --- | --- | --- | --- | --- | --- | --- | --- | --- |
| **Tested?** | FALSE | TRUE | TRUE | FALSE | FALSE | FALSE | FALSE | TRUE |

**SAFB and SAFB2 (Unknown) are not likely to be TFs.** These proteins do not have likely DBDs, and many studies have linked them to binding RNA. Existing evidence for them being TFs are filter binding assays^226^.

**Codebook experiment summary**: No ChIP-seq, Lysate SELEX or SMS experiments. Other experiments were performed but without specific PWM enrichment.

| **SAFB** | **ChIP** | **GHT** | **HT** | **SMS** | **PBM** | **Lysate** | **eGFP-IVT** | **IVT** |
| --- | --- | --- | --- | --- | --- | --- | --- | --- |
| **Tested?** | FALSE | TRUE | TRUE | FALSE | TRUE | FALSE | TRUE | TRUE |
| **SAFB2** | **ChIP** | **GHT** | **HT** | **SMS** | **PBM** | **Lysate** | **eGFP-IVT** | **IVT** |
| **Tested?** | TRUE | TRUE | TRUE | TRUE | TRUE | TRUE | TRUE | TRUE |

**SCMH1 (Unknown) is likely a transcriptional cofactor and not a TF.** This protein does not have likely DBDs. It was included due to homology with SCML2, a polycomb repressive complex protein. It associates with polycomb complexes and is likely a cofactor.

**Codebook experiment summary**: No SMS experiment. ChIP-seq did not pass quality control. All other experiment types were performed but none of them yielded specific PWMs.

| **SCMH1** | **ChIP** | **GHT** | **HT** | **SMS** | **PBM** | **Lysate** | **eGFP-IVT** | **IVT** |
| --- | --- | --- | --- | --- | --- | --- | --- | --- |
| **Tested?** | TRUE | TRUE | TRUE | FALSE | TRUE | TRUE | TRUE | TRUE |

**SMYD3 (Unknown) is likely a transcriptional cofactor, and not a TF.** This protein was not annotated to have any likely DBDs. It is a histone methyltransferase. One report showed that it binds GGAGGG elements, based on SELEX and EMSA performed using GST fusion protein^227^.

**Codebook experiment summary**: ChIP-seq did not pass quality control. All other experiment types were performed but none of them yielded specific PWMs.

| **SMYD3** | **ChIP** | **GHT** | **HT** | **SMS** | **PBM** | **Lysate** | **eGFP-IVT** | **IVT** |
| --- | --- | --- | --- | --- | --- | --- | --- | --- |
| **Tested?** | TRUE | TRUE | TRUE | TRUE | TRUE | TRUE | TRUE | TRUE |

**SNAPC2 and SNAPC5 (Unknown) are unlikely to be TFs.** These proteins have been not annotated to have any likely DBDs and. SNAPC2 was included due to cellular extract based EMSA and antibody supershift evidence, which show that it is a part of initiation complex, but there is no evidence suggesting that it binds DNA on its own^228^. For SNAPC5, an EMSA experiment was performed with cell extract^229^. The protein is present in several initiation complex structures^230^ (PDB: 7ZWC, 7ZWD or 7ZX7) but does not contact DNA directly in any of them.

**Codebook experiment summary**: All experiment types were performed but none of them yielded specific PWMs.

| **SNAPC2** | **ChIP** | **GHT** | **HT** | **SMS** | **PBM** | **Lysate** | **eGFP-IVT** | **IVT** |
| --- | --- | --- | --- | --- | --- | --- | --- | --- |
| **Tested?** | TRUE | TRUE | TRUE | TRUE | TRUE | TRUE | TRUE | TRUE |
| **SNAPC5** | **ChIP** | **GHT** | **HT** | **SMS** | **PBM** | **Lysate** | **eGFP-IVT** | **IVT** |
| **Tested?** | FALSE | TRUE | TRUE | TRUE | TRUE | TRUE | TRUE | TRUE |

**SON (Unknown) is not likely to be a TF**. This protein was not annotated to have any likely DBDs. The main source of evidence for a role as a TF was study in which SELEX and EMSA were carried out with transfected cell extracts^231^, which indicates that it (or something it associates with) binds “GAKANSRCC”. SON is thought to be mainly an RNA-binding protein that acts as a mRNA splicing cofactor, however.

**Codebook experiment summary**: Only ChIP-seq and Lysate based SELEX were performed. ChIP-seq did not pass quality control. No specific PWM enrichment.

| **SON** | **ChIP** | **GHT** | **HT** | **SMS** | **PBM** | **Lysate** | **eGFP-IVT** | **IVT** |
| --- | --- | --- | --- | --- | --- | --- | --- | --- |
| **Tested?** | TRUE | TRUE | TRUE | FALSE | FALSE | TRUE | FALSE | FALSE |

**TET2 (Unknown) is likely a low specificity DNA-binding cofactor**. This protein was not annotated to have any likely DBDs, however, it is an enzyme that oxidises methylated CpG sequences to hydroxymethyls. It was included based on crystal structure PDB:4NM6^232^ that shows the enzymatic domain of the protein complexed with a CpG methylated DNA. Protein binds DNA on the minor groove side, making backbone contacts and showing that the methylated base is flipped out of double-stranded context to face toward the catalytic core of the enzyme. Based on many lines of evidence, the removal of CpG methylation is guided with multiple mechanisms, including both sequence specific TFs and chromatin modifications, making it unlikely that there is significant contribution of intrinsic specificity^233^.

**Codebook experiment summary**: ChIP-seq enriched specific PWMs, most prominent of which were for posterior homeodomains and SMiLE-seq showed enrichment of CG-containing DNA-when the assay was performed with CpG methylated DNA^13^, whereas all other assays were unsuccessful.

| **TET2** | **ChIP** | **GHT** | **HT** | **SMS** | **PBM** | **Lysate** | **eGFP-IVT** | **IVT** |
| --- | --- | --- | --- | --- | --- | --- | --- | --- |
| **Tested?** | TRUE | TRUE | TRUE | TRUE | TRUE | TRUE | TRUE | TRUE |

**THYN1 (Unknown) is a low specificity DNA-binding protein.** This protein was included in the study based on X-ray crystallography structure (**PDB:5J3E,** structure is not connected to a publication) with CpG methylated palindromic DNA sequence “GCCAAmCGTTGGC”. In this structure, the protein makes extensive contacts with the DNA backbone, but contacts bases only through a single arginine inserted into the minor groove. In the structure, the protein does not contact the methylated cytosine. These features of the structure and the fact that protein did not enrich any motifs in experiments suggest that its DNA binding is nonspecific.

**Codebook experiment summary**: All experiment types were performed but none of them yielded specific PWMs.

| **THYN1** | **ChIP** | **GHT** | **HT** | **SMS** | **PBM** | **Lysate** | **eGFP-IVT** | **IVT** |
| --- | --- | --- | --- | --- | --- | --- | --- | --- |
| **Tested?** | TRUE | TRUE | TRUE | TRUE | TRUE | TRUE | TRUE | TRUE |

**TMF1 (Unknown) is likely not a TF.** This protein has not been annotated to have any likely DBDs and based on AF3 prediction, it is composed entirely of long alpha helices and unstructured regions. The protein was reported to bind DNA specifically based on EMSA experiments, where bacterially expressed recombinant protein bound specifically to the TATA element^234^. A second transcriptional regulation connecting study from 1999 described it as a co-activator of androgen receptor (AR), based on it interacting well with AR and up-regulating the expression of AR targets^235^. Later on, in a study from 2007 the protein was indicated to function in Rab6 mediated retrograde transport on the levels of both, endosomes to Golgi and Golgi to ER^236^. Interactions between TMF1, AR and Golgi transport proteins are validated by systematic analyzes of protein-protein interactions, and thus the protein’s role in retrograde transport activity could potentially explain AR target upregulation seen in 1999 study. To our knowledge, however, no studies since the original EMSA-experiments have shown direct DNA-binding activity to this protein, and together with negative results in all our assays and a predicted structure that appears incompatible with DNA binding activity, it seems unlikely that the protein is a TF.

**Codebook experiment summary**: All experiment types were performed but none of them yielded specific PWMs.

| **TMF1** | **ChIP** | **GHT** | **HT** | **SMS** | **PBM** | **Lysate** | **eGFP-IVT** | **IVT** |
| --- | --- | --- | --- | --- | --- | --- | --- | --- |
| **Tested?** | TRUE | TRUE | TRUE | TRUE | TRUE | TRUE | TRUE | TRUE |

**TSC22D1 (Unknown) is likely a transcriptional cofactor.** This protein does not have any likely DBDs as it contains a potential leucine zipper, but without the basic region used to bind DNA in bZIP proteins. It was included based on an EMSA experiments by Ohta et al. 1996, performed with *E. coli* expressed recombinant protein that indicated it to bind CG-rich sequences^237^. TSC22D1 interacts with known TFs, including SMAD family members TFs to affect transcription, however^238,239^, and to our knowledge there has been no evidence of direct DNA binding activity.

**Codebook experiment summary**: Only ChIP-seq and Lysate based SELEX experiments. ChIP-seq did not pass quality control. No enrichment for specific PWMs.

| **TSC22D1** | **ChIP** | **GHT** | **HT** | **SMS** | **PBM** | **Lysate** | **eGFP-IVT** | **IVT** |
| --- | --- | --- | --- | --- | --- | --- | --- | --- |
| **Tested?** | TRUE | TRUE | TRUE | FALSE | FALSE | TRUE | FALSE | FALSE |

### (g) Potential TFs with unknown DNA-binding domain

**RAG1 (Unknown) is likely a sequence specific TF.** The main evidence for sequence specific DNA binding activity of RAG1 is X-ray structures showing the protein binding to DNA by itself (PDB: 3GNA) and also as a heteromultimer with RAG2 3(JBW)^240^. It binds DNA specifically as a part of sequence specific nuclease complex that works in V(D)J recombination, but as a sequence specific DNA binder it could also have regulatory activity. **Codebook experiment summary**: No SMS experiment. Codebook ChIP-seq data are enriched in a “ACAAAAACC” motif that is concordant with DNA sequence in crystal structure PDB: 3GNA. Other experiments were performed but without specific PWM enrichment.

| **RAG1** | **ChIP** | **GHT** | **HT** | **SMS** | **PBM** | **Lysate** | **eGFP-IVT** | **IVT** |
| --- | --- | --- | --- | --- | --- | --- | --- | --- |
| **Tested?** | TRUE | TRUE | TRUE | FALSE | TRUE | TRUE | TRUE | TRUE |

**CENPS and CENPX (Unknown) are likely sequence-specific DNA-binding proteins that bind together as a cooperative complex.** Their potential for sequence specific DNA binding activity was based on crystal structure **PDB:4NDY^241^**, which shows CENPS and CENPX binding DNA as a complex that is consistent of them being obligate heterodimerization partners of each other, as the protein chains are intertwined. DNA in the structure is an AAAAAAA-stretch.

**Codebook experiment summary**: No SMS experiment. ChIP-seq experiments did not pass quality control and *in vitro* experiments were tested only in individual contexts. None of the experiments enriched specific PWMs.

| **CENPS** | **ChIP** | **GHT** | **HT** | **SMS** | **PBM** | **Lysate** | **eGFP-IVT** | **IVT** |
| --- | --- | --- | --- | --- | --- | --- | --- | --- |
| **Tested?** | TRUE | TRUE | TRUE | FALSE | TRUE | TRUE | TRUE | TRUE |
| **CENPX** | **ChIP** | **GHT** | **HT** | **SMS** | **PBM** | **Lysate** | **eGFP-IVT** | **IVT** |
| **Tested?** | TRUE | TRUE | TRUE | FALSE | TRUE | TRUE | TRUE | TRUE |

**SKI and SKIL (Unknown) are likely sequence specific TFs**. These proteins have a SKI/SNO/DACH DNA binding domain (see **Supplementary Discussion** “Under-characterized DBDs, including the SKI/SNO/DAC and C-Clamp domains” for more details). The main evidence of sequence specific DNA binding activity is a study (Nicol et al. 1998) reporting that, by SELEX and EMSA, SKI (produced from cell extract) binds a “GTCTAGAC” consensus^95^.

**Codebook experiment summary**: ChIP-seq experiments did not pass quality control. The same consensus sequence described by Nicol et al. clearly enriches in our lysate-based HT-SELEX for SKIL, however. This site enriched only in one weak experiment, and therefore the PWM did not pass our quality control. Nonetheless, it clearly validates the sequence specific DNA-binding activity of the protein. Both our SKIL experiment and the Nicol et, al. data are based on cellular extracts, and as these proteins were unsuccessful with other protein production methods, it is possible that they require a post-transcriptional modification, bind as heterodimers, or potentially contact DNA indirectly.

| **SKI** | **ChIP** | **GHT** | **HT** | **SMS** | **PBM** | **Lysate** | **eGFP-IVT** | **IVT** |
| --- | --- | --- | --- | --- | --- | --- | --- | --- |
| **Tested?** | TRUE | TRUE | TRUE | FALSE | FALSE | FALSE | TRUE | FALSE |
| **SKIL** | **ChIP** | **GHT** | **HT** | **SMS** | **PBM** | **Lysate** | **eGFP-IVT** | **IVT** |
| **Tested?** | TRUE | TRUE | TRUE | FALSE | FALSE | TRUE | TRUE | FALSE |

**DRAP1 (Unknown) is a potential TF.** This protein was not annotated to have any likely DBDs. DRAP1 produced AT-rich motif in external ChIP-seq that is consistent with its known function of binding DNA as cooperative complex of DR1, DRAP1 and TBP in structure PDB: 1JFI^242^

**Codebook experiment summary**: No SMS experiment. ChIP-seq is enriched in NFY-like PWMs. Other experiment types did not yield clear enrichment.

| **DRAP1** | **ChIP** | **GHT** | **HT** | **SMS** | **PBM** | **Lysate** | **eGFP-IVT** | **IVT** |
| --- | --- | --- | --- | --- | --- | --- | --- | --- |
| **Tested?** | TRUE | TRUE | TRUE | FALSE | TRUE | TRUE | TRUE | TRUE |

**BRF2 (Unknown) is likely a sequence specific TF.** This protein was not annotated to have any likely DBDs. Based on PDB:4ROC it binds cooperatively with TBP and defines flanking specificity^243^; proper interaction needs the DNA bending caused by TBP and it is likely an obligate heterodimer.

**Codebook experiment summary**: No SMS experiment. ChIP-seq did not pass quality control. All other experiment types were performed but none of them yielded specific PWMs.

| **BRF2** | **ChIP** | **GHT** | **HT** | **SMS** | **PBM** | **Lysate** | **eGFP-IVT** | **IVT** |
| --- | --- | --- | --- | --- | --- | --- | --- | --- |
| **Tested?** | TRUE | TRUE | TRUE | FALSE | TRUE | TRUE | TRUE | TRUE |

**DR1 (Unknown) is a potential TF.** This protein was not annotated to have any likely DBDs. The main source of evidence for a role as a TF is crystal structure PDB:1JFI^242^, showing it as an obligate heterodimer with DRAP1; together they bind TBP to inhibit its function. DR1 and DRAP1 make interactions with DNA but binding could be dependent on TBP1. DRAP1 produced AT-rich motif in external ChIP-seq that is consistent with this function

**Codebook experiment summary**: No SMS experiment. Our ChIP-seq shows enrichment of “TATAAA” motif consistently with it binding together with TBP1 (and possibly DRAP1). No other experiment types enrich in clear motifs.

| **DR1** | **ChIP** | **GHT** | **HT** | **SMS** | **PBM** | **Lysate** | **eGFP-IVT** | **IVT** |
| --- | --- | --- | --- | --- | --- | --- | --- | --- |
| **Tested?** | TRUE | TRUE | TRUE | FALSE | TRUE | TRUE | TRUE | TRUE |

**PHF19 (Unknown) is likely a sequence specific TF.** This protein was not annotated to have any likely DBDs. A recent study shows with PBM that it bound a CG-rich PWM and also that its homologs PHF1 and MTF2 bound DNA specifically with a WH domain^48^. This protein is also known to bind trimethylated Lys-36 of histone H3.

**Codebook experiment summary**: No eGFP-IVT based SELEX or SMS experiments. ChIP-seq is enriched in CG-rich sequences All other experiment types were performed but none of them yielded specific PWMs.

| **PHF19** | **ChIP** | **GHT** | **HT** | **SMS** | **PBM** | **Lysate** | **eGFP-IVT** | **IVT** |
| --- | --- | --- | --- | --- | --- | --- | --- | --- |
| **Tested?** | TRUE | TRUE | TRUE | FALSE | TRUE | TRUE | FALSE | TRUE |

**PURG (Unknown) is a potential TF.** This protein was not annotated to have any likely DBDs. Its paralog PURB was successful with PBM, and paralog PURA has a Transfac PWM. An existing cell extract EMSA based results indicated binding to GGA repeats^244^, but this is discrepant with our PBM data for paralog PURB. These proteins have been also linked on binding single stranded DNA^245^.

**Codebook experiment summary**: No Lysate and eGFP-IVT based SELEX or SMS experiments. ChIP-seq did not pass quality control. Other experiment types did not enriched in anything.

| **PURG** | **ChIP** | **GHT** | **HT** | **SMS** | **PBM** | **Lysate** | **eGFP-IVT** | **IVT** |
| --- | --- | --- | --- | --- | --- | --- | --- | --- |
| **Tested?** | TRUE | TRUE | TRUE | FALSE | TRUE | FALSE | FALSE | TRUE |

## Section 1.2 **SUPPLEMENTARY TABLE TITLES AND LEGENDS**

**Supplementary Table 1**

Codebook proteins and positive control TFs that were analyzed in the Codebook studies along with metadata and information on whether they showed sequence-specific DNA binding activities in different types of experiments, together with the ID of the representative PWM selected in this study, if any.

**Supplementary Table 2**

Amino acid sequence and type (full-length or DBD) for the 716 inserts used in the Codebook studies.

**Supplementary Table 3**

Plasmid backbone and insert for each of the 1,387 plasmids used in the Codebook studies.

**Supplementary Table 4**

The 4,805 experiments performed on Codebook and control TFs, along with 20 GFP control experiments, 43 reanalyzed experiments from Schmitges et al. 2016^8^, and 25 reanalyzed experiments from Isakova et al. 2017^7^. Each experiment is mapped to its ID in an accompanying manuscript^12^ and 9 additional experiments used only in an accompanying manuscript^12^ are marked.

**Supplementary Table 5**

Logo representations for the PWMs that were manually selected as the representative for each of the TFs (i.e. the expert-curated motifs).

**Supplementary Table 6**

Re-assessment outcomes for the unsuccessful proteins. Further details can be found in the **Supplementary Discussion.**

**Supplementary Table 7**

External ChIP-seq datasets obtained from the GTRD database and ENCODE consortium for Codebook and control TFs as well as other putative TFs that were not tested in Codebook.

**Supplementary Table 8**

Transcription factor allele-specific binding sites (ASBs) identified using Codebook data at 5% FDR. Each entry represents an allele specific interaction between a TF and its DNA binding site in the vicinity of, or directly encompassing, the SNV, for which the allelic imbalance of read counts was observed between the reference and the alternative alleles.

**Supplementary Table 9**

The joint set of ASBs (FDR 5%) aggregated across all Codebook data (all transcription factors and experiment types), SNP-associated phenotypes (EBI GWAS Catalog), and known eQTL or ASB annotations of the respective SNVs. Each entry corresponds to the allele-specific binding of one or more TFs. The aggregated statistics combine evidence from multiple TFs and experiments (ChIP-seq and multiple cycles of GHT-SELEX).

**Supplementary Table 10**

Representative motifs for Codebook TFs, TFs with previously known specificities, and motifs for 33 uncharacterized TFs curated from other publications (see “**Curation of new external motifs for previously uncharacterized TFs**”). Motif cluster membership is also indicated (**Extended Data Fig. 6**). The PWMs can be found in **Supplementary Data 1**.

**Supplementary Table 11**

Details of the 653 motif clusters used for the motif activity response analysis (MARA) using FANTOM5 data.

**Supplementary Table 12**

MARA-predicted motif activity estimates across FANTOM5 samples for each motif cluster, their Pearson correlations with the expression of corresponding TFs in 583 samples used for the MARA analysis, ANOVA statistics, and significance estimates.

**Supplementary Table 13**

PWM identifiers, manual curation and other metadata for external motifs available from the JASPAR, HOCOMOCO and Factorbook databases.

**Supplementary Table 14**

Updated census of human transcription factors and their motif coverage.

**Supplementary Table 15**

Success of motif derivation tools across experiment types.

**Supplementary Table 16**

Motifs that represent artifactual or false positive signals that were commonly observed in the Codebook experiments.

**Supplementary Data 1**

Representative motifs for all human TFs. Motif annotation is provided in

## Section 1.3 **Supplementary information references**

**REFERENCES**

87	Singh, U. & Westermark, B. CGGBP1--an indispensable protein with ubiquitous cytoprotective functions. *Ups J Med Sci* **120**, 219–232 (2015). https://doi.org/10.3109/03009734.2015.1086451

88	Yellan, I., Yang, A. W. H. & Hughes, T. R. Diverse Eukaryotic CGG-Binding Proteins Produced by Independent Domestications of hAT Transposons. *Mol Biol Evol* **38**, 2070–2075 (2021). https://doi.org/10.1093/molbev/msab007

89	Aoki, T., Sarkeshik, A., Yates, J. & Schedl, P. Elba, a novel developmentally regulated chromatin boundary factor is a hetero-tripartite DNA binding complex. *eLife* **1**, e00171 (2012). https://doi.org/10.7554/eLife.00171

90	Dai, Q. *et al.* The BEN domain is a novel sequence-specific DNA-binding domain conserved in neural transcriptional repressors. *Genes Dev* **27**, 602–614 (2013). https://doi.org/10.1101/gad.213314.113

91	Zheng, L. *et al.* Distinct structural bases for sequence-specific DNA binding by mammalian BEN domain proteins. *Genes Dev* **36**, 225–240 (2022). https://doi.org/10.1101/gad.348993.121

92	Vetrini, F. *et al.* De novo and inherited TCF20 pathogenic variants are associated with intellectual disability, dysmorphic features, hypotonia, and neurological impairments with similarities to Smith-Magenis syndrome. *Genome Med* **11**, 12 (2019). https://doi.org/10.1186/s13073-019-0623-0

93	Gupta, M., Zak, R., Libermann, T. A. & Gupta, M. P. Tissue-restricted expression of the cardiac alpha-myosin heavy chain gene is controlled by a downstream repressor element containing a palindrome of two ets-binding sites. *Mol Cell Biol* **18**, 7243–7258 (1998). https://doi.org/10.1128/MCB.18.12.7243

94	Zhou, J. *et al.* Attenuation of Forkhead signaling by the retinal determination factor DACH1. *Proc Natl Acad Sci U S A* **107**, 6864–6869 (2010). https://doi.org/10.1073/pnas.1002746107

95	Nicol, R. & Stavnezer, E. Transcriptional repression by v-Ski and c-Ski mediated by a specific DNA binding site. *J Biol Chem* **273**, 3588–3597 (1998). https://doi.org/10.1074/jbc.273.6.3588

96	Chen, R., Amoui, M., Zhang, Z. & Mardon, G. Dachshund and eyes absent proteins form a complex and function synergistically to induce ectopic eye development in Drosophila. *Cell* **91**, 893–903 (1997). https://doi.org/10.1016/s0092-8674(00)80481-x

97	Rass, M. *et al.* The Drosophila functional Smad suppressing element fuss, a homologue of the human Skor genes, retains pro-oncogenic properties of the Ski/Sno family. *PLoS One* **17**, e0262360 (2022). https://doi.org/10.1371/journal.pone.0262360

98	Wang, B., Harrison, W., Overbeek, P. A. & Zheng, H. Transposon mutagenesis with coat color genotyping identifies an essential role for Skor2 in sonic hedgehog signaling and cerebellum development. *Development* **138**, 4487–4497 (2011). https://doi.org/10.1242/dev.067264

99	Hoverter, N. P. *et al.* The TCF C-clamp DNA binding domain expands the Wnt transcriptome via alternative target recognition. *Nucleic Acids Res* **42**, 13615–13632 (2014). https://doi.org/10.1093/nar/gku1186

100	Duan, B. *et al.* Selective Nonmethylated CpG DNA Recognition Mechanism of Cysteine Clamp Domains. *J Am Chem Soc* **143**, 7688–7697 (2021). https://doi.org/10.1021/jacs.1c00599

101	Tanaka, K., Shouguchi-Miyata, J., Miyamoto, N. & Ikeda, J. E. Novel nuclear shuttle proteins, HDBP1 and HDBP2, bind to neuronal cell-specific cis-regulatory element in the promoter for the human Huntington's disease gene. *J Biol Chem* **279**, 7275–7286 (2004). https://doi.org/10.1074/jbc.M310726200

102	Blum, M. *et al.* InterPro: the protein sequence classification resource in 2025. *Nucleic Acids Res* **53**, D444–D456 (2025). https://doi.org/10.1093/nar/gkae1082

103	Abramson, J. *et al.* Accurate structure prediction of biomolecular interactions with AlphaFold 3. *Nature* (2024). https://doi.org/10.1038/s41586-024-07487-w

104	Letunic, I. & Bork, P. SMART v10: three decades of the protein domain annotation resource. *Nucleic Acids Res* (2025). https://doi.org/10.1093/nar/gkaf1023

105	Dyer, S. C. *et al.* Ensembl 2025. *Nucleic Acids Res* **53**, D948–D957 (2025). https://doi.org/10.1093/nar/gkae1071

106	Archbold, H. C., Broussard, C., Chang, M. V. & Cadigan, K. M. Bipartite recognition of DNA by TCF/Pangolin is remarkably flexible and contributes to transcriptional responsiveness and tissue specificity of wingless signaling. *PLoS Genet* **10**, e1004591 (2014). https://doi.org/10.1371/journal.pgen.1004591

107	Bhambhani, C. *et al.* Distinct DNA binding sites contribute to the TCF transcriptional switch in C. elegans and Drosophila. *PLoS Genet* **10**, e1004133 (2014). https://doi.org/10.1371/journal.pgen.1004133

108	Feschotte, C. & Pritham, E. J. DNA transposons and the evolution of eukaryotic genomes. *Annu Rev Genet* **41**, 331–368 (2007). https://doi.org/10.1146/annurev.genet.40.110405.090448

109	Hayward, A., Ghazal, A., Andersson, G., Andersson, L. & Jern, P. ZBED evolution: repeated utilization of DNA transposons as regulators of diverse host functions. *PLoS One* **8**, e59940 (2013). https://doi.org/10.1371/journal.pone.0059940

110	Smit, A. F. & Riggs, A. D. Tiggers and DNA transposon fossils in the human genome. *Proc Natl Acad Sci U S A* **93**, 1443–1448 (1996). https://doi.org/10.1073/pnas.93.4.1443

111	Etchegaray, E., Baas, D., Naville, M., Haftek-Terreau, Z. & Volff, J. N. The neurodevelopmental gene MSANTD2 belongs to a gene family formed by recurrent molecular domestication of Harbinger transposons at the base of vertebrates. *Mol Biol Evol* **39** (2022). https://doi.org/10.1093/molbev/msac173

112	Marquez, C. P. & Pritham, E. J. Phantom, a new subclass of Mutator DNA transposons found in insect viruses and widely distributed in animals. *Genetics* **185**, 1507–1517 (2010). https://doi.org/10.1534/genetics.110.116673

113	Moore, T. *et al.* Polymorphism analysis of JRK/JH8, the human homologue of mouse jerky, and description of a rare mutation in a case of CAE evolving to JME. *Epilepsy Res* **46**, 157–167 (2001). https://doi.org/10.1016/s0920-1211(01)00275-3

114	Pangon, L. *et al.* JRK is a positive regulator of beta-catenin transcriptional activity commonly overexpressed in colon, breast and ovarian cancer. *Oncogene* **35**, 2834–2841 (2016). https://doi.org/10.1038/onc.2015.347

115	Yin, L., Yan, J., Wang, Y. & Sun, Q. TIGD1, a gene of unknown function, involves cell-cycle progression and correlates with poor prognosis in human cancer. *J Cell Biochem* **120**, 9758–9767 (2019). https://doi.org/10.1002/jcb.28256

116	Siegmund, T. & Lehmann, M. The Drosophila Pipsqueak protein defines a new family of helix-turn-helix DNA-binding proteins. *Dev Genes Evol* **212**, 152–157 (2002). https://doi.org/10.1007/s00427-002-0219-2

117	Tanaka, Y. *et al.* Crystal structure of the CENP-B protein-DNA complex: the DNA-binding domains of CENP-B induce kinks in the CENP-B box DNA. *EMBO J* **20**, 6612–6618 (2001). https://doi.org/10.1093/emboj/20.23.6612

118	Cordier, F., Hartmann, B., Rogowski, M., Affolter, M. & Grzesiek, S. DNA recognition by the brinker repressor--an extreme case of coupling between binding and folding. *J Mol Biol* **361**, 659–672 (2006). https://doi.org/10.1016/j.jmb.2006.06.045

119	Storer, J., Hubley, R., Rosen, J., Wheeler, T. J. & Smit, A. F. The Dfam community resource of transposable element families, sequence models, and genome annotations. *Mobile DNA* **12**, 2 (2021). https://doi.org/10.1186/s13100-020-00230-y

120	Kipling, D. & Warburton, P. E. Centromeres, CENP-B and Tigger too. *Trends Genet* **13**, 141–145 (1997). https://doi.org/10.1016/s0168-9525(97)01098-6

121	Cosby, R. L. *et al.* Recurrent evolution of vertebrate transcription factors by transposase capture. *Science* **371** (2021). https://doi.org/10.1126/science.abc6405

122	Markljung, E. *et al.* ZBED6, a novel transcription factor derived from a domesticated DNA transposon regulates IGF2 expression and muscle growth. *PLoS Biol* **7**, e1000256 (2009). https://doi.org/10.1371/journal.pbio.1000256

123	Tipney, H. J. *et al.* Isolation and characterisation of GTF2IRD2, a novel fusion gene and member of the TFII-I family of transcription factors, deleted in Williams-Beuren syndrome. *Eur J Hum Genet* **12**, 551–560 (2004). https://doi.org/10.1038/sj.ejhg.5201174

124	Chen, T. *et al.* Identification of zinc-finger BED domain-containing 3 (Zbed3) as a novel Axin-interacting protein that activates Wnt/beta-catenin signaling. *J Biol Chem* **284**, 6683–6689 (2009). https://doi.org/10.1074/jbc.M807753200

125	Lannes, L., Furman, C. M., Hickman, A. B. & Dyda, F. Zinc-finger BED domains drive the formation of the active Hermes transpososome by asymmetric DNA binding. *Nat Commun* **14**, 4470 (2023). https://doi.org/10.1038/s41467-023-40210-3

126	Casola, C., Lawing, A. M., Betran, E. & Feschotte, C. PIF-like transposons are common in drosophila and have been repeatedly domesticated to generate new host genes. *Mol Biol Evol* **24**, 1872–1888 (2007). https://doi.org/10.1093/molbev/msm116

127	Aravind, L., Anantharaman, V., Balaji, S., Babu, M. M. & Iyer, L. M. The many faces of the helix-turn-helix domain: transcription regulation and beyond. *FEMS Microbiol Rev* **29**, 231–262 (2005). https://doi.org/10.1016/j.femsre.2004.12.008

128	Lambert, S. A. *et al.* Similarity regression predicts evolution of transcription factor sequence specificity. *Nat Genet* **51**, 981–989 (2019). https://doi.org/10.1038/s41588-019-0411-1

129	Sinzelle, L. *et al.* Transposition of a reconstructed Harbinger element in human cells and functional homology with two transposon-derived cellular genes. *Proc Natl Acad Sci U S A* **105**, 4715–4720 (2008). https://doi.org/10.1073/pnas.0707746105

130	Zhang, X., Jiang, N., Feschotte, C. & Wessler, S. R. PIF- and Pong-like transposable elements: distribution, evolution and relationship with Tourist-like miniature inverted-repeat transposable elements. *Genetics* **166**, 971–986 (2004). https://doi.org/10.1093/genetics/166.2.971

131	Almozyan, S., Coulton, J., Babaei-Jadidi, R. & Nateri, A. S. FLYWCH1, a Multi-Functional Zinc Finger Protein Contributes to the DNA Repair Pathway. *Cells* **10** (2021). https://doi.org/10.3390/cells10040889

132	Fullstone, T. L. *et al.* Identification of FLYWCH1 as a regulator of platinum-resistance in epithelial ovarian cancer. *NAR Cancer* **7**, zcaf012 (2025). https://doi.org/10.1093/narcan/zcaf012

133	Muhammad, B. A. *et al.* FLYWCH1, a Novel Suppressor of Nuclear beta-Catenin, Regulates Migration and Morphology in Colorectal Cancer. *Mol Cancer Res* **16**, 1977–1990 (2018). https://doi.org/10.1158/1541-7786.MCR-18-0262

134	Santos-Barriopedro, I., van Mierlo, G. & Vermeulen, M. Off-the-shelf proximity biotinylation for interaction proteomics. *Nat Commun* **12**, 5015 (2021). https://doi.org/10.1038/s41467-021-25338-4

135	Sircoulomb, F. *et al.* ZNF703 gene amplification at 8p12 specifies luminal B breast cancer. *EMBO Mol Med* **3**, 153–166 (2011). https://doi.org/10.1002/emmm.201100121

136	Berkovits-Cymet, H. J., Amann, B. T. & Berg, J. M. Solution structure of a CCHHC domain of neural zinc finger factor-1 and its implications for DNA binding. *Biochemistry* **43**, 898–903 (2004). https://doi.org/10.1021/bi035159d

137	Gamsjaeger, R. *et al.* A structural analysis of DNA binding by myelin transcription factor 1 double zinc fingers. *J Biol Chem* **288**, 35180–35191 (2013). https://doi.org/10.1074/jbc.M113.482075

138	Liu, Z., Yang, X., Tan, F., Cullion, K. & Thiele, C. J. Molecular cloning and characterization of human Castor, a novel human gene upregulated during cell differentiation. *Biochem Biophys Res Commun* **344**, 834–844 (2006). https://doi.org/10.1016/j.bbrc.2006.03.207

139	Aravind, L. & Landsman, D. AT-hook motifs identified in a wide variety of DNA-binding proteins. *Nucleic Acids Res* **26**, 4413–4421 (1998). https://doi.org/10.1093/nar/26.19.4413

140	Carrion, A. M., Link, W. A., Ledo, F., Mellstrom, B. & Naranjo, J. R. DREAM is a Ca2+-regulated transcriptional repressor. *Nature* **398**, 80–84 (1999). https://doi.org/10.1038/18044

141	Rhodes, K. J. *et al.* KChIPs and Kv4 alpha subunits as integral components of A-type potassium channels in mammalian brain. *J Neurosci* **24**, 7903–7915 (2004). https://doi.org/10.1523/JNEUROSCI.0776-04.2004

142	Lei, C. *et al.* Isolation and characterization of a novel zinc finger gene, ZNFD, activating AP1(PMA) transcriptional activities. *Mol Cell Biochem* **340**, 63–71 (2010). https://doi.org/10.1007/s11010-010-0401-1

143	Mazin, A., Milot, E., Devoret, R. & Chartrand, P. KIN17, a mouse nuclear protein, binds to bent DNA fragments that are found at illegitimate recombination junctions in mammalian cells. *Mol Gen Genet* **244**, 435–438 (1994). https://doi.org/10.1007/BF00286696

144	Li, W., Zhang, J. & Liu, Q. Kin17 promotes rDNA transcription, ribosomal biogenesis, and cortical lamination. *EMBO Rep* **26**, 4283–4311 (2025). https://doi.org/10.1038/s44319-025-00524-3

145	Huang, X. *et al.* Roles and regulatory mechanisms of KIN17 in cancers (Review). *Oncol Lett* **25**, 137 (2023). https://doi.org/10.3892/ol.2023.13723

146	Sanada, T. *et al.* FLN29 deficiency reveals its negative regulatory role in the Toll-like receptor (TLR) and retinoic acid-inducible gene I (RIG-I)-like helicase signaling pathway. *J Biol Chem* **283**, 33858–33864 (2008). https://doi.org/10.1074/jbc.M806923200

147	Close, P. *et al.* DBIRD complex integrates alternative mRNA splicing with RNA polymerase II transcript elongation. *Nature* **484**, 386–389 (2012). https://doi.org/10.1038/nature10925

148	Wang, C., Low, W. C., Liu, A. & Wang, B. Centrosomal protein DZIP1 regulates Hedgehog signaling by promoting cytoplasmic retention of transcription factor GLI3 and affecting ciliogenesis. *J Biol Chem* **288**, 29518–29529 (2013). https://doi.org/10.1074/jbc.M113.492066

149	Mishra, A., Eathiraj, S., Corvera, S. & Lambright, D. G. Structural basis for Rab GTPase recognition and endosome tethering by the C2H2 zinc finger of Early Endosomal Autoantigen 1 (EEA1). *Proc Natl Acad Sci U S A* **107**, 10866–10871 (2010). https://doi.org/10.1073/pnas.1000843107

150	Xu, T. *et al.* RBPJ/CBF1 interacts with L3MBTL3/MBT1 to promote repression of Notch signaling via histone demethylase KDM1A/LSD1. *EMBO J* **36**, 3232–3249 (2017). https://doi.org/10.15252/embj.201796525

151	Huang, J. *et al.* PRMT3 and CARM1: Emerging Epigenetic Targets in Cancer. *J Cell Mol Med* **29**, e70386 (2025). https://doi.org/10.1111/jcmm.70386

152	Frankel, A. & Clarke, S. PRMT3 is a distinct member of the protein arginine N-methyltransferase family. Conferral of substrate specificity by a zinc-finger domain. *J Biol Chem* **275**, 32974–32982 (2000). https://doi.org/10.1074/jbc.M006445200

153	Jiang, N. *et al.* Characterization of dsRNA binding proteins through solubility analysis identifies ZNF385A as a dsRNA homeostasis regulator. *Nature communications* **16**, 3433 (2025). https://doi.org/10.1038/s41467-025-58704-7

154	Sharma, S., Dimasi, D., Higginson, K. & Della, N. G. RZF, a zinc-finger protein in the photoreceptors of human retina. *Gene* **342**, 219–229 (2004). https://doi.org/10.1016/j.gene.2004.08.015

155	Chapman, R. M. *et al.* Convergent Evidence That ZNF804A Is a Regulator of Pre-messenger RNA Processing and Gene Expression. *Schizophr Bull* **45**, 1267–1278 (2019). https://doi.org/10.1093/schbul/sby183

156	Klockmeier, K., Silva Ramos, E., Rasko, T., Marti Pastor, A. & Wanker, E. E. Schizophrenia risk candidate protein ZNF804A interacts with STAT2 and influences interferon-mediated gene transcription in mammalian cells. *J Mol Biol* **433**, 167184 (2021). https://doi.org/10.1016/j.jmb.2021.167184

157	Toledo, C. M. *et al.* BuGZ is required for Bub3 stability, Bub1 kinetochore function, and chromosome alignment. *Dev Cell* **28**, 282–294 (2014). https://doi.org/10.1016/j.devcel.2013.12.014

158	Jiang, H. *et al.* Phase transition of spindle-associated protein regulate spindle apparatus assembly. *Cell* **163**, 108–122 (2015). https://doi.org/10.1016/j.cell.2015.08.010

159	Pioli, P. D., Debnath, I., Weis, J. J. & Weis, J. H. Zfp318 regulates IgD expression by abrogating transcription termination within the Ighm/Ighd locus. *Journal of immunology* **193**, 2546–2553 (2014). https://doi.org/10.4049/jimmunol.1401275

160	Hao, W. *et al.* ZUP1 is a key component of the MAVS complex and acts as a protector of host against viral invasion. *FASEB journal : official publication of the Federation of American Societies for Experimental Biology* **39**, e70419 (2025). https://doi.org/10.1096/fj.202401661RRR

161	Marstad, A. *et al.* A-kinase anchoring protein AKAP95 is a novel regulator of ribosomal RNA synthesis. *FEBS J* **283**, 757–770 (2016). https://doi.org/10.1111/febs.13630

162	Hu, X. *et al.* The RNA-binding protein AKAP8 suppresses tumor metastasis by antagonizing EMT-associated alternative splicing. *Nature communications* **11**, 486 (2020). https://doi.org/10.1038/s41467-020-14304-1

163	Bieluszewska, A., Weglewska, M., Bieluszewski, T., Lesniewicz, K. & Poreba, E. PKA-binding domain of AKAP8 is essential for direct interaction with DPY30 protein. *FEBS J* **285**, 947–964 (2018). https://doi.org/10.1111/febs.14378

164	Leeb, M., Dietmann, S., Paramor, M., Niwa, H. & Smith, A. Genetic exploration of the exit from self-renewal using haploid embryonic stem cells. *Cell stem cell* **14**, 385–393 (2014). https://doi.org/10.1016/j.stem.2013.12.008

165	Boxer, L. D., Barajas, B., Tao, S., Zhang, J. & Khavari, P. A. ZNF750 interacts with KLF4 and RCOR1, KDM1A, and CTBP1/2 chromatin regulators to repress epidermal progenitor genes and induce differentiation genes. *Genes Dev* **28**, 2013–2026 (2014). https://doi.org/10.1101/gad.246579.114

166	Sen, G. L. *et al.* ZNF750 is a p63 target gene that induces KLF4 to drive terminal epidermal differentiation. *Dev Cell* **22**, 669–677 (2012). https://doi.org/10.1016/j.devcel.2011.12.001

167	Zhang, Y., Park, E., Kim, C. S. & Paik, J. H. ZNF365 promotes stalled replication forks recovery to maintain genome stability. *Cell cycle* **12**, 2817–2828 (2013). https://doi.org/10.4161/cc.25882

168	Lee, S., Ye, A. & Kim, J. DNA-Binding Motif of the Imprinted Transcription Factor PEG3. *PLoS One* **10**, e0145531 (2015). https://doi.org/10.1371/journal.pone.0145531

169	Thiaville, M. M. *et al.* DNA-binding motif and target genes of the imprinted transcription factor PEG3. *Gene* **512**, 314–320 (2013). https://doi.org/10.1016/j.gene.2012.10.005

170	Orouji, E., Peitsch, W. K., Orouji, A., Houben, R. & Utikal, J. Unique Role of Histone Methyltransferase PRDM8 in the Tumorigenesis of Virus-Negative Merkel Cell Carcinoma. *Cancers (Basel)* **12** (2020). https://doi.org/10.3390/cancers12041057

171	Fox, A. H. *et al.* Transcriptional cofactors of the FOG family interact with GATA proteins by means of multiple zinc fingers. *EMBO J* **18**, 2812–2822 (1999). https://doi.org/10.1093/emboj/18.10.2812

172	Loesch, R., Chenane, L. & Colnot, S. ARID2 Chromatin Remodeler in Hepatocellular Carcinoma. *Cells* **9** (2020). https://doi.org/10.3390/cells9102152

173	Yuan, J., Chen, K., Zhang, W. & Chen, Z. Structure of human chromatin-remodelling PBAF complex bound to a nucleosome. *Nature* **605**, 166–171 (2022). https://doi.org/10.1038/s41586-022-04658-5

174	Siddiqa, A. *et al.* Regulation of CD40 and CD40 ligand by the AT-hook transcription factor AKNA. *Nature* **410**, 383–387 (2001). https://doi.org/10.1038/35066602

175	Hart, D., Rodriguez Gutierrez, D. & Biason-Lauber, A. CBX2 in DSD: The Quirky Kid on the Block. *Sex Dev* **16**, 162–170 (2022). https://doi.org/10.1159/000522164

176	Yao, T. *et al.* Structural basis of the crosstalk between histone H2B monoubiquitination and H3 lysine 79 methylation on nucleosome. *Cell Res* **29**, 330–333 (2019). https://doi.org/10.1038/s41422-019-0146-7

177	Van, H. T. *et al.* Methyl-lysine readers PHF20 and PHF20L1 define two distinct gene expression-regulating NSL complexes. *J Biol Chem* **298**, 101588 (2022). https://doi.org/10.1016/j.jbc.2022.101588

178	Nguyen, A. L., Smith, E. M. & Cheeseman, I. M. Co-essentiality analysis identifies PRR12 as a cohesin interacting protein and contributor to genomic integrity. *Dev Cell* **60**, 1217–1233 e1217 (2025). https://doi.org/10.1016/j.devcel.2024.12.015

179	Hwang, E. S., Choi, A. & Ho, I. C. Transcriptional regulation of GATA-3 by an intronic regulatory region and fetal liver zinc finger protein 1. *Journal of immunology* **169**, 248–253 (2002). https://doi.org/10.4049/jimmunol.169.1.248

180	Jolma, A. *et al.* Binding specificities of human RNA-binding proteins toward structured and linear RNA sequences. *Genome Res* **30**, 962–973 (2020). https://doi.org/10.1101/gr.258848.119

181	Li, R. *et al.* ZIP: a novel transcription repressor, represses EGFR oncogene and suppresses breast carcinogenesis. *EMBO J* **28**, 2763–2776 (2009). https://doi.org/10.1038/emboj.2009.211

182	Barkess, G. *et al.* The chromatin-binding protein HMGN3 stimulates histone acetylation and transcription across the Glyt1 gene. *Biochem J* **442**, 495–505 (2012). https://doi.org/10.1042/BJ20111502

183	Chen, S. *et al.* Structural basis of the TAM domain of BAZ2A in binding to DNA or RNA independent of methylation status. *J Biol Chem* **297**, 101351 (2021). https://doi.org/10.1016/j.jbc.2021.101351

184	Sabogal, A., Lyubimov, A. Y., Corn, J. E., Berger, J. M. & Rio, D. C. THAP proteins target specific DNA sites through bipartite recognition of adjacent major and minor grooves. *Nat Struct Mol Biol* **17**, 117–123 (2010). https://doi.org/10.1038/nsmb.1742

185	Campagne, S., Saurel, O., Gervais, V. & Milon, A. Structural determinants of specific DNA-recognition by the THAP zinc finger. *Nucleic Acids Res* **38**, 3466–3476 (2010). https://doi.org/10.1093/nar/gkq053

186	Toyoda, S. *et al.* Sohlh2 affects differentiation of KIT positive oocytes and spermatogonia. *Dev Biol* **325**, 238–248 (2009). https://doi.org/10.1016/j.ydbio.2008.10.019

187	Lazebnik, M. B., Tussie-Luna, M. I. & Roy, A. L. Determination and functional analysis of the consensus binding site for TFII-I family member BEN, implicated in Williams-Beuren syndrome. *J Biol Chem* **283**, 11078–11082 (2008). https://doi.org/10.1074/jbc.C800049200

188	Ahel, J. *et al.* ChAHP2 and ChAHP control diverse retrotransposons by complementary activities. *Genes Dev* **38**, 554–568 (2024). https://doi.org/10.1101/gad.351769.124

189	Kawata, H. *et al.* Zinc-fingers and homeoboxes (ZHX) 2, a novel member of the ZHX family, functions as a transcriptional repressor. *Biochem J* **373**, 747–757 (2003). https://doi.org/10.1042/BJ20030171

190	Walavalkar, N. M., Cramer, J. M., Buchwald, W. A., Scarsdale, J. N. & Williams, D. C., Jr. Solution structure and intramolecular exchange of methyl-cytosine binding domain protein 4 (MBD4) on DNA suggests a mechanism to scan for mCpG/TpG mismatches. *Nucleic Acids Res* **42**, 11218–11232 (2014). https://doi.org/10.1093/nar/gku782

191	Baymaz, H. I. *et al.* MBD5 and MBD6 interact with the human PR-DUB complex through their methyl-CpG-binding domain. *Proteomics* **14**, 2179–2189 (2014). https://doi.org/10.1002/pmic.201400013

192	Yakubovskaya, E., Mejia, E., Byrnes, J., Hambardjieva, E. & Garcia-Diaz, M. Helix unwinding and base flipping enable human MTERF1 to terminate mitochondrial transcription. *Cell* **141**, 982–993 (2010). https://doi.org/10.1016/j.cell.2010.05.018

193	Shibuya, H., Ishiguro, K. & Watanabe, Y. The TRF1-binding protein TERB1 promotes chromosome movement and telomere rigidity in meiosis. *Nat Cell Biol* **16**, 145–156 (2014). https://doi.org/10.1038/ncb2896

194	Song, Z., Krishna, S., Thanos, D., Strominger, J. L. & Ono, S. J. A novel cysteine-rich sequence-specific DNA-binding protein interacts with the conserved X-box motif of the human major histocompatibility complex class II genes via a repeated Cys-His domain and functions as a transcriptional repressor. *J Exp Med* **180**, 1763–1774 (1994). https://doi.org/10.1084/jem.180.5.1763

195	Nitta, K. R. *et al.* Conservation of transcription factor binding specificities across 600 million years of bilateria evolution. *eLife* **4** (2015). https://doi.org/10.7554/eLife.04837

196	Chintala, S. & Katzenellenbogen, R. A. NFX1, Its Isoforms and Roles in Biology, Disease and Cancer. *Biology (Basel)* **10** (2021). https://doi.org/10.3390/biology10040279

197	Akhtar, W. & Veenstra, G. J. TBP-related factors: a paradigm of diversity in transcription initiation. *Cell & bioscience* **1**, 23 (2011). https://doi.org/10.1186/2045-3701-1-23

198	Chong, J. A. *et al.* TATA-binding protein (TBP)-like factor (TLF) is a functional regulator of transcription: reciprocal regulation of the neurofibromatosis type 1 and c-fos genes by TLF/TRF2 and TBP. *Mol Cell Biol* **25**, 2632–2643 (2005). https://doi.org/10.1128/MCB.25.7.2632-2643.2005

199	Lyons, P. J., Muise, A. M. & Ro, H. S. MAPK modulates the DNA binding of adipocyte enhancer-binding protein 1. *Biochemistry* **44**, 926–931 (2005). https://doi.org/10.1021/bi0480178

200	Ladha, J., Sinha, S., Bhat, V., Donakonda, S. & Rao, S. M. Identification of genomic targets of transcription factor AEBP1 and its role in survival of glioma cells. *Molecular cancer research : MCR* **10**, 1039–1051 (2012). https://doi.org/10.1158/1541-7786.MCR-11-0488

201	LeClerc, S., Palaniswami, R., Xie, B. X. & Govindan, M. V. Molecular cloning and characterization of a factor that binds the human glucocorticoid receptor gene and represses its expression. *J Biol Chem* **266**, 17333–17340 (1991).

202	Levay, M., Settleman, J. & Ligeti, E. Regulation of the substrate preference of p190RhoGAP by protein kinase C-mediated phosphorylation of a phospholipid binding site. *Biochemistry* **48**, 8615–8623 (2009). https://doi.org/10.1021/bi900667y

203	Rogaeva, A. & Albert, P. R. The mental retardation gene CC2D1A/Freud-1 encodes a long isoform that binds conserved DNA elements to repress gene transcription. *Eur J Neurosci* **26**, 965–974 (2007). https://doi.org/10.1111/j.1460-9568.2007.05727.x

204	Yatskevich, S. *et al.* Structure of the human inner kinetochore bound to a centromeric CENP-A nucleosome. *Science* **376**, 844–852 (2022). https://doi.org/10.1126/science.abn3810

205	Liu, H. *et al.* Cloning and functional analysis of FLJ20420: a novel transcription factor for the BAG-1 promoter. *PLoS One* **7**, e34832 (2012). https://doi.org/10.1371/journal.pone.0034832

206	Gingras, S., Pelletier, S., Boyd, K. & Ihle, J. N. Characterization of a family of novel cysteine- serine-rich nuclear proteins (CSRNP). *PLoS One* **2**, e808 (2007). https://doi.org/10.1371/journal.pone.0000808

207	Steffensen, K. R. *et al.* Human NCU-G1 can function as a transcription factor and as a nuclear receptor co-activator. *BMC Mol Biol* **8**, 106 (2007). https://doi.org/10.1186/1471-2199-8-106

208	Hsu, L. C. *et al.* The murine G+C-rich promoter binding protein mGPBP is required for promoter-specific transcription. *Mol Cell Biol* **23**, 8773–8785 (2003). https://doi.org/10.1128/MCB.23.23.8773-8785.2003

209	Filarsky, M. *et al.* The extended AT-hook is a novel RNA binding motif. *RNA Biol* **12**, 864–876 (2015). https://doi.org/10.1080/15476286.2015.1060394

210	Nourbakhsh, M. *et al.* The NF-kappa b repressing factor is involved in basal repression and interleukin (IL)-1-induced activation of IL-8 transcription by binding to a conserved NF-kappa b-flanking sequence element. *J Biol Chem* **276**, 4501–4508 (2001). https://doi.org/10.1074/jbc.M007532200

211	Dreikhausen, U., Hiebenthal-Millow, K., Bartels, M., Resch, K. & Nourbakhsh, M. NF-kappaB-repressing factor inhibits elongation of human immunodeficiency virus type 1 transcription by DRB sensitivity-inducing factor. *Mol Cell Biol* **25**, 7473–7483 (2005). https://doi.org/10.1128/MCB.25.17.7473-7483.2005

212	Chen, X. J. *et al.* Homoharringtonine deregulates MYC transcriptional expression by directly binding NF-kappaB repressing factor. *Proc Natl Acad Sci U S A* **116**, 2220–2225 (2019). https://doi.org/10.1073/pnas.1818539116

213	Lahiri, D. K., Maloney, B., Rogers, J. T. & Ge, Y. W. PuF, an antimetastatic and developmental signaling protein, interacts with the Alzheimer's amyloid-beta precursor protein via a tissue-specific proximal regulatory element (PRE). *BMC Genomics* **14**, 68 (2013). https://doi.org/10.1186/1471-2164-14-68

214	Kar, A. *et al.* Metastases suppressor NME2 associates with telomere ends and telomerase and reduces telomerase activity within cells. *Nucleic Acids Res* **40**, 2554–2565 (2012). https://doi.org/10.1093/nar/gkr1109

215	Dexheimer, T. S. *et al.* NM23-H2 may play an indirect role in transcriptional activation of c-myc gene expression but does not cleave the nuclease hypersensitive element III(1). *Mol Cancer Ther* **8**, 1363–1377 (2009). https://doi.org/10.1158/1535-7163.MCT-08-1093

216	Zhang, Y. & Hamburger, A. W. Heregulin regulates the ability of the ErbB3-binding protein Ebp1 to bind E2F promoter elements and repress E2F-mediated transcription. *J Biol Chem* **279**, 26126–26133 (2004). https://doi.org/10.1074/jbc.M314305200

217	Wild, K. *et al.* MetAP-like Ebp1 occupies the human ribosomal tunnel exit and recruits flexible rRNA expansion segments. *Nature communications* **11**, 776 (2020). https://doi.org/10.1038/s41467-020-14603-7

218	Jia, W. *et al.* PA2G4 in health and disease: An underestimated multifunctional regulator. *J Adv Res* **78**, 307–323 (2025). https://doi.org/10.1016/j.jare.2025.02.002

219	Kanno, M., Hasegawa, M., Ishida, A., Isono, K. & Taniguchi, M. mel-18, a Polycomb group-related mammalian gene, encodes a transcriptional negative regulator with tumor suppressive activity. *EMBO J* **14**, 5672–5678 (1995). https://doi.org/10.1002/j.1460-2075.1995.tb00254.x

220	Scelfo, A. *et al.* Functional Landscape of PCGF Proteins Reveals Both RING1A/B-Dependent-and RING1A/B-Independent-Specific Activities. *Mol Cell* **74**, 1037–1052 e1037 (2019). https://doi.org/10.1016/j.molcel.2019.04.002

221	Zhou, Q. *et al.* Phospholipid scramblase 1 binds to the promoter region of the inositol 1,4,5-triphosphate receptor type 1 gene to enhance its expression. *J Biol Chem* **280**, 35062–35068 (2005). https://doi.org/10.1074/jbc.M504821200

222	Dal Col, J. *et al.* Phospholipid scramblase 1: a protein with multiple functions via multiple molecular interactors. *Cell Commun Signal* **20**, 78 (2022). https://doi.org/10.1186/s12964-022-00895-3

223	Murao, K. *et al.* The transcriptional factor PREB mediates MCP-1 transcription induced by cytokines in human vascular endothelial cells. *Atherosclerosis* **207**, 45–50 (2009). https://doi.org/10.1016/j.atherosclerosis.2009.03.051

224	Tokunaga, C. *et al.* Molecular cloning and characterization of a novel protein kinase C-interacting protein with structural motifs related to RBCC family proteins. *Biochem Biophys Res Commun* **244**, 353–359 (1998). https://doi.org/10.1006/bbrc.1998.8270

225	Montano, M. M., Wittmann, B. M. & Bianco, N. R. Identification and characterization of a novel factor that regulates quinone reductase gene transcriptional activity. *J Biol Chem* **275**, 34306–34313 (2000). https://doi.org/10.1074/jbc.M003880200

226	Renz, A. & Fackelmayer, F. O. Purification and molecular cloning of the scaffold attachment factor B (SAF-B), a novel human nuclear protein that specifically binds to S/MAR-DNA. *Nucleic Acids Res* **24**, 843–849 (1996). https://doi.org/10.1093/nar/24.5.843

227	Hamamoto, R. *et al.* SMYD3 encodes a histone methyltransferase involved in the proliferation of cancer cells. *Nat Cell Biol* **6**, 731–740 (2004). https://doi.org/10.1038/ncb1151

228	Sadowski, C. L., Henry, R. W., Kobayashi, R. & Hernandez, N. The SNAP45 subunit of the small nuclear RNA (snRNA) activating protein complex is required for RNA polymerase II and III snRNA gene transcription and interacts with the TATA box binding protein. *Proc Natl Acad Sci U S A* **93**, 4289–4293 (1996). https://doi.org/10.1073/pnas.93.9.4289

229	Henry, R. W., Mittal, V., Ma, B., Kobayashi, R. & Hernandez, N. SNAP19 mediates the assembly of a functional core promoter complex (SNAPc) shared by RNA polymerases II and III. *Genes Dev* **12**, 2664–2672 (1998). https://doi.org/10.1101/gad.12.17.2664

230	Rengachari, S. *et al.* Structural basis of SNAPc-dependent snRNA transcription initiation by RNA polymerase II. *Nat Struct Mol Biol* **29**, 1159–1169 (2022). https://doi.org/10.1038/s41594-022-00857-w

231	Sun, C. T. *et al.* Transcription repression of human hepatitis B virus genes by negative regulatory element-binding protein/SON. *J Biol Chem* **276**, 24059–24067 (2001). https://doi.org/10.1074/jbc.M101330200

232	Hu, L. *et al.* Crystal structure of TET2-DNA complex: insight into TET-mediated 5mC oxidation. *Cell* **155**, 1545–1555 (2013). https://doi.org/10.1016/j.cell.2013.11.020

233	Wu, X. & Zhang, Y. TET-mediated active DNA demethylation: mechanism, function and beyond. *Nat Rev Genet* **18**, 517–534 (2017). https://doi.org/10.1038/nrg.2017.33

234	Garcia, J. A. *et al.* Cloning and chromosomal mapping of a human immunodeficiency virus 1 "TATA" element modulatory factor. *Proc Natl Acad Sci U S A* **89**, 9372–9376 (1992). https://doi.org/10.1073/pnas.89.20.9372

235	Hsiao, P. W. & Chang, C. Isolation and characterization of ARA160 as the first androgen receptor N-terminal-associated coactivator in human prostate cells. *J Biol Chem* **274**, 22373–22379 (1999). https://doi.org/10.1074/jbc.274.32.22373

236	Yamane, J. *et al.* Functional involvement of TMF/ARA160 in Rab6-dependent retrograde membrane traffic. *Exp Cell Res* **313**, 3472–3485 (2007). https://doi.org/10.1016/j.yexcr.2007.07.010

237	Ohta, S., Shimekake, Y. & Nagata, K. Molecular cloning and characterization of a transcription factor for the C-type natriuretic peptide gene promoter. *Eur J Biochem* **242**, 460–466 (1996). https://doi.org/10.1111/j.1432-1033.1996.460rr.x

238	Kester, H. A., Blanchetot, C., den Hertog, J., van der Saag, P. T. & van der Burg, B. Transforming growth factor-beta-stimulated clone-22 is a member of a family of leucine zipper proteins that can homo- and heterodimerize and has transcriptional repressor activity. *J Biol Chem* **274**, 27439–27447 (1999). https://doi.org/10.1074/jbc.274.39.27439

239	Choi, S. J. *et al.* Tsc-22 enhances TGF-beta signaling by associating with Smad4 and induces erythroid cell differentiation. *Mol Cell Biochem* **271**, 23–28 (2005). https://doi.org/10.1007/s11010-005-3456-7

240	Mo, X., Bailin, T. & Sadofsky, M. J. RAG1 and RAG2 cooperate in specific binding to the recombination signal sequence in vitro. *J Biol Chem* **274**, 7025–7031 (1999). https://doi.org/10.1074/jbc.274.11.7025

241	Zhao, Q. *et al.* The MHF complex senses branched DNA by binding a pair of crossover DNA duplexes. *Nature communications* **5**, 2987 (2014). https://doi.org/10.1038/ncomms3987

242	Kamada, K. *et al.* Crystal structure of negative cofactor 2 recognizing the TBP-DNA transcription complex. *Cell* **106**, 71–81 (2001). https://doi.org/10.1016/s0092-8674(01)00417-2

243	Gouge, J. *et al.* Redox Signaling by the RNA Polymerase III TFIIB-Related Factor Brf2. *Cell* **163**, 1375–1387 (2015). https://doi.org/10.1016/j.cell.2015.11.005

244	Subramanian, S. V. *et al.* Induction of vascular smooth muscle alpha-actin gene transcription in transforming growth factor beta1-activated myofibroblasts mediated by dynamic interplay between the Pur repressor proteins and Sp1/Smad coactivators. *Mol Biol Cell* **15**, 4532–4543 (2004). https://doi.org/10.1091/mbc.e04-04-0348

245	Bergemann, A. D., Ma, Z. W. & Johnson, E. M. Sequence of cDNA comprising the human pur gene and sequence-specific single-stranded-DNA-binding properties of the encoded protein. *Mol Cell Biol* **12**, 5673–5682 (1992). https://doi.org/10.1128/mcb.12.12.5673-5682.1992
