# Supplementary material for: An expanded codebook of human transcription factor DNA-binding specificity
Source: Nature. 2026 Aug 5;657(8130):275–83. doi: 10.1038/s41586-026-10798-9 (PMC13538051; doi:10.1038/s41586-026-10798-9)
Supplement: Supplementary file 4 [file 41586_2026_10798_MOESM3_ESM.zip › 2024-11-25439B-s3/Supplementary_tables_guide.docx]

**An expanded codebook of human transcription factor DNA binding specificity**

Arttu Jolma**^1,15^**, Kaitlin U. Laverty**^1,2,3,15^**, Ali Fathi**^1,3,15^**, Ally W.H. Yang**^1,15^**, Isaac Yellan**^1,3,15^**, Ilya E. Vorontsov**^4,15^**, Antoni J. Gralak**^5,6^**, Judith F. Kribelbauer-Swietek**^5,6,7^**, Sachi Inukai**^5,6^**, Rozita Razavi**^1^**, Mihai Albu**^1^**, Alexander Brechalov**^1^**, Zain M. Patel**^1,3^**, Vladimir Nozdrin**^8^**, Georgy Meshcheryakov**^9,10^**, Andrey Buyan**^9,10^**, Ivan Kozin**^4,8,9^**, Sergey Abramov**^4,11^**, Alexandr Boytsov**^4,11^**, The Codebook Consortium*, Quaid D. Morris^2^, Matthew T. Weirauch**^12^**, Oriol Fornes**^13^**, Vsevolod J. Makeev**^4,#^**, Jan Grau**^14^**, Ivo Grosse**^14^**, Philipp Bucher**^6^**, Bart Deplancke**^5,6^****, Ivan V. Kulakovskiy**^4,9,10^****, and Timothy R. Hughes**^1,3^****

**^1^**Donnelly Centre, University of Toronto, Toronto, ON M5S 3E1, Canada

**^2^**Computational and Systems Biology Program, Sloan Kettering Institute, Memorial Sloan Kettering Cancer Center, New York, NY 10065, USA

**^3^**Department of Molecular Genetics, University of Toronto, Toronto, ON M5S 1A8, Canada

**^4^**Vavilov Institute of General Genetics, Russian Academy of Sciences, 119991, Moscow, Russia

**^5^**Laboratory of Systems Biology and Genetics, Institute of Bioengineering, School of Life Sciences, École Polytechnique Fédérale de Lausanne, 1015, Lausanne, Switzerland

**^6^**Swiss Institute of Bioinformatics, 1015, Lausanne, Switzerland

**^7^**Department of Biological Sciences, University of Southern California, Los Angeles, CA, USA

**^8^**Faculty of Bioengineering and Bioinformatics, Lomonosov Moscow State University, 119991, Moscow, Russia

**^9^**Institute of Protein Research, Russian Academy of Sciences, 142290, Pushchino, Russia

**^10^**Institute of Biochemistry and Genetics, Ufa Federal Research Centre of Russian Academy of Sciences, 450054, Ufa, Russia

**^11^**Altius Institute for Biomedical Sciences, Seattle, WA 98121, USA

**^12^**Center for Autoimmune Genomics and Etiology, Cincinnati Children’s Hospital Medical Center, Cincinnati, OH, USA; Divisions of Allergy and Immunology, Biomedical Informatics, Human Genetics, and Developmental Biology, Cincinnati Children’s Hospital Medical Center, Cincinnati, OH, USA; Department of Pediatrics, University of Cincinnati College of Medicine, Cincinnati, OH, USA

**^13^**Department of Medical Genetics, Centre for Molecular Medicine and Therapeutics, BC Children’s Hospital Research Institute, University of British Columbia, Vancouver, BC V5Z 4H4, Canada

**^14^**Institute of Computer Science, Martin Luther University Halle-Wittenberg, 06099, Halle, Germany

**^15^**These authors contributed equally

*A list of authors and their affiliations appears at the end of the paper.

**^#^**Present address: Cancer Research UK National Biomarker Centre, University of Manchester, Manchester, M20 4BX, UK

**To whom correspondence should be addressed: [bart.deplancke@epfl.ch](mailto:bart.deplancke@epfl.ch), ivan.kulakovskiy@gmail.com, t.hughes@utoronto.ca

# Supplementary Information Guide

**Supplementary Table 1**

Codebook proteins and positive control TFs that were analyzed in the Codebook studies along with metadata and information on whether they showed sequence-specific DNA binding activities in different types of experiments, together with the ID of the representative PWM selected in this study, if any.

**Supplementary Table 2**

Amino acid sequence and type (full-length or DBD) for the 716 inserts used in the Codebook studies.

**Supplementary Table 3**

Plasmid backbone and insert for each of the 1,387 plasmids used in the Codebook studies.

**Supplementary Table 4**

The 4,805 experiments performed on Codebook and control TFs, along with 20 GFP control experiments, 43 reanalyzed experiments from Schmitges et al. 2016^8^, and 25 reanalyzed experiments from Isakova et al. 2017^7^. Each experiment is mapped to its ID in an accompanying manuscript^12^, and 9 additional experiments used only in an accompanying manuscript^12^ are marked.

**Supplementary Table 5**

Logo representations for the PWMs that were manually selected as the representative for each of the TFs (i.e. the expert-curated motifs).

**Supplementary Table 6**

Re-assessment outcomes for the unsuccessful proteins. Further details can be found in the **Supplementary Discussion.**

**Supplementary Table 7**

External ChIP-seq datasets obtained from the GTRD database and ENCODE consortium for Codebook and control TFs as well as other putative TFs that were not tested in Codebook.

**Supplementary Table 8**

Transcription factor allele-specific binding sites (ASBs) identified using Codebook data at 5% FDR. Each entry represents an allele specific interaction between a TF and its DNA binding site in the vicinity of, or directly encompassing, the SNV, for which the allelic imbalance of read counts was observed between the reference and the alternative alleles.

**Supplementary Table 9**

The joint set of ASBs (FDR 5%) aggregated across all Codebook data (all transcription factors and experiment types), SNP-associated phenotypes (EBI GWAS Catalog), and known eQTL or ASB annotations of the respective SNVs. Each entry corresponds to the allele-specific binding of one or more TFs. The aggregated statistics combine evidence from multiple TFs and experiments (ChIP-seq and multiple cycles of GHT-SELEX).

**Supplementary Table 10**

Representative motifs for Codebook TFs, TFs with previously known specificities, and motifs for 33 uncharacterized TFs curated from other publications (see “**Curation of new external motifs for previously uncharacterized TFs**”). Motif cluster membership is also indicated (**Extended Data Fig. 6**). The PWMs can be found in **Supplementary Data 1**.

**Supplementary Table 11**

Details of the 653 motif clusters used for the motif activity response analysis (MARA) using FANTOM5 data.

**Supplementary Table 12**

MARA-predicted motif activity estimates across FANTOM5 samples for each motif cluster, their Pearson correlations with the expression of corresponding TFs in 583 samples used for the MARA analysis, ANOVA statistics, and significance estimates.

**Supplementary Table 13**

PWM identifiers, manual curation and other metadata for external motifs available from the JASPAR, HOCOMOCO and Factorbook databases.

**Supplementary Table 14**

Updated census of human transcription factors and their motif coverage.

**Supplementary Table 15**

Success of motif derivation tools across experiment types.

**Supplementary Table 16**

Motifs that represent artifactual or false positive signals that were commonly observed in the Codebook experiments.
