# Supplementary material for: An expanded codebook of human transcription factor DNA-binding specificity
Source: Nature. 2026 Aug 5;657(8130):275–83. doi: 10.1038/s41586-026-10798-9 (PMC13538051; doi:10.1038/s41586-026-10798-9)
Supplement: Supplementary file 4 [file 41586_2026_10798_MOESM3_ESM.zip › 2024-11-25439B-s3/SupplementaryTable5_PWMs.pdf]

TF	Type	DBD type(s)	Motif source	Motif derivation algorithm	Motif
YY1	Control	C2H2 ZF	ChIP-seq	MEME	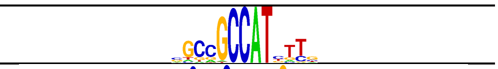
CTCF	Control	C2H2 ZF	GHT-SELEX (Lysate)	Homer	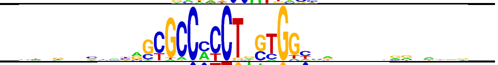
GLI4	Control	C2H2 ZF	GHT-SELEX (Lysate)	ExplaiNN	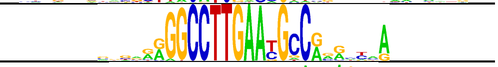
ZFP3	Control	C2H2 ZF	ChIP-seq	RCade	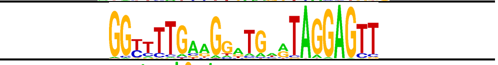
ZIM3	Control	C2H2 ZF	ChIP-seq	RCade	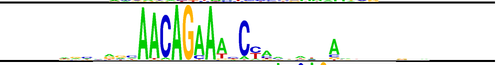
ZNF134	Control	C2H2 ZF	GHT-SELEX (Lysate)	RCade	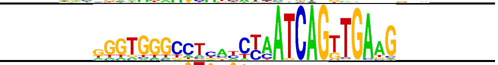
ZNF18	Control	C2H2 ZF	HT-SELEX (Lysate)	Autoseed	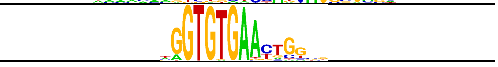
ZNF260	Control	C2H2 ZF	GHT-SELEX (Lysate)	Dimont	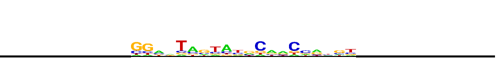
ZNF322	Control	C2H2 ZF	ChIP-seq	ChIPMunk	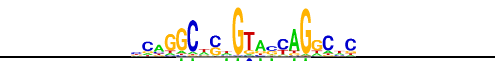
ZNF35	Control	C2H2 ZF	GHT-SELEX (Lysate)	RCade	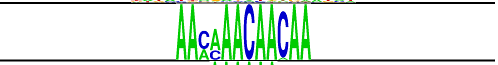
ZNF384	Control	C2H2 ZF	ChIP-seq	RCade	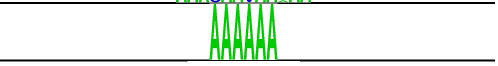
ZNF770	Control	C2H2 ZF	HT-SELEX (Lysate)	Dimont	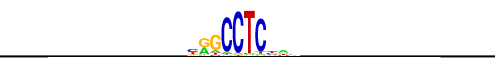
ZNF250	Control	C2H2 ZF	ChIP-seq	GkmSVM	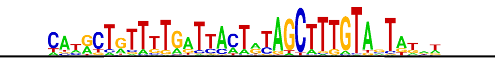
ZNF264	Control	C2H2 ZF	GHT-SELEX (Lysate)	ChIPMunk	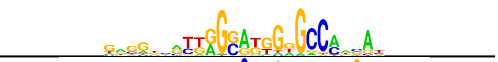
ZNF436	Control	C2H2 ZF	GHT-SELEX (Lysate)	RCade	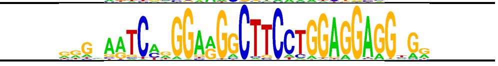
ZNF596	Control	C2H2 ZF	ChIP-seq	ChIPMunk	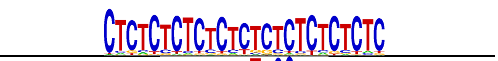
ZNF8	Control	C2H2 ZF	ChIP-seq	MEME	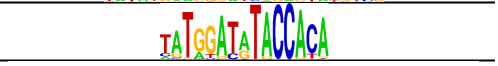
ZFP28	Control	C2H2 ZF	ChIP-seq	RCade	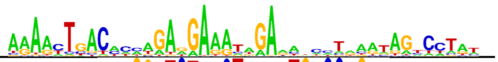
ZNF121	Control	C2H2 ZF	ChIP-seq	ExplaiNN	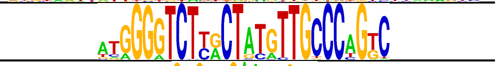
ZNF140	Control	C2H2 ZF	ChIP-seq	Streme	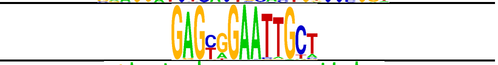
ZNF146	Control	C2H2 ZF	ChIP-seq	ChIPMunk	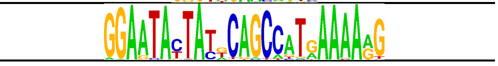
ZNF30	Control	C2H2 ZF	ChIP-seq	ChIPMunk	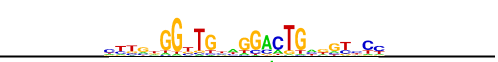
ZNF317	Control	C2H2 ZF	ChIP-seq	RCade	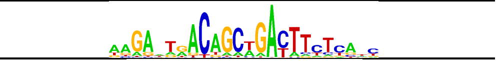
ZNF382	Control	C2H2 ZF	ChIP-seq	ChIPMunk	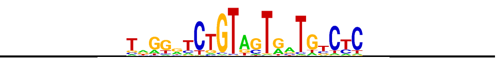
ZNF454	Control	C2H2 ZF	ChIP-seq	ExplaiNN	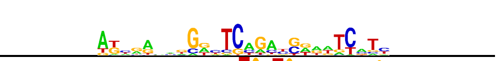
ZNF490	Control	C2H2 ZF	ChIP-seq	ChIPMunk	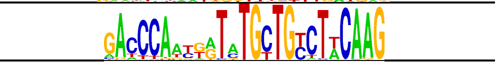
ZNF582	Control	C2H2 ZF	ChIP-seq	ChIPMunk	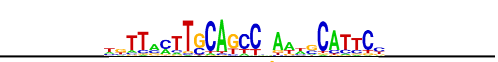
ZNF708	Control	C2H2 ZF	ChIP-seq	RCade	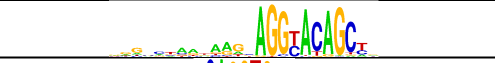
SNAI1	Control	C2H2 ZF	GHT-SELEX (IVT)	MEME	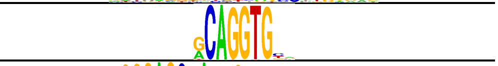
ZNF16	Control	C2H2 ZF	GHT-SELEX (Lysate)	ChIPMunk	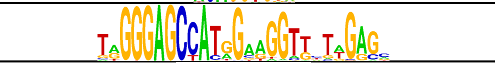
ZSCAN4	Control	C2H2 ZF	GHT-SELEX (IVT)	ProBound	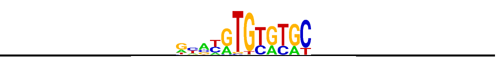
MAX	Control	bHLH	GHT-SELEX (Lysate)	Dimont	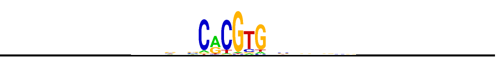

TF	Type	DBD type(s)	Motif source	Motif derivation algorithm	Motif
FOSL2	Control	bZIP	ChIP-seq	ChIPMunk	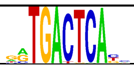
GCM1	Control	GCM	GHT-SELEX (IVT)	Dimont	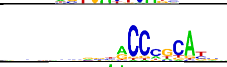
LEF1	Control	HMG/Sox	HT-SELEX (Lysate)	Autoseed	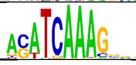
SOX2	Control	HMG/Sox	GHT-SELEX (Lysate)	Dimont	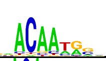
SRY	Control	HMG/Sox	HT-SELEX (Lysate)	Autoseed	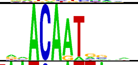
PAX7	Control	Homeodomain; Paired box	HT-SELEX (Lysate)	Streme	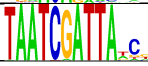
RORB	Control	Nuclear receptor	GHT-SELEX (Lysate)	Dimont	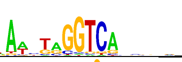
VDR	Control	Nuclear receptor	HT-SELEX (IVT)	Autoseed	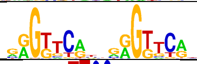
ELF3	Control	Ets; AT hook	ChIP-seq	MEME	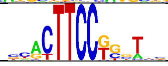
NFKB1	Control	Rel	GHT-SELEX (Lysate)	Dimont	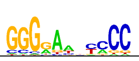
MYF6	Control	bHLH	GHT-SELEX (Lysate)	Dimont	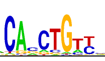
FLI1	Control	Ets	GHT-SELEX (IVT)	Dimont	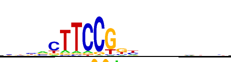
GABPA	Control	Ets	HT-SELEX (Lysate)	Autoseed	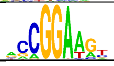
SOX15	Control	HMG/Sox	GHT-SELEX (Lysate)	Dimont	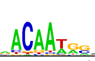
NR1H4	Control	Nuclear receptor	HT-SELEX (Lysate)	Autoseed	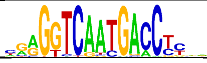
RFX5	Control	RFX	ChIP-seq	ChIPMunk	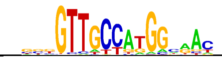
MGA	Control	T	GHT-SELEX (Lysate)	Dimont	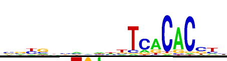
JUN	Control	bZIP	ChIP-seq	Streme	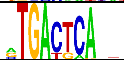
LHX6	Control	Homeodomain	ChIP-seq	Dimont	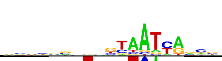
JUNB	Control	bZIP	ChIP-seq	MEME	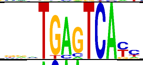
SOX5	Control	HMG/Sox	HT-SELEX (IVT)	ChIPMunk	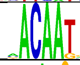
NR4A2	Control	Nuclear receptor	HT-SELEX (IVT)	ChIPMunk	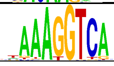
HMGA1	Control	AT hook	HT-SELEX (GFPIVT)	Dimont	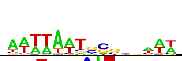
POU5F1	Control	Homeodomain; POU	GHT-SELEX (GFPIVT)	MEME	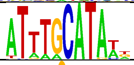
RARA	Control	Nuclear receptor	HT-SELEX (IVT)	ProBound	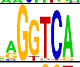
RXRA	Control	Nuclear receptor	HT-SELEX (Lysate)	MEME	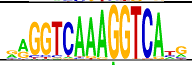
FIZ1	Codebook TF	C2H2 ZF	HT-SELEX (Lysate)	Autoseed	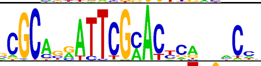
PRDM10	Codebook TF	C2H2 ZF	HT-SELEX (GFPIVT)	Autoseed	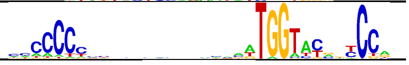
PRDM13	Codebook TF	C2H2 ZF	GHT-SELEX (Lysate)	Dimont	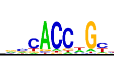
PRDM5	Codebook TF	C2H2 ZF	HT-SELEX (GFPIVT)	Homer	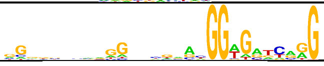
RLF	Codebook TF	C2H2 ZF	ChIP-seq	Dimont	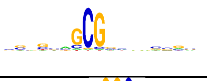
ZBTB8A	Codebook TF	C2H2 ZF	HT-SELEX (Lysate)	MEME	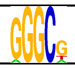

TF	Type	DBD type(s)	Motif source	Motif derivation algorithm	Motif
ZBTB8B	Codebook TF	C2H2 ZF	HT-SELEX (Lysate)	Homer	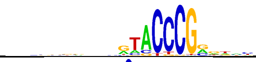
ZFAT	Codebook TF	C2H2 ZF	GHT-SELEX (GFPIVT)	ChIPMunk	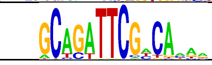
ZNF107	Codebook TF	C2H2 ZF	ChIP-seq	ChIPMunk	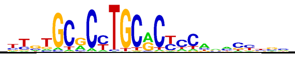
ZNF131	Codebook TF	C2H2 ZF	GHT-SELEX (Lysate)	Dimont	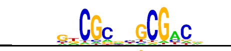
ZNF142	Codebook TF	C2H2 ZF	ChIP-seq	ChIPMunk	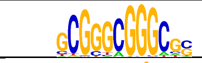
ZNF226	Codebook TF	C2H2 ZF	HT-SELEX (GFPIVT)	ChIPMunk	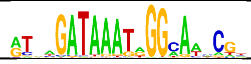
ZNF251	Codebook TF	C2H2 ZF	ChIP-seq	Dimont	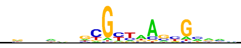
ZNF347	Codebook TF	C2H2 ZF	ChIP-seq	ChIPMunk	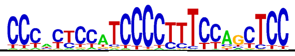
ZNF362	Codebook TF	C2H2 ZF	SMiLE-seq	Dimont	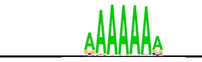
ZNF367	Codebook TF	C2H2 ZF	GHT-SELEX (GFPIVT)	Dimont	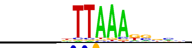
ZNF395	Codebook TF	C2H2 ZF	GHT-SELEX (IVT)	ProBound	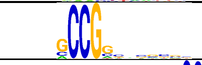
ZNF407	Codebook TF	C2H2 ZF	GHT-SELEX (Lysate)	ChIPMunk	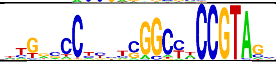
ZNF43	Codebook TF	C2H2 ZF	ChIP-seq	Dimont	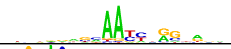
ZNF493	Codebook TF	C2H2 ZF	HT-SELEX (GFPIVT)	ChIPMunk	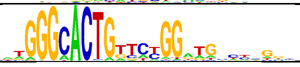
ZNF500	Codebook TF	C2H2 ZF	GHT-SELEX (GFPIVT)	Dimont	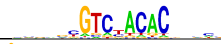
ZNF648	Codebook TF	C2H2 ZF	GHT-SELEX (GFPIVT)	Homer	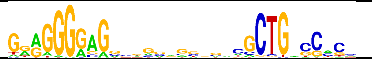
ZNF726	Codebook TF	C2H2 ZF	ChIP-seq	Dimont	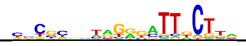
ZNF780B	Codebook TF	C2H2 ZF	GHT-SELEX (GFPIVT)	Dimont	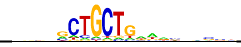
ZNF831	Codebook TF	C2H2 ZF	GHT-SELEX (GFPIVT)	Dimont	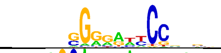
ZNF841	Codebook TF	C2H2 ZF	GHT-SELEX (GFPIVT)	Streme	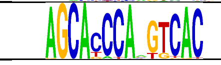
ZNF518B	Codebook TF	C2H2 ZF	GHT-SELEX (GFPIVT)	Dimont	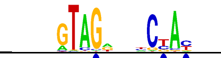
ZNF865	Codebook TF	C2H2 ZF	ChIP-seq	ExplaiNN	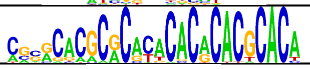
SALL3	Codebook TF	C2H2 ZF	HT-SELEX (Lysate)	ProBound	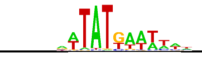
SLC2A4RG	Codebook TF	C2H2 ZF	GHT-SELEX (IVT)	Autoseed	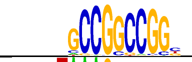
ZBTB40	Codebook TF	C2H2 ZF	HT-SELEX (GFPIVT)	Autoseed	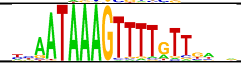
ZBTB41	Codebook TF	C2H2 ZF	GHT-SELEX (GFPIVT)	Dimont	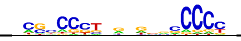
ZBTB47	Codebook TF	C2H2 ZF	GHT-SELEX (GFPIVT)	Dimont	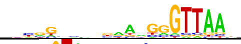
ZNF20	Codebook TF	C2H2 ZF	GHT-SELEX (GFPIVT)	Streme	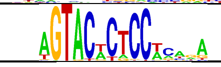
ZNF215	Codebook TF	C2H2 ZF	HT-SELEX (GFPIVT)	ProBound	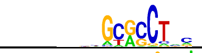
ZNF234	Codebook TF	C2H2 ZF	HT-SELEX (GFPIVT)	Autoseed	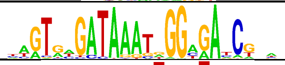
ZNF286B	Codebook TF	C2H2 ZF	HT-SELEX (GFPIVT)	Autoseed	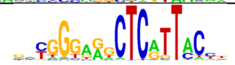
ZNF292	Codebook TF	C2H2 ZF	HT-SELEX (GFPIVT)	MEME	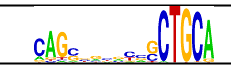

TF	Type	DBD type(s)	Motif source	Motif derivation algorithm	Motif
ZNF335	Codebook TF	C2H2 ZF	HT-SELEX (GFPIVT)	Autoseed	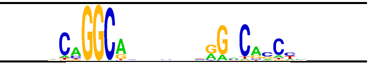
ZNF470	Codebook TF	C2H2 ZF	ChIP-seq	Dimont	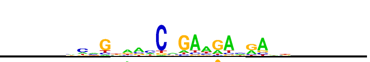
ZNF471	Codebook TF	C2H2 ZF	GHT-SELEX (GFPIVT)	MEME	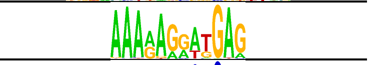
ZNF48	Codebook TF	C2H2 ZF	GHT-SELEX (GFPIVT)	ProBound	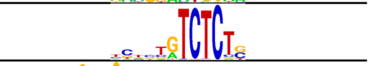
ZNF510	Codebook TF	C2H2 ZF	HT-SELEX (GFPIVT)	Autoseed	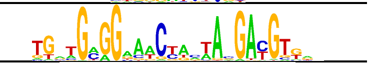
ZNF551	Codebook TF	C2H2 ZF	ChIP-seq	ChIPMunk	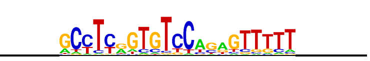
ZNF57	Codebook TF	C2H2 ZF	ChIP-seq	ChIPMunk	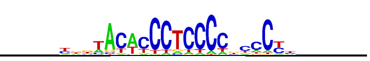
ZNF575	Codebook TF	C2H2 ZF	GHT-SELEX (GFPIVT)	ProBound	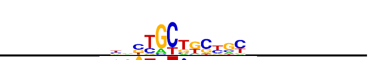
ZNF606	Codebook TF	C2H2 ZF	GHT-SELEX (GFPIVT)	Streme	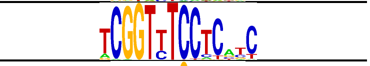
ZNF646	Codebook TF	C2H2 ZF	GHT-SELEX (IVT)	ProBound	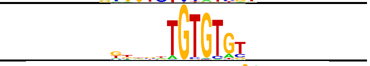
ZNF665	Codebook TF	C2H2 ZF	HT-SELEX (GFPIVT)	Autoseed	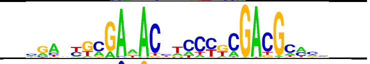
ZNF672	Codebook TF	C2H2 ZF	HT-SELEX (GFPIVT)	Autoseed	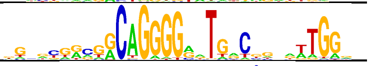
ZNF676	Codebook TF	C2H2 ZF	HT-SELEX (GFPIVT)	Autoseed	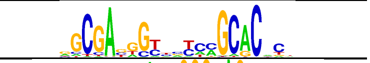
ZNF678	Codebook TF	C2H2 ZF	GHT-SELEX (GFPIVT)	ExplaiNN	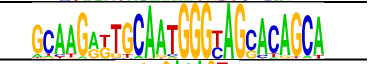
ZNF683	Codebook TF	C2H2 ZF	HT-SELEX (GFPIVT)	Streme	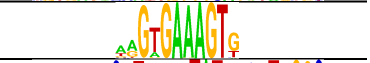
ZNF689	Codebook TF	C2H2 ZF	GHT-SELEX (GFPIVT)	ExplaiNN	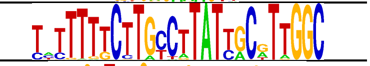
ZNF696	Codebook TF	C2H2 ZF	HT-SELEX (GFPIVT)	ChIPMunk	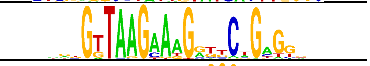
ZNF699	Codebook TF	C2H2 ZF	HT-SELEX (GFPIVT)	Autoseed	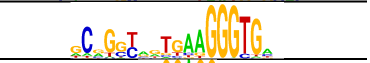
ZNF70	Codebook TF	C2H2 ZF	HT-SELEX (GFPIVT)	MEME	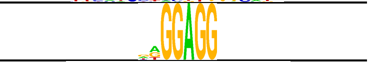
ZNF700	Codebook TF	C2H2 ZF	ChIP-seq	Dimont	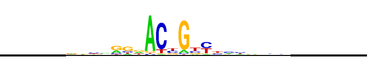
ZNF721	Codebook TF	C2H2 ZF	GHT-SELEX (GFPIVT)	Dimont	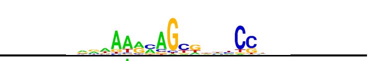
ZNF724	Codebook TF	C2H2 ZF	HT-SELEX (GFPIVT)	Autoseed	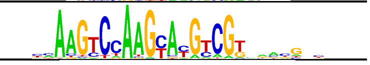
ZNF728	Codebook TF	C2H2 ZF	GHT-SELEX (GFPIVT)	ChIPMunk	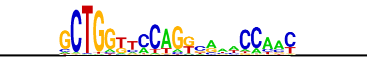
ZNF732	Codebook TF	C2H2 ZF	GHT-SELEX (GFPIVT)	Dimont	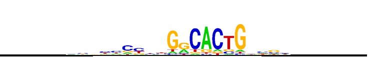
ZNF746	Codebook TF	C2H2 ZF	HT-SELEX (GFPIVT)	Dimont	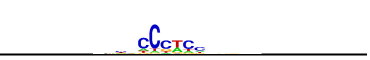
ZNF775	Codebook TF	C2H2 ZF	GHT-SELEX (GFPIVT)	Dimont	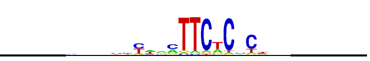
ZNF800	Codebook TF	C2H2 ZF	GHT-SELEX (GFPIVT)	Dimont	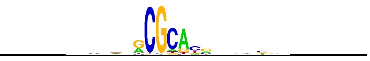
ZNF814	Codebook TF	C2H2 ZF	GHT-SELEX (GFPIVT)	Dimont	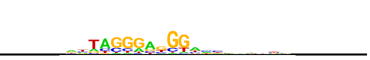
ZNF836	Codebook TF	C2H2 ZF	ChIP-seq	ChIPMunk	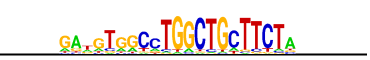
ZNF845	Codebook TF	C2H2 ZF	ChIP-seq	Dimont	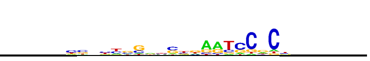
ZNF850	Codebook TF	C2H2 ZF	ChIP-seq	ChIPMunk	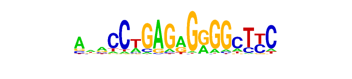
ZSCAN25	Codebook TF	C2H2 ZF	GHT-SELEX (Lysate)	Dimont	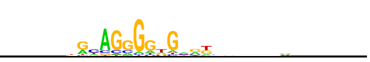

TF	Type	DBD type(s)	Motif source	Motif derivation algorithm	Motif
ZNF233	Codebook TF	C2H2 ZF	ChIP-seq	Dimont	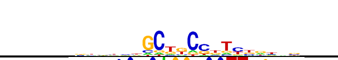
ZNF623	Codebook TF	C2H2 ZF	GHT-SELEX (GFPIVT)	ChIPMunk	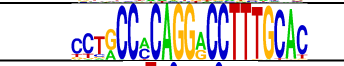
ZNF66	Codebook TF	C2H2 ZF	GHT-SELEX (GFPIVT)	Homer	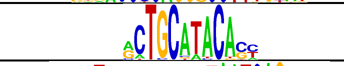
ZNF772	Codebook TF	C2H2 ZF	GHT-SELEX (GFPIVT)	ChIPMunk	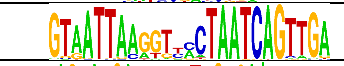
ZNF773	Codebook TF	C2H2 ZF	GHT-SELEX (GFPIVT)	ExplaiNN	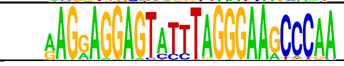
ZSCAN2	Codebook TF	C2H2 ZF	ChIP-seq	RCade	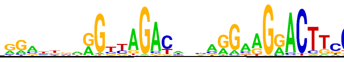
ZXDA	Codebook TF	C2H2 ZF	ChIP-seq	ChIPMunk	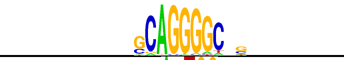
ZNF516	Codebook TF	C2H2 ZF	HT-SELEX (GFPIVT)	MEME	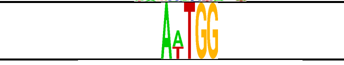
ZNF878	Codebook TF	C2H2 ZF	ChIP-seq	Dimont	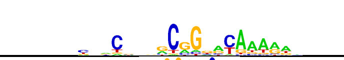
ZXDB	Codebook TF	C2H2 ZF	ChIP-seq	RCade	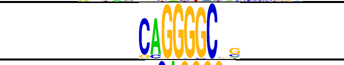
ZXDC	Codebook TF	C2H2 ZF	ChIP-seq	Homer	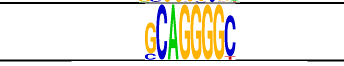
ZNF14	Codebook TF	C2H2 ZF	ChIP-seq	ChIPMunk	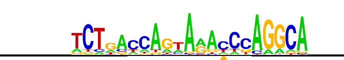
ZNF503	Codebook TF	C2H2 ZF	ChIP-seq	MEME	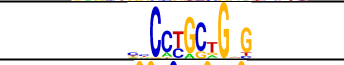
ZNF578	Codebook TF	C2H2 ZF	ChIP-seq	Homer	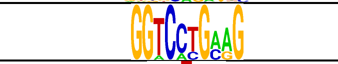
ZNF703	Codebook TF	C2H2 ZF	ChIP-seq	ChIPMunk	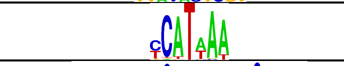
ZNF705E	Codebook TF	C2H2 ZF	ChIP-seq	ChIPMunk	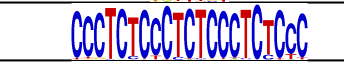
ZNF709	Codebook TF	C2H2 ZF	ChIP-seq	ChIPMunk	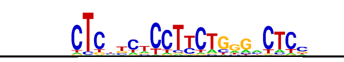
ZNF888	Codebook TF	C2H2 ZF	ChIP-seq	Dimont	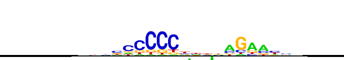
ZNF92	Codebook TF	C2H2 ZF	ChIP-seq	ChIPMunk	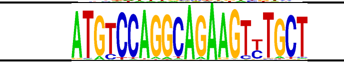
ZSCAN12	Codebook TF	C2H2 ZF	ChIP-seq	ChIPMunk	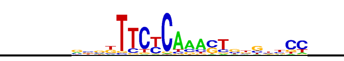
AC092835	Codebook TF	C2H2 ZF	ChIP-seq	ChIPMunk	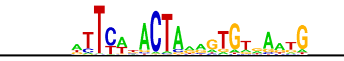
ATMIN	Codebook TF	C2H2 ZF	ChIP-seq	ExplaiNN	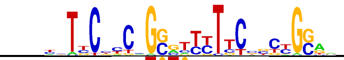
ZNF208	Codebook TF	C2H2 ZF	GHT-SELEX (GFPIVT)	Streme	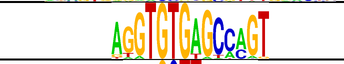
ZNF853	Codebook TF	C2H2 ZF	GHT-SELEX (GFPIVT)	ChIPMunk	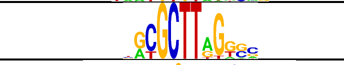
ZNF729	Codebook TF	C2H2 ZF	GHT-SELEX (GFPIVT)	ChIPMunk	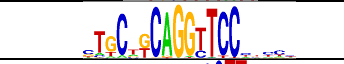
ZNF813	Codebook TF	C2H2 ZF	HT-SELEX (GFPIVT)	MEME	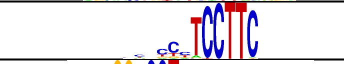
ZBTB24	Codebook TF	C2H2 ZF; AT hook	HT-SELEX (GFPIVT)	ChIPMunk	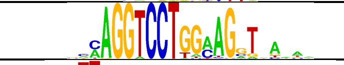
CASZ1	Codebook TF	C2H2 ZF	HT-SELEX (Lysate)	ChIPMunk	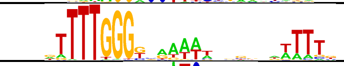
ZBTB5	Codebook TF	C2H2 ZF	SMiLE-seq	ChIPMunk	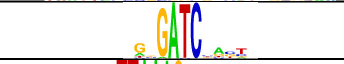
ZFP91	Codebook TF	C2H2 ZF	GHT-SELEX (GFPIVT)	Streme	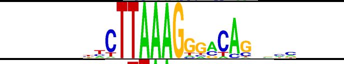
ZNF536	Codebook TF	C2H2 ZF	HT-SELEX (GFPIVT)	ChIPMunk	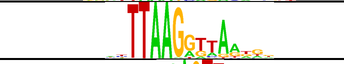
MYT1	Codebook TF	C2H2 ZF	HT-SELEX (Lysate)	MEME	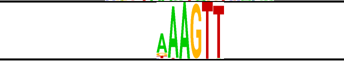

TF	Type	DBD type(s)	Motif source	Motif derivation algorithm	Motif
TSHZ2	Codebook TF	C2H2 ZF	HT-SELEX (IVT)	Dimont	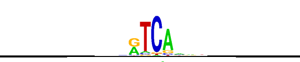
ZKSCAN4	Codebook TF	C2H2 ZF	GHT-SELEX (GFPIVT)	ChIPMunk	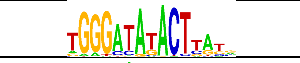
ZNF160	Codebook TF	C2H2 ZF	GHT-SELEX (GFPIVT)	ChIPMunk	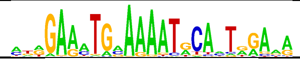
ZNF229	Codebook TF	C2H2 ZF	HT-SELEX (GFPIVT)	Autoseed	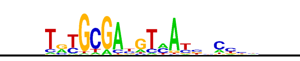
ZNF275	Codebook TF	C2H2 ZF	HT-SELEX (GFPIVT)	Autoseed	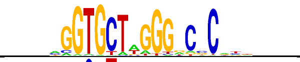
ZNF358	Codebook TF	C2H2 ZF	HT-SELEX (GFPIVT)	ChIPMunk	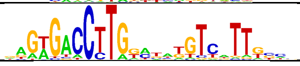
ZNF497	Codebook TF	C2H2 ZF	GHT-SELEX (GFPIVT)	ChIPMunk	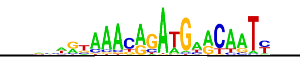
ZNF532	Codebook TF	C2H2 ZF	HT-SELEX (GFPIVT)	Dimont	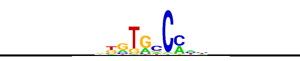
ZNF568	Codebook TF	C2H2 ZF	GHT-SELEX (GFPIVT)	Dimont	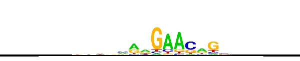
ZNF569	Codebook TF	C2H2 ZF	GHT-SELEX (GFPIVT)	Dimont	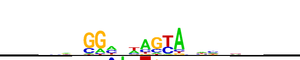
ZNF587B	Codebook TF	C2H2 ZF	HT-SELEX (GFPIVT)	ChIPMunk	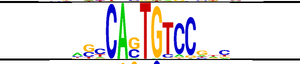
ZNF668	Codebook TF	C2H2 ZF	GHT-SELEX (GFPIVT)	ChIPMunk	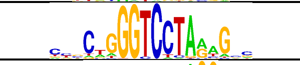
ZNF827	Codebook TF	C2H2 ZF	HT-SELEX (GFPIVT)	ProBound	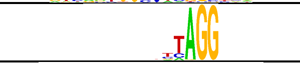
ZNF618	Codebook TF	C2H2 ZF	GHT-SELEX (Lysate)	ChIPMunk	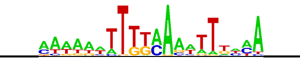
ZNF83	Codebook TF	C2H2 ZF	GHT-SELEX (GFPIVT)	Dimont	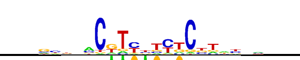
ZNF526	Codebook TF	C2H2 ZF	HT-SELEX (GFPIVT)	ChIPMunk	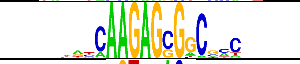
ZNF788P	Codebook TF	C2H2 ZF	HT-SELEX (GFPIVT)	Streme	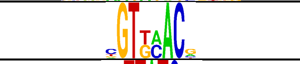
ZFPM1	Codebook TF	C2H2 ZF	SMiLE-seq	ChIPMunk	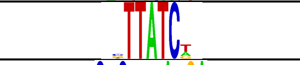
ZNF507	Codebook TF	C2H2 ZF	SMiLE-seq	Streme	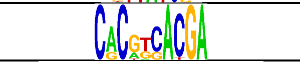
FAM200B	Codebook TF	BED ZF	ChIP-seq	Dimont	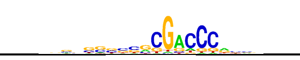
ZBED2	Codebook TF	BED ZF	GHT-SELEX (Lysate)	Dimont	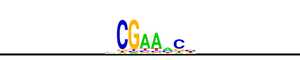
ZBED5	Codebook TF	BED ZF	GHT-SELEX (GFPIVT)	Dimont	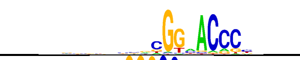
ZFTA	Codebook TF	BED ZF	ChIP-seq	MEME	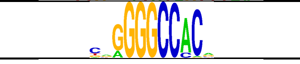
USF3	Codebook TF	bHLH	GHT-SELEX (Lysate)	Dimont	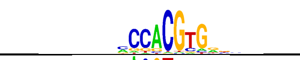
CREB3L3	Codebook TF	bZIP	GHT-SELEX (Lysate)	ChIPMunk	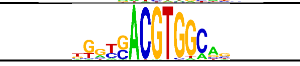
TIGD3	Codebook TF	CENPB	GHT-SELEX (Lysate)	Dimont	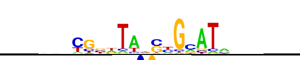
CXXC4	Codebook TF	CxxC	HT-SELEX (IVT)	Autoseed	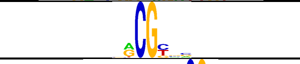
FBXL19	Codebook TF	CxxC	GHT-SELEX (Lysate)	ProBound	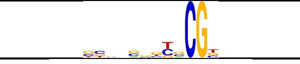
TET3	Codebook TF	CxxC	HT-SELEX (IVT)	Dimont	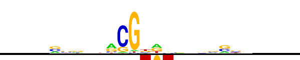
MKX	Codebook TF	Homeodomain	HT-SELEX (GFPIVT)	MEME	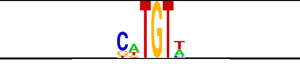
TPRX1	Codebook TF	Homeodomain	HT-SELEX (GFPIVT)	Dimont	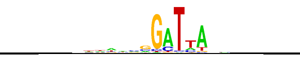
MSANTD1	Codebook TF	MADF	GHT-SELEX (Lysate)	Dimont	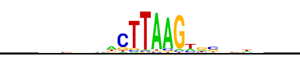

TF	Type	DBD type(s)	Motif source	Motif derivation algorithm	Motif
DMTF1	Codebook TF	Myb/SANT	GHT-SELEX (GFPIVT)	Dimont	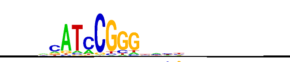
SP140	Codebook TF	SAND	PBM	Dimont	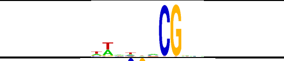
SP140L	Codebook TF	SAND	SMiLE-seq	ProBound	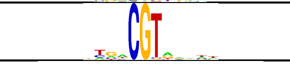
TIGD4	Codebook TF	CENPB	GHT-SELEX (Lysate)	Dimont	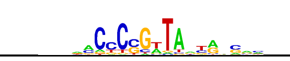
CAMTA1	Codebook TF	CG	GHT-SELEX (GFPIVT)	Dimont	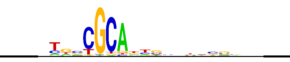
CAMTA2	Codebook TF	CG	GHT-SELEX (Lysate)	Dimont	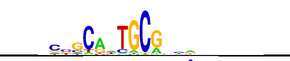
SP100	Codebook TF	SAND	GHT-SELEX (GFPIVT)	ProBound	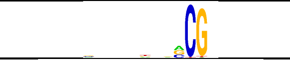
MYPOP	Codebook TF	Myb/SANT	GHT-SELEX (Lysate)	Dimont	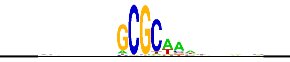
TERF1	Codebook TF	Myb/SANT	GHT-SELEX (Lysate)	Dimont	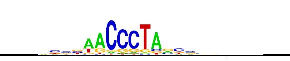
DNTTIP1	Codebook TF	AT hook	HT-SELEX (Lysate)	Dimont	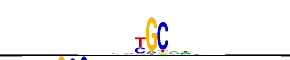
ZBED9	Codebook TF	BED ZF	HT-SELEX (Lysate)	ChIPMunk	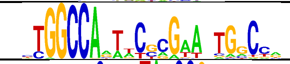
JRK	Codebook TF	CENPB	HT-SELEX (GFPIVT)	ChIPMunk	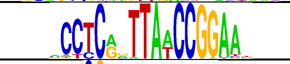
KDM2A	Codebook TF	CxxC	GHT-SELEX (Lysate)	ProBound	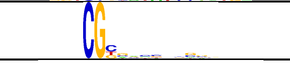
FLYWCH1	Codebook TF	FLYWCH	ChIP-seq	Dimont	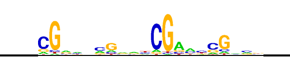
LEUTX	Codebook TF	Homeodomain	GHT-SELEX (GFPIVT)	Dimont	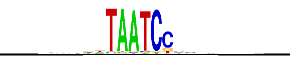
BATF2	Codebook TF	bZIP	HT-SELEX (Lysate)	ProBound	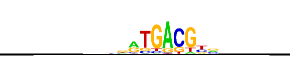
MYRFL	Codebook TF	Ndt80/PhoG	ChIP-seq	ChIPMunk	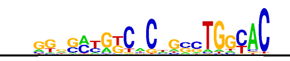
MBD1	Codebook TF	MBD; CxxC ZF	ChIP-seq	Dimont	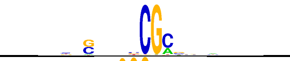
GTF2IRD2	Codebook TF	GTF2I	HT-SELEX (Lysate)	ChIPMunk	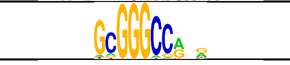
BHLHA9	Codebook TF	bHLH	ChIP-seq	Dimont	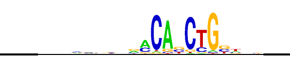
MBD3	Codebook TF	MBD	ChIP-seq	Dimont	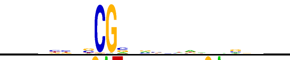
MYSM1	Codebook TF	Myb/SANT	ChIP-seq	Streme	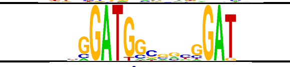
TTF1	Codebook TF	Myb/SANT	ChIP-seq	ChIPMunk	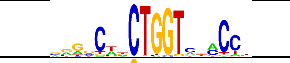
ZBED4	Codebook TF	BED ZF	SMiLE-seq	Homer	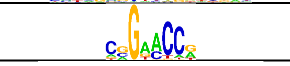
TIGD7	Codebook TF	CENPB	GHT-SELEX (GFPIVT)	Dimont	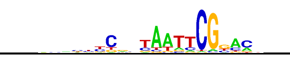
MSANTD4	Codebook TF	Myb/SANT	GHT-SELEX (Lysate)	Dimont	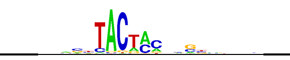
TIGD5	Codebook TF	CENPB	GHT-SELEX (GFPIVT)	Dimont	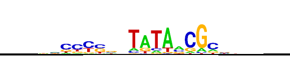
ZGLP1	Codebook TF	GATA	HT-SELEX (Lysate)	Homer	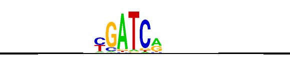
GATAD2A	Codebook TF	GATA	GHT-SELEX (GFPIVT)	Dimont	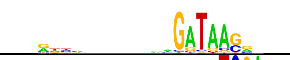
POGK	Codebook TF	Brinker	HT-SELEX (IVT)	ChIPMunk	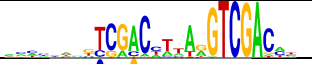
GRHL3	Codebook TF	Grainyhead	HT-SELEX (IVT)	ChIPMunk	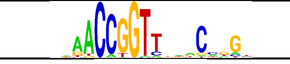
HMG20A	Codebook TF	HMG/Sox	HT-SELEX (GFPIVT)	Streme	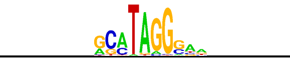

TF	Type	DBD type(s)	Motif source	Motif derivation algorithm	Motif
AHCTF1	Codebook TF	AT hook	PBM	Dimont	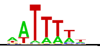
GLYR1	Codebook TF	AT hook	PBM	Dimont	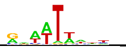
PHF21A	Codebook TF	AT hook	PBM	Dimont	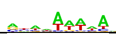
SETBP1	Codebook TF	AT hook	PBM	Dimont	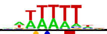
MBNL2	Codebook TF	CCCH ZF	PBM	ChIPMunk	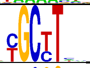
CGGBP1	Codebook TF	Unknown	HT-SELEX (Lysate)	MEME	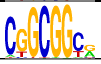
NACC2	Codebook TF	Unknown	GHT-SELEX (GFPIVT)	Dimont	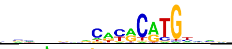
DACH1	Codebook TF	Unknown	HT-SELEX (GFPIVT)	ChIPMunk	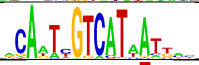
DACH2	Codebook TF	Unknown	HT-SELEX (IVT)	Autoseed	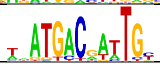
PURB	Codebook TF	Unknown	PBM	ExplaiNN	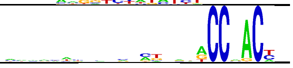
TCF20	Codebook TF	Unknown	PBM	Dimont	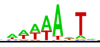
